# Meta-analysis of 46,000 germline *de novo* mutations linked to human inherited disease

**DOI:** 10.1186/s40246-024-00587-8

**Published:** 2024-02-23

**Authors:** Mónica Lopes-Marques, Matthew Mort, João Carneiro, António Azevedo, Andreia P. Amaro, David N. Cooper, Luísa Azevedo

**Affiliations:** 1 CIIMAR-Interdisciplinary Centre of Marine and Environmental Research, University of Porto, Porto, Portugal; 2 Institute of Medical Genetics, School of Medicine, Cardiff University, Cardiff, UK; 3 CHUdSA-Centro Hospitalar Universitário de Santo António, Porto, Portugal; 4 UMIB-Unit for Multidisciplinary Research in Biomedicine, ICBAS - School of Medicine and Biomedical Sciences, University of Porto, Porto, Portugal; 5 ITR - Laboratory for Integrative and Translational Research in Population Health, Porto, Portugal

**Keywords:** *De novo* mutations (DNMs), Genetic disease, Development, Neurodevelopmental disorders, Psychiatric disorders, Autism spectrum disorder

## Abstract

**Background:**

*De novo* mutations (DNMs) are variants that occur anew in the offspring of noncarrier parents. They are not inherited from either parent but rather result from endogenous mutational processes involving errors of DNA repair/replication. These spontaneous errors play a significant role in the causation of genetic disorders, and their importance in the context of molecular diagnostic medicine has become steadily more apparent as more DNMs have been reported in the literature. In this study, we examined 46,489 disease-associated DNMs annotated by the Human Gene Mutation Database (HGMD) to ascertain their distribution across gene and disease categories.

**Results:**

Most disease-associated DNMs reported to date are found to be associated with developmental and psychiatric disorders, a reflection of the focus of sequencing efforts over the last decade. Of the 13,277 human genes in which DNMs have so far been found, the top-10 genes with the highest proportions of DNM relative to gene size were *H3-3 A, DDX3X, CSNK2B, PURA, ZC4H2, STXBP1, SCN1A, SATB2, H3-3B* and *TUBA1A.* The distribution of CADD and REVEL scores for both disease-associated DNMs and those mutations not reported to be *de novo* revealed a trend towards higher deleteriousness for DNMs, consistent with the likely lower selection pressure impacting them. This contrasts with the non-DNMs, which are presumed to have been subject to continuous negative selection over multiple generations.

**Conclusion:**

This meta-analysis provides important information on the occurrence and distribution of disease-associated DNMs in association with heritable disease and should make a significant contribution to our understanding of this major type of mutation.

**Supplementary Information:**

The online version contains supplementary material available at 10.1186/s40246-024-00587-8.

## Background

*De novo* mutations (DNMs) challenge traditional notions of Mendelian inheritance because the parents of affected offspring bearing DNMs are not themselves carriers [1–6]. In recent years, increasing numbers of DNMs have been identified as a consequence of the widespread adoption of whole exome/genome sequencing to screen patient cohorts.

In principle, there are two junctures at which such mutations can arise: (1) during gametogenesis in one of the parents, or (2) during the early divisions of embryogenesis. In the former instance, the mutation occurs in the germline of one of the parents and there is a tendency for the germline mutation rate to increase with age in both males and females [7–11], although DNMs originate more frequently in the paternal germline due to the comparatively high number of cell divisions occurring during spermatogenesis [6]. In the latter instance, by dint of their occurrence post-fertilization, the mutations are termed postzygotic DNMs [12]. The precise timepoint at which a mutation occurs during embryonic development is important for the establishment of the somatic mutational distribution pattern. Thus, if the mutation arises prior to primordial germline cell specification, it can be transmitted through the germline, resulting in recurrence of the disease in the next generation [13]. By contrast, if it arises after primordial germline cell specification, it will give rise to either mosaicism in the germline (which has the potential to result in disease recurrence) or mosaicism in the somatic tissues [8]. In contradistinction to germline mutations where paternal age has a considerable influence on the mutation rate [8, 14–16], currently available data are consistent with the absence of any parent-of-origin bias in relation to postzygotic mutations [17].

DNMs arise mainly through the action of endogenous processes mediated by the specific features and intrinsic properties of the genomic DNA sequence (e.g. methylation-mediated deamination of 5-methylcytosine, DNA sequence repetitivity, GC content, non-B DNA structures, recombination hotspots), chromosomal architecture (e.g. chromatin structure and interactions) and replication/repair errors [3, 17–19].

Our study, based on a large collection of germline DNMs, has explored the impact of these lesions on human inherited disease, with the specific aim of understanding their distribution and their key role in increasing the incidence of such disorders.

## Methods

### DNM dataset

A total of 443,508 germline disease-associated mutations (annotated as DM, DM?, DP and DFP [20]) were sourced from the Human Gene Mutation Database (HGMD Professional v.2023.2), which includes a set of 46,489 putatively disease-causing DNMs from 13,277 genes. This constitutes a highly reliable source of germline DNMs due to the manual curation of the scientific literature related to human inherited disease [21]. Mutations were included in this DNM set if they were classified as “disease-causing mutations” (DM) or “probable/possible pathogenic mutations” (DM?) and had been annotated as DNMs by HGMD (reflecting the claims made by the authors in the original articles reporting them). The only exception was the prediction of the deleteriousness (described below) in which only DM were included.

### Mapping of disease terms onto the Unified Medical Language System (UMLS)

Categorization of the disease-associated DNM set into high level disease concepts (e.g. developmental disorders or immune system disorders) was based on the Unified Medical Language System (UMLS) annotations [22] using a simple word permutation-based method. The disease names were mapped to UMLS concept identifiers (CUI) using the open source UMLS-Query module [23]. UMLS-Query provides a function called maptoId, which accepts a phrase and maps it to a CUI. A total of 39,125 (approx. 84% of the total) disease terms relating to DNMs were mapped to the UMLS with high confidence. The hierarchy of disease terms from the UMLS ontology was used to explore the relationships between the disease classes and DNMs. Using graph traversal in the UMLS Metathesaurus, a DNM could possibly (if appropriate) be associated with multiple high level disease classes (e.g. Primary sclerosing cholangitis is classed both as an ‘immune’ disorder and as a ‘digestive system’ disorder).

### DNM enrichment analysis and Gene Ontology (GO) enrichment analysis

To identify disease genes enriched for DNMs, a relative DNM enrichment rate was calculated. The relative DNM enrichment rate allows for intergenic differences in coding sequence length and DNM frequency between specific genes to be taken into account and is defined as the fraction of the observed number of DNMs normalised with respect to the coding sequence length calculated on a gene wise basis:

$$Relative\, mutability\, of\, DNMs=\frac{Number\, of\, DNMs\, for\, gene}{Coding\, sequence\, length\, of\, gene\, \left(bp\right)}$$

Of the 13,249 genes (out of 13,277) from the DNM mutation set for which transcript information was available, we excluded genes with fewer than 5 DNMs (arbitrary cut-off; this excluded 11,105). For the remaining genes, the mean + 1SD values of relative mutability were calculated (0.424 + 0.615 = 1.039), so that only genes with a DNM enrichment rate greater than the mean + 1SD were included in the analyses (*N* = 187). For these genes, we also normalized the frequency of disease-associated *de novo* mutations by the estimates of the per gene mutations rates previously reported by Bethune and collaborators [24]. For this, the “expected_genovo_missense_corrected” values were used as missense variants represent the vast majority of DNM among the 187 enriched genes. The subset of 187 genes was then used for the analysis of biological processes using the DAVID Gene Ontology (GO) tool (https://david.ncifcrf.gov/).

### Prediction of the functional impact of missense mutations

To predict the functional impact of mutations, the tool CADD (Combined Annotation Dependent Depletion) was employed [25]. The datasets were both normalised with respect to mutation type by selecting only missense mutations from each dataset. CADD predictions were calculated on two sets of HGMD missense disease-causing mutations (only DM mutations were included), viz. 5,307 mutations from the DNM set and 32,605 disease-causing mutations from HGMD (non-DNMs). This functional impact analysis was then repeated by using REVEL (Rare Exome Variant Ensemble Learner) prediction scores [26]. REVEL prediction scores were available for 5,506 mutations in the HGMD missense disease-causing DNM and 33,191 disease-causing non-DNMs from HGMD.

The above-mentioned strategy is graphically shown in Additional file 1.

## Results

### Frequency and distribution of DNM types among disease-associated mutations

**Fig. 1:**
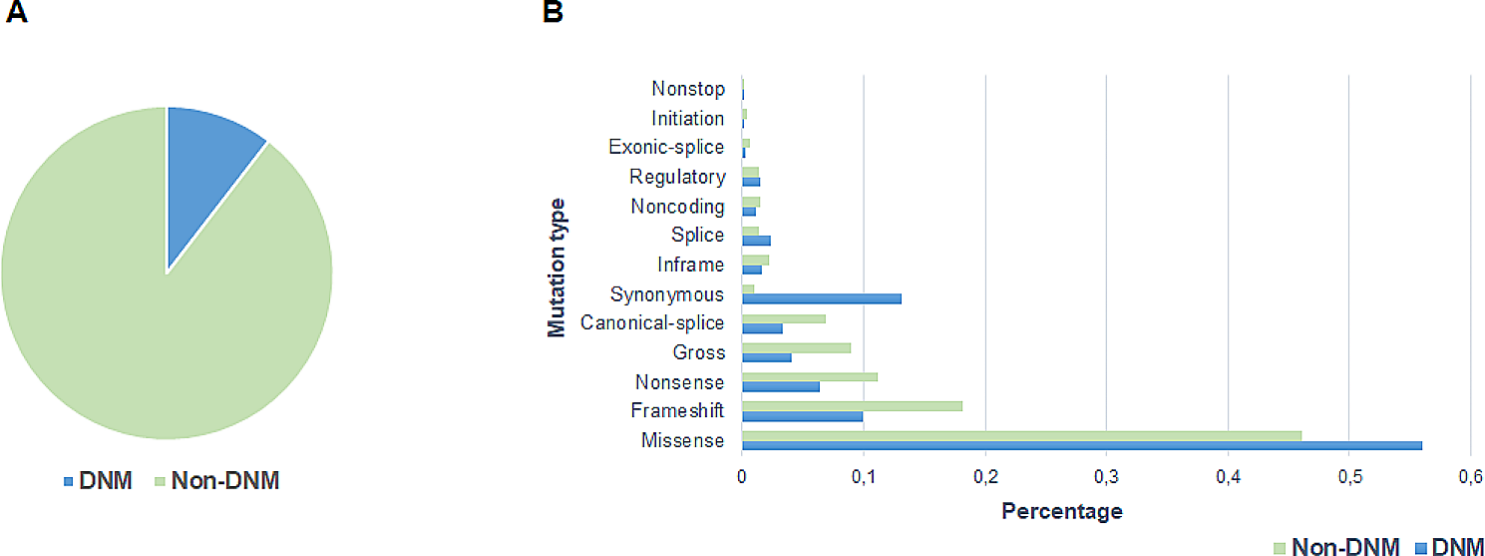
Proportion of DNMs and non-DNMs in HGMD (**A**) and distribution of mutation types for both DNMs and non-DNMs (**B**)

A total of 443,508 germline disease-associated mutations were obtained from HGMD and subsequently analysed. Of these, 46,489 were identified as DNMs (from author-provided information), representing 10.5% of the total number of mutations in the sample (Fig. 1A). Missense replacements were found to be the most common type of mutation among both DNMs and disease-associated DNMs not reported be *de novo* (non-DNMs), accounting for 56% and 46% of the listed mutations, respectively (Fig. 1B). One potentially interesting finding was the higher proportion of synonymous replacements noted among DNMs (13%) compared to just 1% for non-DNMs. Although this difference was statistically significant (χ^2^ (1, *N* = 443,508) = 3469, *p* < 0.01), it is likely to be artefactual, simply reflecting the criteria used for identifying and including DNMs rather than the underlying mechanisms driving these replacements. In the absence of mRNA phenotyping data, synonymous substitutions would normally be excluded from HGMD because there would be no direct and cogent evidence for their pathogenicity. By contrast, synonymous substitutions that occurred *de novo* would probably have been prioritized by the reporting authors because of the focus on DNMs being of pathological significance in the context of the various neurodevelopmental disorders under study. At the same time one cannot exclude the possibility that pathogenic synonymous substitutions would tend to be under ascertained in the context of non-DNMs as they often tend to go unreported in the context of molecular diagnostic testing.

### Distribution of DNMs between disease concepts

**Fig. 2:**
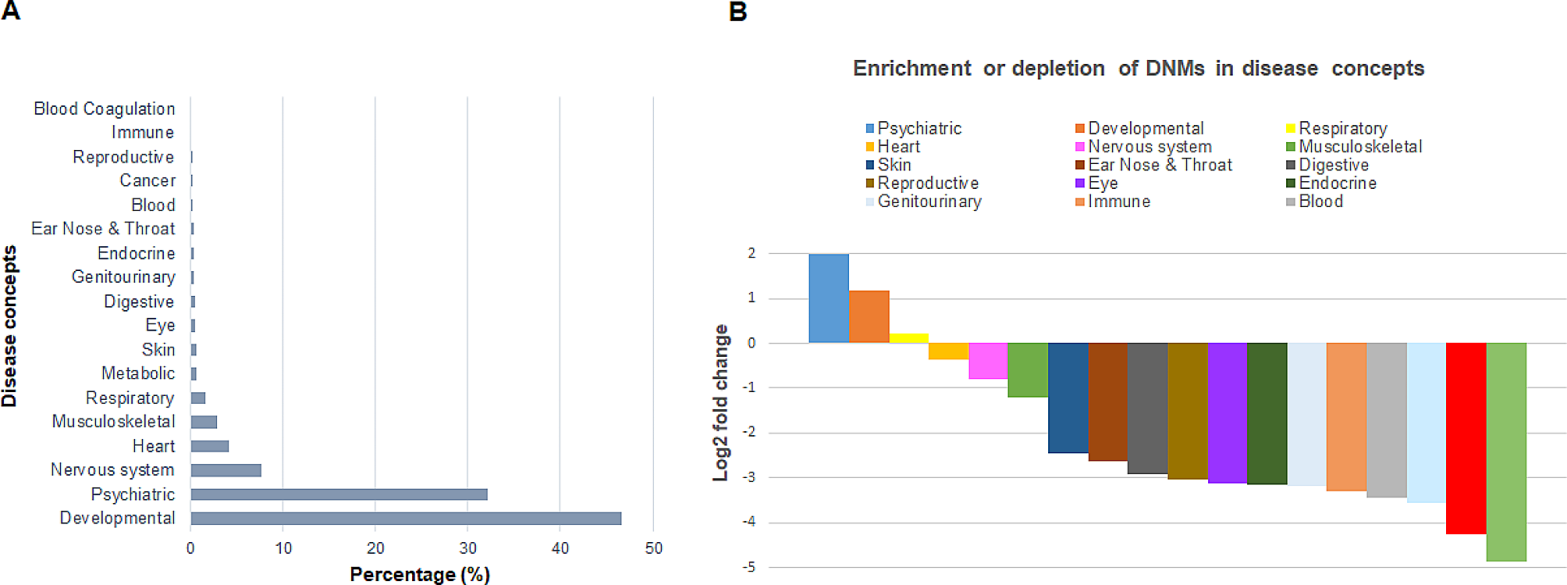
Distribution of DNMs by disease concepts (**A**). Enrichment or depletion of DNMs in disease concepts (**B**)

Figure 2 presents the findings when UMLS disease concepts [22] were utilized to categorize the 46,489 DNMs annotated by HGMD. The majority of DNMs occurred in genes belonging to two predominant classificatory categories: “Developmental” disorders, accounting for 47% of DNMs, and “Psychiatric” disorders, comprising 32% of DNMs (Fig. 2A). It is important to note that owing to the nature of the inclusion criteria (by mapping DNMs to multiple high level classes for each disease concept), a single disease may be classified under multiple categories, resulting in overlaps between concepts. Nevertheless, the high prevalence of DNMs among developmental and psychiatric diseases is clear. In agreement with this assertion, the enrichment analysis (Fig. 2B) revealed log2-fold changes of 2 and 1.2 for psychiatric and developmental concepts, respectively, highlighting a clear association between DNMs and these conditions that may have resulted, at least in part, from the considerable efforts that have been undertaken in recent years to unravel their genetic basis by whole genome sequencing or whole exome sequencing methodologies [3, 17, 27–35].

**Fig. 3:**
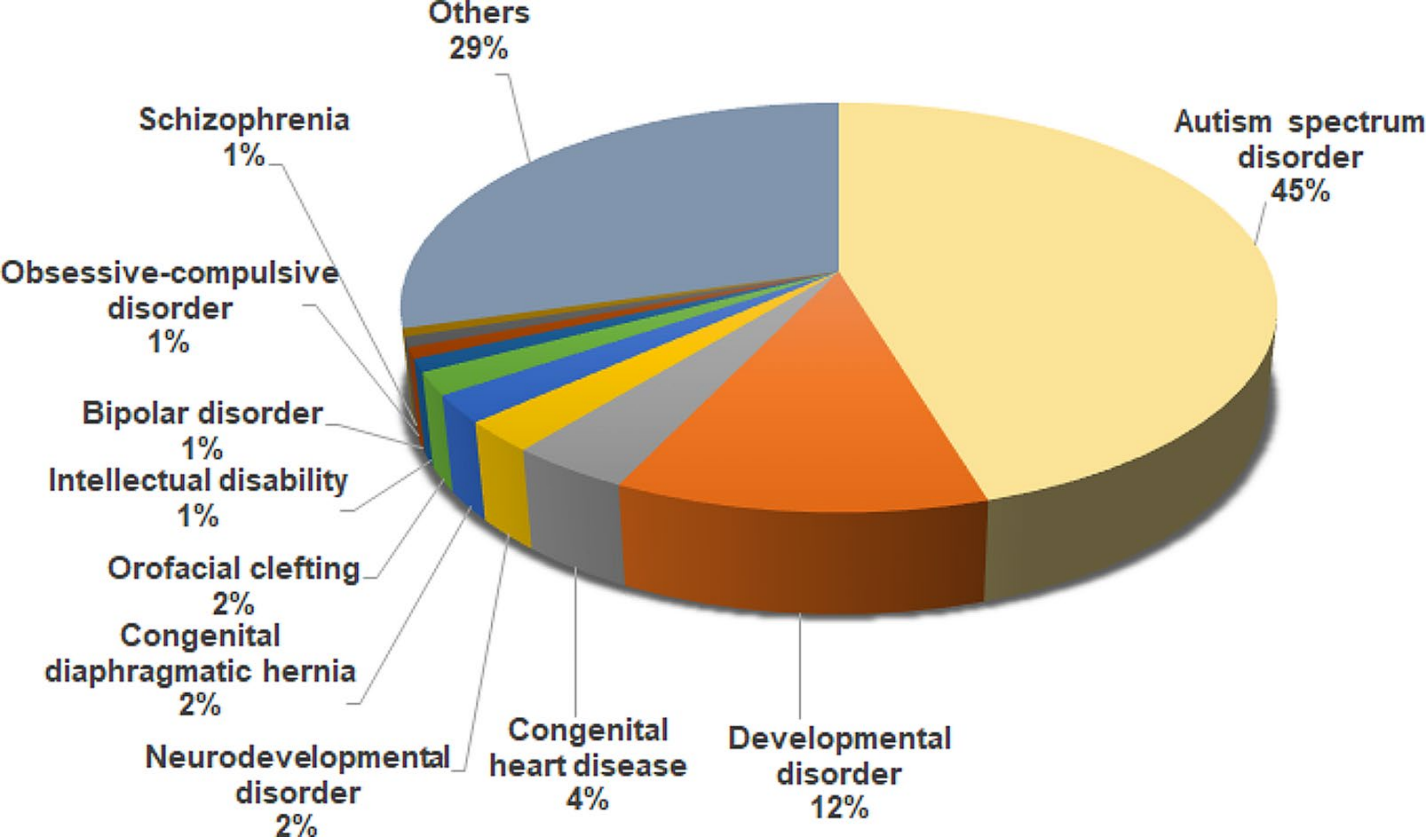
Genetic diseases with the highest proportion of DNMs

Next, the DNMs dataset was interrogated by disease term (Fig. 3). The most frequent term obtained was ‘autism’ reaching 45% of all DNMs. Autism spectrum disorder (ASD), the most frequent neurodevelopmental disorder in Western populations, is characterized by impaired social communication and interactions, and repetitive behavior [36]. The incidence of ASD has been estimated to be 60.38 × 10^4^ according to the Global Burden of Disease Study 2019 [37]. In terms of the molecular basis of autism spectrum disorders, and according to previous estimates, DNMs account for approximately one third of all cases ascertained [38]. This high proportion is probably due to a high proportion of DNMs being anticipated in ASD cohorts and because identifying a DNM in an individual with autism is generally held to be supportive of pathological authenticity (although by the very nature of this approach, there will probably also be a considerable number of false positives).

Congenital heart disease is another multi-gene phenotype that exhibits a high proportion of DNMs, with approximately 4% of all DNMs in our dataset associated with this condition, Other congenital phenotypes, such as orofacial clefting and congenital diaphragmatic hernia, are also represented at a relatively high level in our dataset, each accounting for 2% of DNMs. These figures might reflect the fact that these birth defects are not only frequent in human populations but also that they have come under close molecular scrutiny by whole exome/genome sequencing in recent years [39–43]. About 29% of DNMs tagged in our analyses as belonging to the “Others” category, the disease terms with the highest number of DNMs were: developmental and epileptic encephalopathy, hydrocephalus, epilepsy, neurofibromatosis type 1, Dravet syndrome, Tourette syndrome, Coffin-Siris syndrome, Tetralogy of Fallot, periventricular nodular heterotopia and KBG syndrome.

### Distribution of DNMs between and among disease-associated genes

Next, we examined the genes that harbored the highest numbers of disease-associated DNMs. The 20 genes with the highest number of DNMs accounted for only 5.8% of all the DNMs in our dataset (Additional file 2). The gene with the highest reported number of DNMs was *SCN1A* which encodes the sodium voltage-gated channel alpha subunit 1 involved in severe myoclonic epilepsy of infancy or Dravet syndrome [44, 45]. DNMs in the *SCN1A* gene have been reported as a major cause of this disease [46, 47]. The second most common occurrence was observed for *ARID1B*, one of the genes underlying Coffin-Siris syndrome [48, 49]. It encodes a component of the SWI/SNF (BAF) chromatin remodeling complex which is essential for gene expression during development [50]. The *NF1* gene, known for some time to have a high mutation rate [51, 52], has one of the highest numbers of DNMs. This gene is responsible for neurofibromatosis type 1, a common autosomal dominant tumor predisposition syndrome [53–55], in which approximately half of the cases are caused by DNMs [56].

Two highly penetrant autism spectrum disorder genes [35], *SCN2A* and *SHANK3*, are represented among the top 20 genes with the highest number of DNMs. In addition, many of the genes shown in Additional file 2 (e.g. *SCN1A*, *ANKRD11*, *KMT2A*, *SYNGAP1*, *SATB2*, *CHD7*, *STXBP1*, *SHANK3*) have been shown to be associated with autism and other neurodevelopmental phenotypes (e.g. [57–63]). Because neurodevelopmental disorders share genetic risk genes and variants (inherited and *de novo*), they have been postulated to represent a continuum of etiological and genetic factors [64–66]. In fact, Ghiania and Faudez have proposed that impairments of specific windows of vulnerability during brain development may result in distinct disease entities with overlapping clinical symptoms [67].

Because gene complexity can contribute to the high number of mutations in any given gene, we investigated it by normalizing the number of DNMs by the coding length of the 187 genes enriched in DNMs (Table 1). To further contextualize our findings, we used estimates of per-gene mutation rates from Bethune and collaborators [24]. Although the coverage among the 187 genes was incomplete, we nevertheless observed a strong correlation between the two datasets (Additional file 2). Among the genes presented in Table 1, five (*DDX3X*, *STXBP1*, *SCN1A*, *SATB2*, *CTNNB1*) overlap with the top 20 genes with the highest number of DNMs (Additional file 2). This finding is consistent with previous research that has established a correlation between longer transcripts and genes that play a functional role at early developmental stages [68]. It is also important to note that genes associated with other phenotypes, such as the *SLC35A2* gene, associated with an inborn error of metabolism [2], are among the genes with the high proportions of DNMs.

**Table 1:** Top 20 genes with the highest proportion of DNMs

Gene Symbol	Relative mutability (DNM frequency/coding length)*100	Most frequent clinical phenotype (HGMD)
*H3-3 A*	7.056	Neurodegenerative disease
*DDX3X*	6.747	DDX3X syndrome
*CSNK2B*	6.636	Poirier-Bienvenu neurodevelopmental syndrome
*PURA*	6.295	PURA syndrome
*ZC4H2*	5.926	Arthrogryposis multiplex congenita
*STXBP1*	5.243	Epileptic encephalopathy, early infantile
*SCN1A*	5.158	Dravet syndrome
*SATB2*	4.723	SATB2-associated syndrome
*H3-3B*	4.623	Neurodegenerative disease
*TUBA1A*	4.425	Tubulinopathy
*SLC35A2*	4.146	Congenital disorder of glycosylation
*CTNNB1*	4.007	Neurodevelopmental disorder
*VAMP2*	3.989	Axial hypotonia, intellectual disability and autism
*GNAO1*	3.944	Encephalopathy
*MECP2*	3.901	Rett syndrome
*DYRK1A*	3.665	Intellectual disability/Developmental disorder
*GATAD2B*	3.591	Neurodevelopmental disorder
*WDR45*	3.591	Neurodegeneration with brain iron accumulation
*SLC6A1*	3.556	Neurodevelopmental disorder
*H1-4*	3.333	Developmental disorder/Intellectual disability and distinct facial gestalt

### GO enrichment analysis

We performed a GO analysis on biological processes for 187 disease genes enriched for DNMs (Additional file 2). This GO analysis identified DNM-enriched disease genes as being significantly enriched in 190 different types of biological process (e.g. system development or transcription related processes) (Additional file 3). The top 10 enriched clusters are shown in Table 2. The term GO:0048731 refers to system development which is the category that embraces a multitude of processes that together contribute to the formation and growth of an individual. It comprises not only nervous system development, (GO:0007399 with an enrichment of 3.7), but all other physiological systems. Other enriched GO terms are related to the regulation of transcription (GO:0045893, GO:1,903,508, GO:1,902,680, GO:0044767, GO:0010628).

**Table 2:** Biological processes for 187 genes enriched in DNMs

Gene Ontology Term	Number of genes	Fold enrichment	*P* value	FDR
GO:0048731 system development	124	2.548578	3.01E-31	9.52E-28
GO:0007275 multicellular organism development	128	2.449113	4.52E-31	9.52E-28
GO:0007399 nervous system development	89	3.659853	1.31E-30	1.84E-27
GO:0044707 single-multicellular organism process	136	2.152079	3.94E-28	4.15E-25
GO:0048856 anatomical structure development	130	2.234834	1.25E-27	1.01E-24
GO:0045893 positive regulation of transcription, DNA-templated	71	4.31298	1.68E-27	1.01E-24
GO:1,903,508 positive regulation of nucleic acid-templated transcription	71	4.31298	1.68E-27	1.01E-24
GO:1,902,680 positive regulation of RNA biosynthetic process	71	4.290988	2.28E-27	1.19E-24
GO:0044767 single-organism developmental process	134	2.150094	2.53E-27	1.19E-24
GO:0010628 positive regulation of gene expression	79	3.696033	1.05E-26	4.43E-24

### Is there a tendency for pathogenic DNMs to be more deleterious than pathogenic mutations not reported to be *de novo*?

**Fig. 4:**
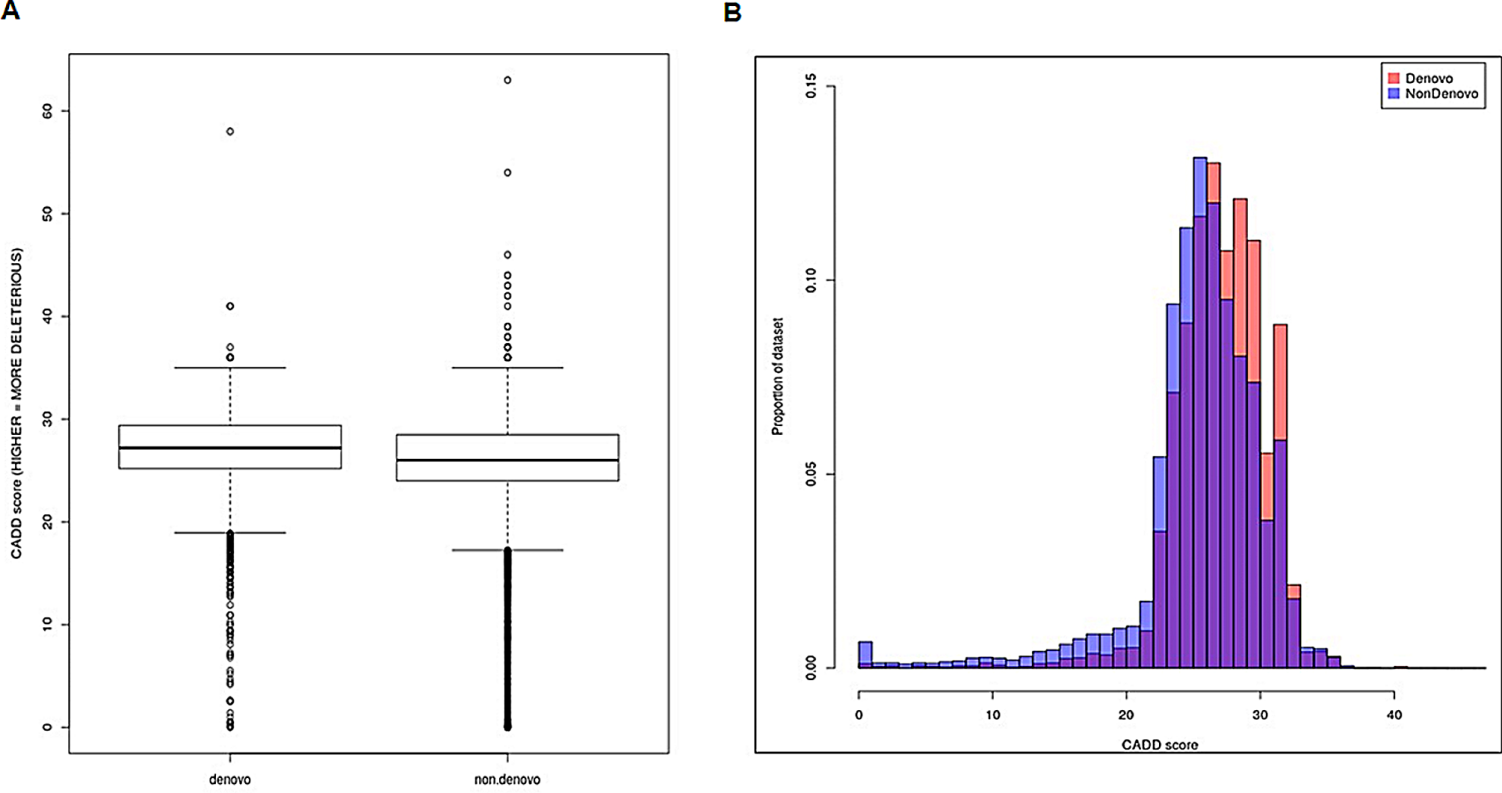
Comparison of predicted deleteriousness of two groups of HGMD disease-causing missense mutations (5,307 DNM versus 32,605 non-DNM) (**A**). A purple bar indicates the proportion of dataset overlap between DNM and non-DNM for a specific range of CADD scores. The colour of the remaining stacked bar indicates an enrichment of a specific dataset at a specific CADD score; thus, red indicates enrichment of the DNM set whereas blue denotes enrichment of the non-DNM set (**B**)

Because disease-associated DNMs are genetic changes that occur in the children of apparently healthy parents, they have not previously experienced negative selection, or at least only during the developmental time window from gametogenesis to adulthood in one generation. As a result, we speculate that DNMs might exert more detrimental effects than disease-associated mutations not reported to be *de novo* which are likely to have been exposed to negative selection for multiple/many generations since their inception [3, 69]. To investigate this postulate further, we first used the extensive collection of DNMs and disease-associated missense mutations available through HGMD not reported to be *de novo*, although we are aware that we cannot exclude the possibility that some non-DNMs have also occurred *de novo*, to ascertain the deleteriousness as measured by CADD scores. In line with our expectation, the CADD scores were found to be significantly higher for missense DNMs than for missense non-DNMs (t-test; *P* < 2.2e^− 16^) (Fig. 4A and B). To further validate these findings, we also calculated the REVEL scores given their high performance with rare variants [26] (Additional file 4). As was observed for CADD scores, there was a statistically significant difference between the two sets (t-test; *P* = 0.0374), indicating that the DNMs set is enriched in missense mutations with greater impact on their protein products. This is consistent with the view that disease-associated variants not reported to be *de novo* have undergone multiple generations of negative selection thereby ensuring that those mutations with the greatest deleterious impact will have been lost from the population and hence would be less likely to contribute to future generations.

## Discussion

Genome and exome sequencing efforts have revealed a high number of DNMs in genes related to human heritable disease. Germline disease-associated DNMs occur in parental germ cells and can be inherited by the offspring leading to a spectrum of health issues ranging from rare Mendelian diseases to complex traits. By using a large dataset of 46,489 DNMs reported in the literature and collected by HGMD, we observed that the most common disease category associated with DNMs is ‘developmental disorder’, possibly a consequence of efforts to sequence large cohorts of patients with these prevalent disorders. Neurodevelopmental disorders are associated with impairments of brain function [70–73] including intellectual disability, autism spectrum disorder, attention-deficit hyperactivity disorder, etc., Although recognized as discrete entities, they represent an interconnected genetic system [74], sharing etiological and genetic risk variants [64–66] that impair the functional integrity of brain-expressed genes related to molecular pathways such as protein synthesis, chromatin remodeling, transcriptional or epigenetic regulation and synaptic signaling [71, 75]. Disease-associated DNMs are intrinsically linked to developmental disorders [3, 17, 27–35], contributing to an estimated prevalence of 400,000 affected children born each year [27]. The most highly represented entity in the disease-associated DNM dataset analysed here was clearly autism. Although definitive evidence is lacking to confirm that locus heterogeneity is significantly higher for autism compared to other neurodevelopmental disorders, a plausible explanation could be the high prevalence of ASD in the general population and the efforts undertaken to sequence the exomes/genomes of affected individuals and their relatives. It is, however, important to note that the set of DNMs analyzed in this work include mutations classified as “DM”, which are clearly linked to the corresponding phenotype as inferred by the original publication, as well as mutations classified as “DM?”. However, these “DM?” variants represent an important source of information because this category of lesion poses a challenge for the interpretation of pathogenicity, which is important for distinguishing the genes that are causal from those that are coincidental. Interpreting the impact of these DNMs can be even more challenging when they occur in genes that have not previously been implicated in any disease [76]. An interesting example was recently reported by Jia and collaborators in the *UBAP2L*, a gene that is involved in regulating stress granule formation during cortical development [77]. This neurodevelopmental disorder involves speech-language impairment, intellectual disability and behavioral problems.

With an average germline *de novo* mutation rate of 1.20 × 10^− 8^ [78] (see also [79–81]), it is expected that an individual’s coding sequence will contain 1–2 DNMs [3, 82]. This low rate of spontaneous occurrence of novel mutations in an individual can be leveraged as a source of information in support of both gene and variant disease candidacy [83]. Whilst many DNMs are still waiting for the confirmation of causal genotype-phenotype linkage, the recurrence of DNMs in different cohorts, plus their absence from control datasets, provides good evidence for pathological authenticity.

A variety of strategies can be employed for the effective evaluation of the impact of individual DNMs prior to functional in vitro testing or analysis in cellular and animal models. For example, scanning protein sequence conservation scores is an important source of information, as it is widely accepted that proteins associated with human disease have been preferentially conserved through evolution [84–87]. In line with this notion, our analysis has shown that amino acid residues affected by DNMs tend to be associated with higher CADD and REVEL scores (Fig. 4 and Additional file 4). In principle, DNMs might also be screened using protein molecular modelling tools and virtual screening [88] and the evaluation of each variant could be performed by free energy binding calculations and chemical descriptors [89]. Additional information might be obtained from other metrics such as the gene damage index [90]. This type of workflow is now possible and could be applied to a large number of DNMs. Very recently, a novel machine learning tool known as AlphaMissense [91] was introduced. This tool utilizes structural information predicted by AlphaFold2 to infer the pathogenicity of human variants, including DNMs, and could help in ranking these variants. Such screening techniques promise to be particularly important in the case of DNMs because, by their very nature, this type of genetic lesions lacks potentially supporting information provided by co-inheritance of the mutation and the clinical phenotype through multi-generational family pedigrees.

One limitation of our study relates to the fact that we used HGMD as a source of disease-associated DNMs. Although the HGMD data are the best available and most accurate source of deleterious DNMs, they do not allow one to consider recurrent DNMs at mutational hotspots. This could in principle impact the interpretation of our findings, although the extent of the impact is unpredictable. Future studies may add this new layer of information that while challenging in terms of data processing, would justify the effort expended in terms of the robustness of the results obtained.

## Conclusions

DNMs appear anew at every generation and are clinically significant in the context of rare and common diseases alike. As the pace of genome sequencing increases, we anticipate a steady increase in the number of DNMs reported, and with it our understanding of the potential contribution of each newly arisen DNM to heritable disease, which is of the utmost importance to the medical genetics field. To the best of our knowledge, the meta-analyses we present here are the largest ever performed on disease-associated DNMs, and we expect that they can represent a gateway for further our understanding of this important category of gene lesions.

### Electronic supplementary material

Below is the link to the electronic supplementary material.

Supplementary Material 1

Supplementary Material 2

Supplementary Material 3

Supplementary Material 4

## Data Availability

All data analysed in this study are included in this published article and its supplementary information files.

## Abbreviations

DNM: *De novo* mutation
HGMD: Human Gene Mutation Database
DM: Disease-causing mutation
DM?: Probable/possible pathogenic mutation
UMLS: Unified Medical Language System
CADD: Combined Annotation Dependent Depletion
GO: Gene Ontology
ASD: Autism Spectrum Disorder
REVEL: Rare Exome Variant Ensemble Learner
