# Supplementary material for: Meta-analysis of 46,000 germline de novo mutations linked to human inherited disease
Source: Hum Genomics. 2024 Feb 23;18:20. doi: 10.1186/s40246-024-00587-8 (PMC10885371; doi:10.1186/s40246-024-00587-8)

Additional file 1. Visual representation of the methodology and analyses performed in this study.

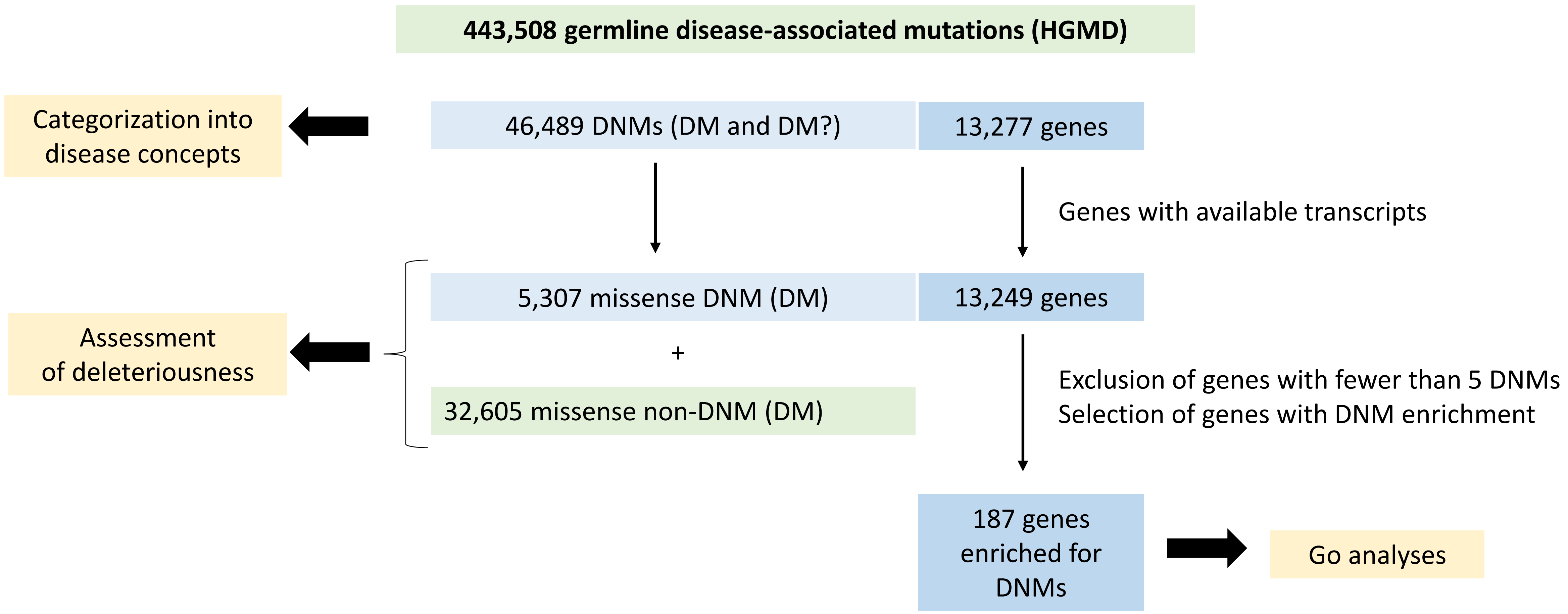

Supplement: Supplementary file 1 — Supplementary Material 1 [file 40246_2024_587_MOESM1_ESM.pdf]
