# Supplementary material for: Meta-analysis of 46,000 germline de novo mutations linked to human inherited disease
Source: Hum Genomics. 2024 Feb 23;18:20. doi: 10.1186/s40246-024-00587-8 (PMC10885371; doi:10.1186/s40246-024-00587-8)
Supplement: Supplementary file 2 — Supplementary Material 2 [file 40246_2024_587_MOESM2_ESM.pdf]

Additional file 3. 190 enriched biological processes identified based on the analysis of 187 DNM enriched disease genes (Additional file 1) using Gene Ontology (GO) analysis.

Annotation Cluster 1	Enrichment Score: 24.538949456603312
Category	Term
GOTERM_BP_ALL	GO:0048731~system development
GOTERM_BP_ALL	GO:0007275~multicellular organism development
GOTERM_BP_ALL	GO:0007399~nervous system development
GOTERM_BP_ALL	GO:0044707~single-multicellular organism process
GOTERM_BP_ALL	GO:0048856~anatomical structure development
GOTERM_BP_ALL	GO:0044767~single-organism developmental process
GOTERM_BP_ALL	GO:0032502~developmental process
GOTERM_BP_ALL	GO:0030154~cell differentiation
GOTERM_BP_ALL	GO:0032501~multicellular organismal process
GOTERM_BP_ALL	GO:0048869~cellular developmental process
GOTERM_BP_ALL	GO:0048513~animal organ development
GOTERM_BP_ALL	GO:0048699~generation of neurons
GOTERM_BP_ALL	GO:0048468~cell development
GOTERM_BP_ALL	GO:0022008~neurogenesis
Annotation Cluster 2	Enrichment Score: 18.06481141083646
Category	Term
GOTERM_BP_ALL	GO:0045893~positive regulation of transcription, DNA-templated
GOTERM_BP_ALL	GO:1903508~positive regulation of nucleic acid-templated transcription
GOTERM_BP_ALL	GO:1902680~positive regulation of RNA biosynthetic process
GOTERM_BP_ALL	GO:0010628~positive regulation of gene expression
GOTERM_BP_ALL	GO:0045935~positive regulation of nucleobase-containing compound metabolic process
GOTERM_BP_ALL	GO:0009893~positive regulation of metabolic process
GOTERM_BP_ALL	GO:0051254~positive regulation of RNA metabolic process
GOTERM_BP_ALL	GO:0009891~positive regulation of biosynthetic process
GOTERM_BP_ALL	GO:0010557~positive regulation of macromolecule biosynthetic process
GOTERM_BP_ALL	GO:0051173~positive regulation of nitrogen compound metabolic process
GOTERM_BP_ALL	GO:0031328~positive regulation of cellular biosynthetic process
GOTERM_BP_ALL	GO:0010604~positive regulation of macromolecule metabolic process
GOTERM_BP_ALL	GO:0048522~positive regulation of cellular process
GOTERM_BP_ALL	GO:0031325~positive regulation of cellular metabolic process
GOTERM_BP_ALL	GO:0097659~nucleic acid-templated transcription
GOTERM_BP_ALL	GO:0051253~negative regulation of RNA metabolic process
GOTERM_BP_ALL	GO:0045944~positive regulation of transcription from RNA polymerase II promoter
GOTERM_BP_ALL	GO:0032774~RNA biosynthetic process
GOTERM_BP_ALL	GO:1903507~negative regulation of nucleic acid-templated transcription
GOTERM_BP_ALL	GO:0006351~transcription, DNA-templated
GOTERM_BP_ALL	GO:1902679~negative regulation of RNA biosynthetic process
GOTERM_BP_ALL	GO:0006366~transcription from RNA polymerase II promoter
GOTERM_BP_ALL	GO:0019219~regulation of nucleobase-containing compound metabolic process
GOTERM_BP_ALL	GO:0051252~regulation of RNA metabolic process
GOTERM_BP_ALL	GO:1903506~regulation of nucleic acid-templated transcription
GOTERM_BP_ALL	GO:2001141~regulation of RNA biosynthetic process

GOTERM_BP_ALL	GO:0045934~negative regulation of nucleobase-containing compound metabolic process
GOTERM_BP_ALL	GO:0048518~positive regulation of biological process
GOTERM_BP_ALL	GO:0045892~negative regulation of transcription, DNA-templated
GOTERM_BP_ALL	GO:0006355~regulation of transcription, DNA-templated
GOTERM_BP_ALL	GO:0006357~regulation of transcription from RNA polymerase II promoter
GOTERM_BP_ALL	GO:0034654~nucleobase-containing compound biosynthetic process
GOTERM_BP_ALL	GO:0018130~heterocycle biosynthetic process
GOTERM_BP_ALL	GO:0019438~aromatic compound biosynthetic process
GOTERM_BP_ALL	GO:0000122~negative regulation of transcription from RNA polymerase II promoter
GOTERM_BP_ALL	GO:0010468~regulation of gene expression
GOTERM_BP_ALL	GO:0051171~regulation of nitrogen compound metabolic process
GOTERM_BP_ALL	GO:1901362~organic cyclic compound biosynthetic process
GOTERM_BP_ALL	GO:0060255~regulation of macromolecule metabolic process
GOTERM_BP_ALL	GO:0019222~regulation of metabolic process
GOTERM_BP_ALL	GO:0048523~negative regulation of cellular process
GOTERM_BP_ALL	GO:0031326~regulation of cellular biosynthetic process
GOTERM_BP_ALL	GO:2000112~regulation of cellular macromolecule biosynthetic process
GOTERM_BP_ALL	GO:0031323~regulation of cellular metabolic process
GOTERM_BP_ALL	GO:0009889~regulation of biosynthetic process
GOTERM_BP_ALL	GO:0010556~regulation of macromolecule biosynthetic process
GOTERM_BP_ALL	GO:0080090~regulation of primary metabolic process
GOTERM_BP_ALL	GO:0016070~RNA metabolic process
GOTERM_BP_ALL	GO:0031324~negative regulation of cellular metabolic process
GOTERM_BP_ALL	GO:0009890~negative regulation of biosynthetic process
GOTERM_BP_ALL	GO:0044271~cellular nitrogen compound biosynthetic process
GOTERM_BP_ALL	GO:0048519~negative regulation of biological process
GOTERM_BP_ALL	GO:0031327~negative regulation of cellular biosynthetic process
GOTERM_BP_ALL	GO:0051172~negative regulation of nitrogen compound metabolic process
GOTERM_BP_ALL	GO:0010605~negative regulation of macromolecule metabolic process
GOTERM_BP_ALL	GO:0010558~negative regulation of macromolecule biosynthetic process
GOTERM_BP_ALL	GO:0090304~nucleic acid metabolic process
GOTERM_BP_ALL	GO:0034645~cellular macromolecule biosynthetic process
GOTERM_BP_ALL	GO:2000113~negative regulation of cellular macromolecule biosynthetic process
GOTERM_BP_ALL	GO:0010629~negative regulation of gene expression
GOTERM_BP_ALL	GO:0009892~negative regulation of metabolic process
GOTERM_BP_ALL	GO:0009059~macromolecule biosynthetic process
GOTERM_BP_ALL	GO:0010467~gene expression
GOTERM_BP_ALL	GO:0006139~nucleobase-containing compound metabolic process
GOTERM_BP_ALL	GO:0044249~cellular biosynthetic process
GOTERM_BP_ALL	GO:0046483~heterocycle metabolic process
GOTERM_BP_ALL	GO:0006725~cellular aromatic compound metabolic process
GOTERM_BP_ALL	GO:0009058~biosynthetic process
GOTERM_BP_ALL	GO:1901576~organic substance biosynthetic process
GOTERM_BP_ALL	GO:1901360~organic cyclic compound metabolic process
GOTERM_BP_ALL	GO:0044260~cellular macromolecule metabolic process
GOTERM_BP_ALL	GO:0043170~macromolecule metabolic process
GOTERM_BP_ALL	GO:0034641~cellular nitrogen compound metabolic process
GOTERM_BP_ALL	GO:0006807~nitrogen compound metabolic process

GOTERM_BP_ALL	GO:0071704~organic substance metabolic process
GOTERM_BP_ALL	GO:0008152~metabolic process
GOTERM_BP_ALL	GO:0044238~primary metabolic process
GOTERM_BP_ALL	GO:0044237~cellular metabolic process
Annotation Cluster 3	Enrichment Score: 16.402881568405938
Category	Term
GOTERM_BP_ALL	GO:0016043~cellular component organization
GOTERM_BP_ALL	GO:0071840~cellular component organization or biogenesis
GOTERM_BP_ALL	GO:0006996~organelle organization
Annotation Cluster 4	Enrichment Score: 12.887988837315419
Category	Term
GOTERM_BP_ALL	GO:0007267~cell-cell signaling
GOTERM_BP_ALL	GO:0007268~chemical synaptic transmission
GOTERM_BP_ALL	GO:0098916~anterograde trans-synaptic signaling
GOTERM_BP_ALL	GO:0050804~modulation of synaptic transmission
GOTERM_BP_ALL	GO:0099537~trans-synaptic signaling
GOTERM_BP_ALL	GO:0099536~synaptic signaling
GOTERM_BP_ALL	GO:0048167~regulation of synaptic plasticity
Annotation Cluster 5	Enrichment Score: 12.791367449280083
Category	Term
GOTERM_BP_ALL	GO:0042221~response to chemical
GOTERM_BP_ALL	GO:0010033~response to organic substance
GOTERM_BP_ALL	GO:0070887~cellular response to chemical stimulus
GOTERM_BP_ALL	GO:0071310~cellular response to organic substance
GOTERM_BP_ALL	GO:0009719~response to endogenous stimulus
GOTERM_BP_ALL	GO:0051716~cellular response to stimulus
GOTERM_BP_ALL	GO:0071495~cellular response to endogenous stimulus
Annotation Cluster 6	Enrichment Score: 12.745786493923193
Category	Term
GOTERM_BP_ALL	GO:0060322~head development
GOTERM_BP_ALL	GO:0007417~central nervous system development
GOTERM_BP_ALL	GO:0007420~brain development
GOTERM_BP_ALL	GO:0030900~forebrain development
GOTERM_BP_ALL	GO:0021537~telencephalon development
GOTERM_BP_ALL	GO:0021987~cerebral cortex development
GOTERM_BP_ALL	GO:0021543~pallium development
Annotation Cluster 7	Enrichment Score: 12.032486964535344
Category	Term
GOTERM_BP_ALL	GO:0030154~cell differentiation
GOTERM_BP_ALL	GO:0048869~cellular developmental process
GOTERM_BP_ALL	GO:0048699~generation of neurons
GOTERM_BP_ALL	GO:0048468~cell development
GOTERM_BP_ALL	GO:2000026~regulation of multicellular organismal development
GOTERM_BP_ALL	GO:0022008~neurogenesis
GOTERM_BP_ALL	GO:0045595~regulation of cell differentiation
GOTERM_BP_ALL	GO:0050793~regulation of developmental process

GOTERM_BP_ALL	GO:0060284~regulation of cell development
GOTERM_BP_ALL	GO:0009653~anatomical structure morphogenesis
GOTERM_BP_ALL	GO:0051239~regulation of multicellular organismal process
GOTERM_BP_ALL	GO:0030182~neuron differentiation
GOTERM_BP_ALL	GO:0045596~negative regulation of cell differentiation
GOTERM_BP_ALL	GO:0048666~neuron development
GOTERM_BP_ALL	GO:0050767~regulation of neurogenesis
GOTERM_BP_ALL	GO:0051960~regulation of nervous system development
GOTERM_BP_ALL	GO:0051093~negative regulation of developmental process
GOTERM_BP_ALL	GO:0051094~positive regulation of developmental process
GOTERM_BP_ALL	GO:0031175~neuron projection development
GOTERM_BP_ALL	GO:0051241~negative regulation of multicellular organismal process
GOTERM_BP_ALL	GO:0032990~cell part morphogenesis
GOTERM_BP_ALL	GO:0048667~cell morphogenesis involved in neuron differentiation
GOTERM_BP_ALL	GO:0045597~positive regulation of cell differentiation
GOTERM_BP_ALL	GO:0048812~neuron projection morphogenesis
GOTERM_BP_ALL	GO:0048858~cell projection morphogenesis
GOTERM_BP_ALL	GO:0000904~cell morphogenesis involved in differentiation
GOTERM_BP_ALL	GO:0030030~cell projection organization
GOTERM_BP_ALL	GO:0045664~regulation of neuron differentiation
GOTERM_BP_ALL	GO:0032989~cellular component morphogenesis
GOTERM_BP_ALL	GO:0007409~axonogenesis
GOTERM_BP_ALL	GO:0061564~axon development
GOTERM_BP_ALL	GO:0000902~cell morphogenesis
GOTERM_BP_ALL	GO:0051240~positive regulation of multicellular organismal process
GOTERM_BP_ALL	GO:0010720~positive regulation of cell development
GOTERM_BP_ALL	GO:0022603~regulation of anatomical structure morphogenesis
GOTERM_BP_ALL	GO:0050769~positive regulation of neurogenesis
GOTERM_BP_ALL	GO:0051962~positive regulation of nervous system development
GOTERM_BP_ALL	GO:0010769~regulation of cell morphogenesis involved in differentiation
GOTERM_BP_ALL	GO:0031344~regulation of cell projection organization
GOTERM_BP_ALL	GO:0010975~regulation of neuron projection development
GOTERM_BP_ALL	GO:0045666~positive regulation of neuron differentiation
GOTERM_BP_ALL	GO:0022604~regulation of cell morphogenesis
Annotation Cluster 8	Enrichment Score: 11.883854154347821
Category	Term
GOTERM_BP_ALL	GO:0008219~cell death
GOTERM_BP_ALL	GO:0006915~apoptotic process
GOTERM_BP_ALL	GO:0012501~programmed cell death
GOTERM_BP_ALL	GO:0010941~regulation of cell death
GOTERM_BP_ALL	GO:0042981~regulation of apoptotic process
GOTERM_BP_ALL	GO:0043067~regulation of programmed cell death
GOTERM_BP_ALL	GO:0043066~negative regulation of apoptotic process
GOTERM_BP_ALL	GO:0060548~negative regulation of cell death
GOTERM_BP_ALL	GO:0043069~negative regulation of programmed cell death
Annotation Cluster 9	Enrichment Score: 11.617721454578012
Category	Term
GOTERM_BP_ALL	GO:0050789~regulation of biological process
GOTERM_BP_ALL	GO:0050794~regulation of cellular process

GOTERM_BP_ALL	GO:0065007~biological regulation
GOTERM_BP_ALL	GO:0009987~cellular process
Annotation Cluster 10	Enrichment Score: 10.877350386983192
Category	Term
GOTERM_BP_ALL	GO:0006325~chromatin organization
GOTERM_BP_ALL	GO:0051276~chromosome organization
GOTERM_BP_ALL	GO:0033044~regulation of chromosome organization
GOTERM_BP_ALL	GO:0006338~chromatin remodeling
GOTERM_BP_ALL	GO:0016569~covalent chromatin modification
GOTERM_BP_ALL	GO:0006259~DNA metabolic process
GOTERM_BP_ALL	GO:0051052~regulation of DNA metabolic process
Annotation Cluster 11	Enrichment Score: 10.777572381307927
Category	Term
GOTERM_BP_ALL	GO:0006996~organelle organization
GOTERM_BP_ALL	GO:0033043~regulation of organelle organization
GOTERM_BP_ALL	GO:1902589~single-organism organelle organization
Annotation Cluster 12	Enrichment Score: 9.870165680795049
Category	Term
GOTERM_BP_ALL	GO:0009887~animal organ morphogenesis
GOTERM_BP_ALL	GO:0009790~embryo development
GOTERM_BP_ALL	GO:0035295~tube development
GOTERM_BP_ALL	GO:0048729~tissue morphogenesis
GOTERM_BP_ALL	GO:0009888~tissue development
GOTERM_BP_ALL	GO:0048598~embryonic morphogenesis
GOTERM_BP_ALL	GO:0035239~tube morphogenesis
GOTERM_BP_ALL	GO:0002009~morphogenesis of an epithelium
GOTERM_BP_ALL	GO:0060429~epithelium development
GOTERM_BP_ALL	GO:0043009~chordate embryonic development
GOTERM_BP_ALL	GO:0009792~embryo development ending in birth or egg hatching
GOTERM_BP_ALL	GO:0048646~anatomical structure formation involved in morphogenesis
GOTERM_BP_ALL	GO:0048568~embryonic organ development
GOTERM_BP_ALL	GO:0060562~epithelial tube morphogenesis
GOTERM_BP_ALL	GO:0001655~urogenital system development
GOTERM_BP_ALL	GO:0072001~renal system development
GOTERM_BP_ALL	GO:0001701~in utero embryonic development
GOTERM_BP_ALL	GO:0030855~epithelial cell differentiation
Annotation Cluster 13	Enrichment Score: 9.65995643564602
Category	Term
GOTERM_BP_ALL	GO:0051128~regulation of cellular component organization
GOTERM_BP_ALL	GO:0033043~regulation of organelle organization
GOTERM_BP_ALL	GO:0051130~positive regulation of cellular component organization
GOTERM_BP_ALL	GO:0010638~positive regulation of organelle organization
Annotation Cluster 14	Enrichment Score: 8.563799367853306
Category	Term
GOTERM_BP_ALL	GO:0010646~regulation of cell communication
GOTERM_BP_ALL	GO:0023051~regulation of signaling

GOTERM_BP_ALL	GO:0007154~cell communication
GOTERM_BP_ALL	GO:0023052~signaling
GOTERM_BP_ALL	GO:0044700~single organism signaling
GOTERM_BP_ALL	GO:0051716~cellular response to stimulus
GOTERM_BP_ALL	GO:0010647~positive regulation of cell communication
GOTERM_BP_ALL	GO:0023056~positive regulation of signaling
GOTERM_BP_ALL	GO:0031399~regulation of protein modification process
GOTERM_BP_ALL	GO:0048583~regulation of response to stimulus
GOTERM_BP_ALL	GO:0009966~regulation of signal transduction
GOTERM_BP_ALL	GO:0050896~response to stimulus
GOTERM_BP_ALL	GO:0048584~positive regulation of response to stimulus
GOTERM_BP_ALL	GO:0035556~intracellular signal transduction
GOTERM_BP_ALL	GO:0009967~positive regulation of signal transduction
GOTERM_BP_ALL	GO:0007165~signal transduction
GOTERM_BP_ALL	GO:0032268~regulation of cellular protein metabolic process
GOTERM_BP_ALL	GO:0007166~cell surface receptor signaling pathway
GOTERM_BP_ALL	GO:0051246~regulation of protein metabolic process
GOTERM_BP_ALL	GO:0051247~positive regulation of protein metabolic process
GOTERM_BP_ALL	GO:0032270~positive regulation of cellular protein metabolic process
GOTERM_BP_ALL	GO:1902531~regulation of intracellular signal transduction
GOTERM_BP_ALL	GO:0001932~regulation of protein phosphorylation
GOTERM_BP_ALL	GO:1902533~positive regulation of intracellular signal transduction
Annotation Cluster 15	Enrichment Score: 8.522382339024787
Category	Term
GOTERM_BP_ALL	GO:0045596~negative regulation of cell differentiation
GOTERM_BP_ALL	GO:0008283~cell proliferation
GOTERM_BP_ALL	GO:0050673~epithelial cell proliferation
GOTERM_BP_ALL	GO:0042127~regulation of cell proliferation
GOTERM_BP_ALL	GO:0050678~regulation of epithelial cell proliferation
GOTERM_BP_ALL	GO:0008284~positive regulation of cell proliferation
GOTERM_BP_ALL	GO:0008285~negative regulation of cell proliferation
GOTERM_BP_ALL	GO:0050680~negative regulation of epithelial cell proliferation
Annotation Cluster 16	Enrichment Score: 7.180668581687825
Category	Term
GOTERM_BP_ALL	GO:0061061~muscle structure development
GOTERM_BP_ALL	GO:0014706~striated muscle tissue development
GOTERM_BP_ALL	GO:0007517~muscle organ development
GOTERM_BP_ALL	GO:0042692~muscle cell differentiation
GOTERM_BP_ALL	GO:0060537~muscle tissue development
GOTERM_BP_ALL	GO:0007519~skeletal muscle tissue development
GOTERM_BP_ALL	GO:0060538~skeletal muscle organ development
GOTERM_BP_ALL	GO:0007507~heart development
GOTERM_BP_ALL	GO:0072359~circulatory system development
GOTERM_BP_ALL	GO:0051146~striated muscle cell differentiation
Annotation Cluster 17	Enrichment Score: 7.125690185410263
Category	Term
GOTERM_BP_ALL	GO:0051402~neuron apoptotic process
GOTERM_BP_ALL	GO:0070997~neuron death

GOTERM_BP_ALL	GO:0043523~regulation of neuron apoptotic process
GOTERM_BP_ALL	GO:1901214~regulation of neuron death
GOTERM_BP_ALL	GO:0043524~negative regulation of neuron apoptotic process
GOTERM_BP_ALL	GO:1901215~negative regulation of neuron death
Annotation Cluster 18	Enrichment Score: 7.034466528327722
Category	Term
GOTERM_BP_ALL	GO:0065008~regulation of biological quality
GOTERM_BP_ALL	GO:0051179~localization
GOTERM_BP_ALL	GO:0032879~regulation of localization
GOTERM_BP_ALL	GO:0051049~regulation of transport
GOTERM_BP_ALL	GO:0051641~cellular localization
GOTERM_BP_ALL	GO:0044765~single-organism transport
GOTERM_BP_ALL	GO:1902578~single-organism localization
GOTERM_BP_ALL	GO:0006810~transport
GOTERM_BP_ALL	GO:0051234~establishment of localization
GOTERM_BP_ALL	GO:1902580~single-organism cellular localization
GOTERM_BP_ALL	GO:0051649~establishment of localization in cell
GOTERM_BP_ALL	GO:0046907~intracellular transport
Annotation Cluster 19	Enrichment Score: 6.840420905191419
Category	Term
GOTERM_BP_ALL	GO:0050890~cognition
GOTERM_BP_ALL	GO:0007611~learning or memory
GOTERM_BP_ALL	GO:0044708~single-organism behavior
GOTERM_BP_ALL	GO:0007610~behavior
GOTERM_BP_ALL	GO:0008306~associative learning
GOTERM_BP_ALL	GO:0003008~system process
GOTERM_BP_ALL	GO:0007612~learning
GOTERM_BP_ALL	GO:0008542~visual learning
GOTERM_BP_ALL	GO:0007632~visual behavior
GOTERM_BP_ALL	GO:0050877~neurological system process
GOTERM_BP_ALL	GO:0048168~regulation of neuronal synaptic plasticity
GOTERM_BP_ALL	GO:0009628~response to abiotic stimulus
GOTERM_BP_ALL	GO:0009314~response to radiation
GOTERM_BP_ALL	GO:0009416~response to light stimulus
Annotation Cluster 20	Enrichment Score: 6.275479568404395
Category	Term
GOTERM_BP_ALL	GO:0045165~cell fate commitment
GOTERM_BP_ALL	GO:0048568~embryonic organ development
GOTERM_BP_ALL	GO:0007389~pattern specification process
GOTERM_BP_ALL	GO:0003002~regionalization
GOTERM_BP_ALL	GO:0048754~branching morphogenesis of an epithelial tube
GOTERM_BP_ALL	GO:0009952~anterior/posterior pattern specification
Annotation Cluster 21	Enrichment Score: 6.254353113865885
Category	Term
GOTERM_BP_ALL	GO:0009719~response to endogenous stimulus
GOTERM_BP_ALL	GO:0010243~response to organonitrogen compound
GOTERM_BP_ALL	GO:1901700~response to oxygen-containing compound

GOTERM_BP_ALL	GO:1901698~response to nitrogen compound
GOTERM_BP_ALL	GO:0071495~cellular response to endogenous stimulus
GOTERM_BP_ALL	GO:0071417~cellular response to organonitrogen compound
GOTERM_BP_ALL	GO:1901699~cellular response to nitrogen compound
GOTERM_BP_ALL	GO:0009725~response to hormone
GOTERM_BP_ALL	GO:1901701~cellular response to oxygen-containing compound
GOTERM_BP_ALL	GO:0032870~cellular response to hormone stimulus
GOTERM_BP_ALL	GO:1901652~response to peptide
GOTERM_BP_ALL	GO:0043434~response to peptide hormone
GOTERM_BP_ALL	GO:1901653~cellular response to peptide
GOTERM_BP_ALL	GO:0071375~cellular response to peptide hormone stimulus
GOTERM_BP_ALL	GO:0032868~response to insulin
GOTERM_BP_ALL	GO:0032869~cellular response to insulin stimulus
Annotation Cluster 22	Enrichment Score: 5.997404981847557
Category	Term
GOTERM_BP_ALL	GO:0043066~negative regulation of apoptotic process
GOTERM_BP_ALL	GO:0009966~regulation of signal transduction
GOTERM_BP_ALL	GO:0043069~negative regulation of programmed cell death
GOTERM_BP_ALL	GO:0097190~apoptotic signaling pathway
GOTERM_BP_ALL	GO:2001233~regulation of apoptotic signaling pathway
GOTERM_BP_ALL	GO:0010648~negative regulation of cell communication
GOTERM_BP_ALL	GO:0023057~negative regulation of signaling
GOTERM_BP_ALL	GO:0009968~negative regulation of signal transduction
GOTERM_BP_ALL	GO:0048585~negative regulation of response to stimulus
GOTERM_BP_ALL	GO:2001234~negative regulation of apoptotic signaling pathway
Annotation Cluster 23	Enrichment Score: 5.831684249050661
Category	Term
GOTERM_BP_ALL	GO:0042063~gliogenesis
GOTERM_BP_ALL	GO:0014009~glial cell proliferation
GOTERM_BP_ALL	GO:0060251~regulation of glial cell proliferation
GOTERM_BP_ALL	GO:0014013~regulation of gliogenesis
GOTERM_BP_ALL	GO:0060253~negative regulation of glial cell proliferation
GOTERM_BP_ALL	GO:0014014~negative regulation of gliogenesis
GOTERM_BP_ALL	GO:0021782~glial cell development
GOTERM_BP_ALL	GO:0014015~positive regulation of gliogenesis
Annotation Cluster 24	Enrichment Score: 5.729004937957952
Category	Term
GOTERM_BP_ALL	GO:0040007~growth
GOTERM_BP_ALL	GO:0016049~cell growth
GOTERM_BP_ALL	GO:0045927~positive regulation of growth
GOTERM_BP_ALL	GO:0030307~positive regulation of cell growth
GOTERM_BP_ALL	GO:0040008~regulation of growth
GOTERM_BP_ALL	GO:0001558~regulation of cell growth
Annotation Cluster 25	Enrichment Score: 5.527060486081129
Category	Term
GOTERM_BP_ALL	GO:0006473~protein acetylation
GOTERM_BP_ALL	GO:0016569~covalent chromatin modification

GOTERM_BP_ALL	GO:0043543~protein acylation
GOTERM_BP_ALL	GO:0016570~histone modification
GOTERM_BP_ALL	GO:0018205~peptidyl-lysine modification
GOTERM_BP_ALL	GO:0018193~peptidyl-amino acid modification
GOTERM_BP_ALL	GO:0018394~peptidyl-lysine acetylation
GOTERM_BP_ALL	GO:0006475~internal protein amino acid acetylation
GOTERM_BP_ALL	GO:0031056~regulation of histone modification
GOTERM_BP_ALL	GO:1901983~regulation of protein acetylation
GOTERM_BP_ALL	GO:0018393~internal peptidyl-lysine acetylation
GOTERM_BP_ALL	GO:0016573~histone acetylation
GOTERM_BP_ALL	GO:2000756~regulation of peptidyl-lysine acetylation
GOTERM_BP_ALL	GO:0035065~regulation of histone acetylation
GOTERM_BP_ALL	GO:2001251~negative regulation of chromosome organization
GOTERM_BP_ALL	GO:1905268~negative regulation of chromatin organization
GOTERM_BP_ALL	GO:0043967~histone H4 acetylation
GOTERM_BP_ALL	GO:0031057~negative regulation of histone modification
Annotation Cluster 26	Enrichment Score: 5.254027834808341
Category	Term
GOTERM_BP_ALL	GO:1905114~cell surface receptor signaling pathway involved in cell-cell signaling
GOTERM_BP_ALL	GO:0016055~Wnt signaling pathway
GOTERM_BP_ALL	GO:0198738~cell-cell signaling by wnt
GOTERM_BP_ALL	GO:0060070~canonical Wnt signaling pathway
GOTERM_BP_ALL	GO:0030111~regulation of Wnt signaling pathway
GOTERM_BP_ALL	GO:0030177~positive regulation of Wnt signaling pathway
GOTERM_BP_ALL	GO:0060828~regulation of canonical Wnt signaling pathway
GOTERM_BP_ALL	GO:0090263~positive regulation of canonical Wnt signaling pathway
Annotation Cluster 27	Enrichment Score: 5.2492917464875495
Category	Term
GOTERM_BP_ALL	GO:0022607~cellular component assembly
GOTERM_BP_ALL	GO:0044085~cellular component biogenesis
GOTERM_BP_ALL	GO:0043933~macromolecular complex subunit organization
GOTERM_BP_ALL	GO:0065003~macromolecular complex assembly
GOTERM_BP_ALL	GO:0071822~protein complex subunit organization
GOTERM_BP_ALL	GO:0070271~protein complex biogenesis
GOTERM_BP_ALL	GO:0006461~protein complex assembly
GOTERM_BP_ALL	GO:0043254~regulation of protein complex assembly
Annotation Cluster 28	Enrichment Score: 5.243960364631991
Category	Term
GOTERM_BP_ALL	GO:0048663~neuron fate commitment
GOTERM_BP_ALL	GO:0001708~cell fate specification
GOTERM_BP_ALL	GO:0030902~hindbrain development
Annotation Cluster 29	Enrichment Score: 5.100968316465785
Category	Term
GOTERM_BP_ALL	GO:1901698~response to nitrogen compound
GOTERM_BP_ALL	GO:0014070~response to organic cyclic compound
GOTERM_BP_ALL	GO:0071407~cellular response to organic cyclic compound

GOTERM_BP_ALL	GO:0033993~response to lipid
GOTERM_BP_ALL	GO:0071396~cellular response to lipid
Annotation Cluster 30	Enrichment Score: 4.9379867338644425
Category	Term
GOTERM_BP_ALL	GO:0031399~regulation of protein modification process
GOTERM_BP_ALL	GO:0009966~regulation of signal transduction
GOTERM_BP_ALL	GO:0006464~cellular protein modification process
GOTERM_BP_ALL	GO:0036211~protein modification process
GOTERM_BP_ALL	GO:0065009~regulation of molecular function
GOTERM_BP_ALL	GO:0035556~intracellular signal transduction
GOTERM_BP_ALL	GO:0009967~positive regulation of signal transduction
GOTERM_BP_ALL	GO:0043412~macromolecule modification
GOTERM_BP_ALL	GO:0032268~regulation of cellular protein metabolic process
GOTERM_BP_ALL	GO:0051246~regulation of protein metabolic process
GOTERM_BP_ALL	GO:0044093~positive regulation of molecular function
GOTERM_BP_ALL	GO:0051338~regulation of transferase activity
GOTERM_BP_ALL	GO:0051247~positive regulation of protein metabolic process
GOTERM_BP_ALL	GO:0032270~positive regulation of cellular protein metabolic process
GOTERM_BP_ALL	GO:0044267~cellular protein metabolic process
GOTERM_BP_ALL	GO:1902531~regulation of intracellular signal transduction
GOTERM_BP_ALL	GO:0050790~regulation of catalytic activity
GOTERM_BP_ALL	GO:0042325~regulation of phosphorylation
GOTERM_BP_ALL	GO:0031401~positive regulation of protein modification process
GOTERM_BP_ALL	GO:0006468~protein phosphorylation
GOTERM_BP_ALL	GO:0001932~regulation of protein phosphorylation
GOTERM_BP_ALL	GO:0019220~regulation of phosphate metabolic process
GOTERM_BP_ALL	GO:0051174~regulation of phosphorus metabolic process
GOTERM_BP_ALL	GO:0019538~protein metabolic process
GOTERM_BP_ALL	GO:0016310~phosphorylation
GOTERM_BP_ALL	GO:0043549~regulation of kinase activity
GOTERM_BP_ALL	GO:0006796~phosphate-containing compound metabolic process
GOTERM_BP_ALL	GO:0006793~phosphorus metabolic process
GOTERM_BP_ALL	GO:1902533~positive regulation of intracellular signal transduction
GOTERM_BP_ALL	GO:0043085~positive regulation of catalytic activity
GOTERM_BP_ALL	GO:0045859~regulation of protein kinase activity
GOTERM_BP_ALL	GO:0042327~positive regulation of phosphorylation
GOTERM_BP_ALL	GO:0010562~positive regulation of phosphorus metabolic process
GOTERM_BP_ALL	GO:0045937~positive regulation of phosphate metabolic process
GOTERM_BP_ALL	GO:0051347~positive regulation of transferase activity
GOTERM_BP_ALL	GO:0001934~positive regulation of protein phosphorylation
GOTERM_BP_ALL	GO:0000165~MAPK cascade
GOTERM_BP_ALL	GO:0023014~signal transduction by protein phosphorylation
GOTERM_BP_ALL	GO:0043408~regulation of MAPK cascade
GOTERM_BP_ALL	GO:0033674~positive regulation of kinase activity
GOTERM_BP_ALL	GO:0044710~single-organism metabolic process
GOTERM_BP_ALL	GO:0045860~positive regulation of protein kinase activity
GOTERM_BP_ALL	GO:0043410~positive regulation of MAPK cascade
Annotation Cluster 31	Enrichment Score: 4.78213385770065
Category	Term

GOTERM_BP_ALL	GO:0040011~locomotion
GOTERM_BP_ALL	GO:0006928~movement of cell or subcellular component
GOTERM_BP_ALL	GO:0016477~cell migration
GOTERM_BP_ALL	GO:0051674~localization of cell
GOTERM_BP_ALL	GO:0048870~cell motility
GOTERM_BP_ALL	GO:0040012~regulation of locomotion
GOTERM_BP_ALL	GO:0051270~regulation of cellular component movement
GOTERM_BP_ALL	GO:0090130~tissue migration
GOTERM_BP_ALL	GO:0030334~regulation of cell migration
GOTERM_BP_ALL	GO:0001667~ameboidal-type cell migration
GOTERM_BP_ALL	GO:2000145~regulation of cell motility
GOTERM_BP_ALL	GO:0010594~regulation of endothelial cell migration
GOTERM_BP_ALL	GO:0043542~endothelial cell migration
GOTERM_BP_ALL	GO:0010632~regulation of epithelial cell migration
GOTERM_BP_ALL	GO:0010631~epithelial cell migration
GOTERM_BP_ALL	GO:0090132~epithelium migration
GOTERM_BP_ALL	GO:0040017~positive regulation of locomotion
GOTERM_BP_ALL	GO:0051272~positive regulation of cellular component movement
GOTERM_BP_ALL	GO:0043535~regulation of blood vessel endothelial cell migration
GOTERM_BP_ALL	GO:0030335~positive regulation of cell migration
GOTERM_BP_ALL	GO:2000147~positive regulation of cell motility
GOTERM_BP_ALL	GO:2000146~negative regulation of cell motility
GOTERM_BP_ALL	GO:0043534~blood vessel endothelial cell migration
GOTERM_BP_ALL	GO:0010595~positive regulation of endothelial cell migration
GOTERM_BP_ALL	GO:0051271~negative regulation of cellular component movement
GOTERM_BP_ALL	GO:0030336~negative regulation of cell migration
GOTERM_BP_ALL	GO:0040013~negative regulation of locomotion
GOTERM_BP_ALL	GO:0010633~negative regulation of epithelial cell migration
GOTERM_BP_ALL	GO:0010634~positive regulation of epithelial cell migration
GOTERM_BP_ALL	GO:0001936~regulation of endothelial cell proliferation
GOTERM_BP_ALL	GO:0001935~endothelial cell proliferation
GOTERM_BP_ALL	GO:0010596~negative regulation of endothelial cell migration
GOTERM_BP_ALL	GO:0043537~negative regulation of blood vessel endothelial cell migration
Annotation Cluster 32	Enrichment Score: 4.756134068385471
Category	Term
GOTERM_BP_ALL	GO:0060562~epithelial tube morphogenesis
GOTERM_BP_ALL	GO:0035148~tube formation
GOTERM_BP_ALL	GO:0001838~embryonic epithelial tube formation
GOTERM_BP_ALL	GO:0072175~epithelial tube formation
GOTERM_BP_ALL	GO:0016331~morphogenesis of embryonic epithelium
GOTERM_BP_ALL	GO:0021915~neural tube development
GOTERM_BP_ALL	GO:0001841~neural tube formation
GOTERM_BP_ALL	GO:0001843~neural tube closure
GOTERM_BP_ALL	GO:0060606~tube closure
GOTERM_BP_ALL	GO:0014020~primary neural tube formation
Annotation Cluster 33	Enrichment Score: 4.643197539293499
Category	Term
GOTERM_BP_ALL	GO:0007409~axonogenesis
GOTERM_BP_ALL	GO:0061564~axon development

GOTERM_BP_ALL	GO:0006935~chemotaxis
GOTERM_BP_ALL	GO:0042330~taxis
GOTERM_BP_ALL	GO:0007411~axon guidance
GOTERM_BP_ALL	GO:0097485~neuron projection guidance
Annotation Cluster 34	Enrichment Score: 4.536570590762885
Category	Term
GOTERM_BP_ALL	GO:0048646~anatomical structure formation involved in morphogenesis
GOTERM_BP_ALL	GO:0072359~circulatory system development
GOTERM_BP_ALL	GO:0001568~blood vessel development
GOTERM_BP_ALL	GO:0048514~blood vessel morphogenesis
GOTERM_BP_ALL	GO:0072358~cardiovascular system development
GOTERM_BP_ALL	GO:0001944~vasculature development
GOTERM_BP_ALL	GO:0001525~angiogenesis
Annotation Cluster 35	Enrichment Score: 4.531912192479839
Category	Term
GOTERM_BP_ALL	GO:0007423~sensory organ development
GOTERM_BP_ALL	GO:0048562~embryonic organ morphogenesis
GOTERM_BP_ALL	GO:0001654~eye development
GOTERM_BP_ALL	GO:0043010~camera-type eye development
GOTERM_BP_ALL	GO:0090596~sensory organ morphogenesis
GOTERM_BP_ALL	GO:0043583~ear development
GOTERM_BP_ALL	GO:0048839~inner ear development
GOTERM_BP_ALL	GO:0048592~eye morphogenesis
GOTERM_BP_ALL	GO:0090102~cochlea development
GOTERM_BP_ALL	GO:0042471~ear morphogenesis
GOTERM_BP_ALL	GO:0042472~inner ear morphogenesis
Annotation Cluster 36	Enrichment Score: 4.35202300009815
Category	Term
GOTERM_BP_ALL	GO:0060425~lung morphogenesis
GOTERM_BP_ALL	GO:0060541~respiratory system development
GOTERM_BP_ALL	GO:0030324~lung development
GOTERM_BP_ALL	GO:0030323~respiratory tube development
GOTERM_BP_ALL	GO:0060428~lung epithelium development
GOTERM_BP_ALL	GO:0060441~epithelial tube branching involved in lung morphogenesis
GOTERM_BP_ALL	GO:0060479~lung cell differentiation
GOTERM_BP_ALL	GO:0060487~lung epithelial cell differentiation
Annotation Cluster 37	Enrichment Score: 4.229605644271566
Category	Term
GOTERM_BP_ALL	GO:0048663~neuron fate commitment
GOTERM_BP_ALL	GO:0021879~forebrain neuron differentiation
GOTERM_BP_ALL	GO:0021877~forebrain neuron fate commitment
GOTERM_BP_ALL	GO:0021872~forebrain generation of neurons
GOTERM_BP_ALL	GO:0021902~commitment of neuronal cell to specific neuron type in forebrain
GOTERM_BP_ALL	GO:0097154~GABAergic neuron differentiation
Annotation Cluster 38	Enrichment Score: 4.1964777759385345

Category	Term
GOTERM_BP_ALL	GO:0000278~mitotic cell cycle
GOTERM_BP_ALL	GO:0007346~regulation of mitotic cell cycle
GOTERM_BP_ALL	GO:0007049~cell cycle
GOTERM_BP_ALL	GO:0051726~regulation of cell cycle
GOTERM_BP_ALL	GO:0010564~regulation of cell cycle process
GOTERM_BP_ALL	GO:1903047~mitotic cell cycle process
GOTERM_BP_ALL	GO:0022402~cell cycle process
GOTERM_BP_ALL	GO:0007067~mitotic nuclear division
GOTERM_BP_ALL	GO:0051983~regulation of chromosome segregation
GOTERM_BP_ALL	GO:0051052~regulation of DNA metabolic process
GOTERM_BP_ALL	GO:0007059~chromosome segregation
GOTERM_BP_ALL	GO:0044770~cell cycle phase transition
GOTERM_BP_ALL	GO:0048285~organelle fission
GOTERM_BP_ALL	GO:0044843~cell cycle G1/S phase transition
GOTERM_BP_ALL	GO:0000819~sister chromatid segregation
GOTERM_BP_ALL	GO:0033045~regulation of sister chromatid segregation
GOTERM_BP_ALL	GO:0044772~mitotic cell cycle phase transition
GOTERM_BP_ALL	GO:1902806~regulation of cell cycle G1/S phase transition
GOTERM_BP_ALL	GO:0007088~regulation of mitotic nuclear division
GOTERM_BP_ALL	GO:0000280~nuclear division
GOTERM_BP_ALL	GO:1901987~regulation of cell cycle phase transition
GOTERM_BP_ALL	GO:0033554~cellular response to stress
GOTERM_BP_ALL	GO:0080135~regulation of cellular response to stress
GOTERM_BP_ALL	GO:0007062~sister chromatid cohesion
GOTERM_BP_ALL	GO:0006950~response to stress
GOTERM_BP_ALL	GO:0000070~mitotic sister chromatid segregation
GOTERM_BP_ALL	GO:0051783~regulation of nuclear division
GOTERM_BP_ALL	GO:0080134~regulation of response to stress
GOTERM_BP_ALL	GO:2001020~regulation of response to DNA damage stimulus
GOTERM_BP_ALL	GO:0098813~nuclear chromosome segregation
GOTERM_BP_ALL	GO:2000045~regulation of G1/S transition of mitotic cell cycle
GOTERM_BP_ALL	GO:1901990~regulation of mitotic cell cycle phase transition
GOTERM_BP_ALL	GO:0033047~regulation of mitotic sister chromatid segregation
GOTERM_BP_ALL	GO:0007064~mitotic sister chromatid cohesion
GOTERM_BP_ALL	GO:0045739~positive regulation of DNA repair
GOTERM_BP_ALL	GO:0045445~myoblast differentiation
GOTERM_BP_ALL	GO:1905818~regulation of chromosome separation
GOTERM_BP_ALL	GO:0045663~positive regulation of myoblast differentiation
GOTERM_BP_ALL	GO:0006974~cellular response to DNA damage stimulus
GOTERM_BP_ALL	GO:0051054~positive regulation of DNA metabolic process
GOTERM_BP_ALL	GO:0006282~regulation of DNA repair
GOTERM_BP_ALL	GO:2001022~positive regulation of response to DNA damage stimulus
GOTERM_BP_ALL	GO:2000781~positive regulation of double-strand break repair
GOTERM_BP_ALL	GO:0030071~regulation of mitotic metaphase/anaphase transition
GOTERM_BP_ALL	GO:1902099~regulation of metaphase/anaphase transition of cell cycle
GOTERM_BP_ALL	GO:2000779~regulation of double-strand break repair
GOTERM_BP_ALL	GO:2000819~regulation of nucleotide-excision repair
GOTERM_BP_ALL	GO:0010965~regulation of mitotic sister chromatid separation
GOTERM_BP_ALL	GO:0044784~metaphase/anaphase transition of cell cycle
GOTERM_BP_ALL	GO:0006302~double-strand break repair

GOTERM_BP_ALL	GO:0051306~mitotic sister chromatid separation
GOTERM_BP_ALL	GO:2000036~regulation of stem cell population maintenance
GOTERM_BP_ALL	GO:0070316~regulation of G0 to G1 transition
GOTERM_BP_ALL	GO:0045661~regulation of myoblast differentiation
GOTERM_BP_ALL	GO:0006281~DNA repair
GOTERM_BP_ALL	GO:0051304~chromosome separation
GOTERM_BP_ALL	GO:1902459~positive regulation of stem cell population maintenance
GOTERM_BP_ALL	GO:0000018~regulation of DNA recombination
GOTERM_BP_ALL	GO:0045911~positive regulation of DNA recombination
GOTERM_BP_ALL	GO:0006310~DNA recombination
Annotation Cluster 39	Enrichment Score: 4.179754312873051
Category	Term
GOTERM_BP_ALL	GO:0019827~stem cell population maintenance
GOTERM_BP_ALL	GO:0098727~maintenance of cell number
GOTERM_BP_ALL	GO:0001708~cell fate specification
GOTERM_BP_ALL	GO:0035019~somatic stem cell population maintenance
GOTERM_BP_ALL	GO:0060795~cell fate commitment involved in formation of primary germ layer
Annotation Cluster 40	Enrichment Score: 4.069078367512534
Category	Term
GOTERM_BP_ALL	GO:0016569~covalent chromatin modification
GOTERM_BP_ALL	GO:0016570~histone modification
GOTERM_BP_ALL	GO:1902275~regulation of chromatin organization
GOTERM_BP_ALL	GO:0018205~peptidyl-lysine modification
GOTERM_BP_ALL	GO:0018394~peptidyl-lysine acetylation
GOTERM_BP_ALL	GO:0031056~regulation of histone modification
GOTERM_BP_ALL	GO:0016571~histone methylation
GOTERM_BP_ALL	GO:0034968~histone lysine methylation
GOTERM_BP_ALL	GO:0008213~protein alkylation
GOTERM_BP_ALL	GO:0006479~protein methylation
GOTERM_BP_ALL	GO:0018022~peptidyl-lysine methylation
GOTERM_BP_ALL	GO:2001252~positive regulation of chromosome organization
GOTERM_BP_ALL	GO:0018027~peptidyl-lysine dimethylation
GOTERM_BP_ALL	GO:0031060~regulation of histone methylation
GOTERM_BP_ALL	GO:0018023~peptidyl-lysine trimethylation
GOTERM_BP_ALL	GO:0043414~macromolecule methylation
GOTERM_BP_ALL	GO:0031058~positive regulation of histone modification
GOTERM_BP_ALL	GO:1905269~positive regulation of chromatin organization
GOTERM_BP_ALL	GO:0032259~methylation
GOTERM_BP_ALL	GO:0051570~regulation of histone H3-K9 methylation
GOTERM_BP_ALL	GO:2000615~regulation of histone H3-K9 acetylation
GOTERM_BP_ALL	GO:1900112~regulation of histone H3-K9 trimethylation
GOTERM_BP_ALL	GO:0061647~histone H3-K9 modification
GOTERM_BP_ALL	GO:0036124~histone H3-K9 trimethylation
GOTERM_BP_ALL	GO:0031062~positive regulation of histone methylation
GOTERM_BP_ALL	GO:0051568~histone H3-K4 methylation
GOTERM_BP_ALL	GO:0051567~histone H3-K9 methylation
GOTERM_BP_ALL	GO:0051569~regulation of histone H3-K4 methylation

Annotation Cluster 41	Enrichment Score: 3.9173162131481574
Category	Term
GOTERM_BP_ALL	GO:0001894~tissue homeostasis
GOTERM_BP_ALL	GO:0060249~anatomical structure homeostasis
GOTERM_BP_ALL	GO:0048873~homeostasis of number of cells within a tissue
GOTERM_BP_ALL	GO:0048871~multicellular organismal homeostasis
Annotation Cluster 42	Enrichment Score: 3.877708844224279
Category	Term
GOTERM_BP_ALL	GO:0042063~gliogenesis
GOTERM_BP_ALL	GO:0010001~glial cell differentiation
GOTERM_BP_ALL	GO:0014013~regulation of gliogenesis
GOTERM_BP_ALL	GO:0048709~oligodendrocyte differentiation
GOTERM_BP_ALL	GO:0021782~glial cell development
GOTERM_BP_ALL	GO:0014003~oligodendrocyte development
GOTERM_BP_ALL	GO:0042552~myelination
GOTERM_BP_ALL	GO:0007272~ensheathment of neurons
GOTERM_BP_ALL	GO:0008366~axon ensheathment
GOTERM_BP_ALL	GO:0032291~axon ensheathment in central nervous system
GOTERM_BP_ALL	GO:0022010~central nervous system myelination
GOTERM_BP_ALL	GO:0007422~peripheral nervous system development
Annotation Cluster 43	Enrichment Score: 3.7805724567534624
Category	Term
GOTERM_BP_ALL	GO:0010721~negative regulation of cell development
GOTERM_BP_ALL	GO:0050768~negative regulation of neurogenesis
GOTERM_BP_ALL	GO:0051961~negative regulation of nervous system development
GOTERM_BP_ALL	GO:0072091~regulation of stem cell proliferation
GOTERM_BP_ALL	GO:0072089~stem cell proliferation
GOTERM_BP_ALL	GO:0061351~neural precursor cell proliferation
GOTERM_BP_ALL	GO:0014013~regulation of gliogenesis
GOTERM_BP_ALL	GO:0007405~neuroblast proliferation
GOTERM_BP_ALL	GO:1902692~regulation of neuroblast proliferation
GOTERM_BP_ALL	GO:0014014~negative regulation of gliogenesis
GOTERM_BP_ALL	GO:0045665~negative regulation of neuron differentiation
GOTERM_BP_ALL	GO:0048546~digestive tract morphogenesis
GOTERM_BP_ALL	GO:2000648~positive regulation of stem cell proliferation
GOTERM_BP_ALL	GO:2000177~regulation of neural precursor cell proliferation
GOTERM_BP_ALL	GO:0031345~negative regulation of cell projection organization
GOTERM_BP_ALL	GO:0010977~negative regulation of neuron projection development
GOTERM_BP_ALL	GO:2000647~negative regulation of stem cell proliferation
GOTERM_BP_ALL	GO:0002052~positive regulation of neuroblast proliferation
GOTERM_BP_ALL	GO:0007406~negative regulation of neuroblast proliferation
GOTERM_BP_ALL	GO:2000178~negative regulation of neural precursor cell proliferation
GOTERM_BP_ALL	GO:2000179~positive regulation of neural precursor cell proliferation
GOTERM_BP_ALL	GO:0006354~DNA-templated transcription, elongation
GOTERM_BP_ALL	GO:0034243~regulation of transcription elongation from RNA polymerase II promoter
GOTERM_BP_ALL	GO:0006368~transcription elongation from RNA polymerase II promoter
GOTERM_BP_ALL	GO:0032784~regulation of DNA-templated transcription, elongation
GOTERM_BP_ALL	GO:0030178~negative regulation of Wnt signaling pathway

GOTERM_BP_ALL	GO:0090090~negative regulation of canonical Wnt signaling pathway
Annotation Cluster 44	Enrichment Score: 3.689270009164847
Category	Term
GOTERM_BP_ALL	GO:0030534~adult behavior
GOTERM_BP_ALL	GO:0050905~neuromuscular process
GOTERM_BP_ALL	GO:0007626~locomotory behavior
GOTERM_BP_ALL	GO:0008344~adult locomotory behavior
Annotation Cluster 45	Enrichment Score: 3.6094506207871873
Category	Term
GOTERM_BP_ALL	GO:0006333~chromatin assembly or disassembly
GOTERM_BP_ALL	GO:0031497~chromatin assembly
GOTERM_BP_ALL	GO:0071103~DNA conformation change
GOTERM_BP_ALL	GO:0006323~DNA packaging
GOTERM_BP_ALL	GO:0071824~protein-DNA complex subunit organization
GOTERM_BP_ALL	GO:0065004~protein-DNA complex assembly
GOTERM_BP_ALL	GO:0034728~nucleosome organization
GOTERM_BP_ALL	GO:0031507~heterochromatin assembly
GOTERM_BP_ALL	GO:0006336~DNA replication-independent nucleosome assembly
GOTERM_BP_ALL	GO:0070828~heterochromatin organization
GOTERM_BP_ALL	GO:0034724~DNA replication-independent nucleosome organization
GOTERM_BP_ALL	GO:0045652~regulation of megakaryocyte differentiation
GOTERM_BP_ALL	GO:0006334~nucleosome assembly
GOTERM_BP_ALL	GO:0071168~protein localization to chromatin
GOTERM_BP_ALL	GO:0032200~telomere organization
GOTERM_BP_ALL	GO:0071459~protein localization to chromosome, centromeric region
GOTERM_BP_ALL	GO:0061644~protein localization to CENP-A containing chromatin
GOTERM_BP_ALL	GO:0045653~negative regulation of megakaryocyte differentiation
GOTERM_BP_ALL	GO:0030219~megakaryocyte differentiation
GOTERM_BP_ALL	GO:0034502~protein localization to chromosome
GOTERM_BP_ALL	GO:0034723~DNA replication-dependent nucleosome organization
GOTERM_BP_ALL	GO:0006335~DNA replication-dependent nucleosome assembly
Annotation Cluster 46	Enrichment Score: 3.507952642664953
Category	Term
GOTERM_BP_ALL	GO:0021537~telencephalon development
GOTERM_BP_ALL	GO:0021987~cerebral cortex development
GOTERM_BP_ALL	GO:0021543~pallium development
GOTERM_BP_ALL	GO:0021795~cerebral cortex cell migration
GOTERM_BP_ALL	GO:0008347~glial cell migration
GOTERM_BP_ALL	GO:0022029~telencephalon cell migration
GOTERM_BP_ALL	GO:0021799~cerebral cortex radially oriented cell migration
GOTERM_BP_ALL	GO:0021885~forebrain cell migration
GOTERM_BP_ALL	GO:0021801~cerebral cortex radial glia guided migration
GOTERM_BP_ALL	GO:0022030~telencephalon glial cell migration
Annotation Cluster 47	Enrichment Score: 3.313120820314416
Category	Term
GOTERM_BP_ALL	GO:0010942~positive regulation of cell death
GOTERM_BP_ALL	GO:0043065~positive regulation of apoptotic process

GOTERM_BP_ALL	GO:0043068~positive regulation of programmed cell death
GOTERM_BP_ALL	GO:2001235~positive regulation of apoptotic signaling pathway
Annotation Cluster 48	Enrichment Score: 3.3067139656130924
Category	Term
GOTERM_BP_ALL	GO:1903706~regulation of hemopoiesis
GOTERM_BP_ALL	GO:0030097~hemopoiesis
GOTERM_BP_ALL	GO:0002520~immune system development
GOTERM_BP_ALL	GO:0048534~hematopoietic or lymphoid organ development
GOTERM_BP_ALL	GO:0002521~leukocyte differentiation
GOTERM_BP_ALL	GO:0045637~regulation of myeloid cell differentiation
GOTERM_BP_ALL	GO:0002682~regulation of immune system process
GOTERM_BP_ALL	GO:1902105~regulation of leukocyte differentiation
GOTERM_BP_ALL	GO:1903707~negative regulation of hemopoiesis
GOTERM_BP_ALL	GO:0030099~myeloid cell differentiation
GOTERM_BP_ALL	GO:0045638~negative regulation of myeloid cell differentiation
GOTERM_BP_ALL	GO:0045321~leukocyte activation
GOTERM_BP_ALL	GO:0001775~cell activation
GOTERM_BP_ALL	GO:0002573~myeloid leukocyte differentiation
GOTERM_BP_ALL	GO:0002376~immune system process
GOTERM_BP_ALL	GO:0002761~regulation of myeloid leukocyte differentiation
GOTERM_BP_ALL	GO:0046649~lymphocyte activation
GOTERM_BP_ALL	GO:0030316~osteoclast differentiation
GOTERM_BP_ALL	GO:0030155~regulation of cell adhesion
GOTERM_BP_ALL	GO:0002683~negative regulation of immune system process
GOTERM_BP_ALL	GO:0030217~T cell differentiation
GOTERM_BP_ALL	GO:0030098~lymphocyte differentiation
GOTERM_BP_ALL	GO:0002694~regulation of leukocyte activation
GOTERM_BP_ALL	GO:0042110~T cell activation
GOTERM_BP_ALL	GO:1903708~positive regulation of hemopoiesis
GOTERM_BP_ALL	GO:1902107~positive regulation of leukocyte differentiation
GOTERM_BP_ALL	GO:0051147~regulation of muscle cell differentiation
GOTERM_BP_ALL	GO:0002696~positive regulation of leukocyte activation
GOTERM_BP_ALL	GO:0045445~myoblast differentiation
GOTERM_BP_ALL	GO:0045670~regulation of osteoclast differentiation
GOTERM_BP_ALL	GO:1902106~negative regulation of leukocyte differentiation
GOTERM_BP_ALL	GO:0050865~regulation of cell activation
GOTERM_BP_ALL	GO:0045785~positive regulation of cell adhesion
GOTERM_BP_ALL	GO:0045663~positive regulation of myoblast differentiation
GOTERM_BP_ALL	GO:0050867~positive regulation of cell activation
GOTERM_BP_ALL	GO:0098602~single organism cell adhesion
GOTERM_BP_ALL	GO:0051249~regulation of lymphocyte activation
GOTERM_BP_ALL	GO:0002762~negative regulation of myeloid leukocyte differentiation
GOTERM_BP_ALL	GO:0002684~positive regulation of immune system process
GOTERM_BP_ALL	GO:1901985~positive regulation of protein acetylation
GOTERM_BP_ALL	GO:2000819~regulation of nucleotide-excision repair
GOTERM_BP_ALL	GO:0043525~positive regulation of neuron apoptotic process
GOTERM_BP_ALL	GO:0051251~positive regulation of lymphocyte activation
GOTERM_BP_ALL	GO:1901216~positive regulation of neuron death
GOTERM_BP_ALL	GO:0050870~positive regulation of T cell activation
GOTERM_BP_ALL	GO:0045671~negative regulation of osteoclast differentiation

GOTERM_BP_ALL	GO:0050863~regulation of T cell activation
GOTERM_BP_ALL	GO:0022409~positive regulation of cell-cell adhesion
GOTERM_BP_ALL	GO:1903039~positive regulation of leukocyte cell-cell adhesion
GOTERM_BP_ALL	GO:0045582~positive regulation of T cell differentiation
GOTERM_BP_ALL	GO:0016337~single organismal cell-cell adhesion
GOTERM_BP_ALL	GO:0070316~regulation of G0 to G1 transition
GOTERM_BP_ALL	GO:0045661~regulation of myoblast differentiation
GOTERM_BP_ALL	GO:0007155~cell adhesion
GOTERM_BP_ALL	GO:1903037~regulation of leukocyte cell-cell adhesion
GOTERM_BP_ALL	GO:0022610~biological adhesion
GOTERM_BP_ALL	GO:0051149~positive regulation of muscle cell differentiation
GOTERM_BP_ALL	GO:0045580~regulation of T cell differentiation
GOTERM_BP_ALL	GO:0045621~positive regulation of lymphocyte differentiation
GOTERM_BP_ALL	GO:0048771~tissue remodeling
GOTERM_BP_ALL	GO:0022407~regulation of cell-cell adhesion
GOTERM_BP_ALL	GO:0007159~leukocyte cell-cell adhesion
GOTERM_BP_ALL	GO:0045619~regulation of lymphocyte differentiation
GOTERM_BP_ALL	GO:0002320~lymphoid progenitor cell differentiation
GOTERM_BP_ALL	GO:0098609~cell-cell adhesion
GOTERM_BP_ALL	GO:2000758~positive regulation of peptidyl-lysine acetylation
GOTERM_BP_ALL	GO:0046849~bone remodeling
GOTERM_BP_ALL	GO:0007219~Notch signaling pathway
GOTERM_BP_ALL	GO:0045765~regulation of angiogenesis
GOTERM_BP_ALL	GO:1901342~regulation of vasculature development
GOTERM_BP_ALL	GO:0016525~negative regulation of angiogenesis
GOTERM_BP_ALL	GO:2000181~negative regulation of blood vessel morphogenesis
GOTERM_BP_ALL	GO:1901343~negative regulation of vasculature development
Annotation Cluster 49	Enrichment Score: 3.1848147575471826
Category	Term
GOTERM_BP_ALL	GO:0048854~brain morphogenesis
GOTERM_BP_ALL	GO:0021761~limbic system development
GOTERM_BP_ALL	GO:0048853~forebrain morphogenesis
Annotation Cluster 50	Enrichment Score: 3.1778615290350793
Category	Term
GOTERM_BP_ALL	GO:0007346~regulation of mitotic cell cycle
GOTERM_BP_ALL	GO:0045787~positive regulation of cell cycle
GOTERM_BP_ALL	GO:0045931~positive regulation of mitotic cell cycle
GOTERM_BP_ALL	GO:0090068~positive regulation of cell cycle process
GOTERM_BP_ALL	GO:0045840~positive regulation of mitotic nuclear division
GOTERM_BP_ALL	GO:0051785~positive regulation of nuclear division
GOTERM_BP_ALL	GO:1901992~positive regulation of mitotic cell cycle phase transition
GOTERM_BP_ALL	GO:1901989~positive regulation of cell cycle phase transition
GOTERM_BP_ALL	GO:0051984~positive regulation of chromosome segregation
Annotation Cluster 51	Enrichment Score: 3.0619237890716957
Category	Term
GOTERM_BP_ALL	GO:0001655~urogenital system development
GOTERM_BP_ALL	GO:0072001~renal system development
GOTERM_BP_ALL	GO:0001709~cell fate determination

GOTERM_BP_ALL	GO:0061326~renal tubule development
GOTERM_BP_ALL	GO:0061333~renal tubule morphogenesis
GOTERM_BP_ALL	GO:0090185~negative regulation of kidney development
GOTERM_BP_ALL	GO:0001656~metanephros development
GOTERM_BP_ALL	GO:0001708~cell fate specification
GOTERM_BP_ALL	GO:0060993~kidney morphogenesis
GOTERM_BP_ALL	GO:0072080~nephron tubule development
GOTERM_BP_ALL	GO:0001822~kidney development
GOTERM_BP_ALL	GO:0061138~morphogenesis of a branching epithelium
GOTERM_BP_ALL	GO:1905330~regulation of morphogenesis of an epithelium
GOTERM_BP_ALL	GO:0048754~branching morphogenesis of an epithelial tube
GOTERM_BP_ALL	GO:0072006~nephron development
GOTERM_BP_ALL	GO:0001763~morphogenesis of a branching structure
GOTERM_BP_ALL	GO:0072009~nephron epithelium development
GOTERM_BP_ALL	GO:0061005~cell differentiation involved in kidney development
GOTERM_BP_ALL	GO:0090183~regulation of kidney development
GOTERM_BP_ALL	GO:2000027~regulation of organ morphogenesis
GOTERM_BP_ALL	GO:0060675~ureteric bud morphogenesis
GOTERM_BP_ALL	GO:0072171~mesonephric tubule morphogenesis
GOTERM_BP_ALL	GO:0072073~kidney epithelium development
GOTERM_BP_ALL	GO:0072189~ureter development
GOTERM_BP_ALL	GO:0072078~nephron tubule morphogenesis
GOTERM_BP_ALL	GO:0072079~nephron tubule formation
GOTERM_BP_ALL	GO:0072179~nephric duct formation
GOTERM_BP_ALL	GO:0072088~nephron epithelium morphogenesis
GOTERM_BP_ALL	GO:0072028~nephron morphogenesis
GOTERM_BP_ALL	GO:0040019~positive regulation of embryonic development
GOTERM_BP_ALL	GO:0010092~specification of animal organ identity
GOTERM_BP_ALL	GO:0072217~negative regulation of metanephros development
GOTERM_BP_ALL	GO:0001657~ureteric bud development
GOTERM_BP_ALL	GO:0072163~mesonephric epithelium development
GOTERM_BP_ALL	GO:0072164~mesonephric tubule development
GOTERM_BP_ALL	GO:0001658~branching involved in ureteric bud morphogenesis
GOTERM_BP_ALL	GO:0001823~mesonephros development
GOTERM_BP_ALL	GO:0060795~cell fate commitment involved in formation of primary germ layer
GOTERM_BP_ALL	GO:2001054~negative regulation of mesenchymal cell apoptotic process
GOTERM_BP_ALL	GO:0048645~animal organ formation
GOTERM_BP_ALL	GO:0072178~nephric duct morphogenesis
GOTERM_BP_ALL	GO:2001053~regulation of mesenchymal cell apoptotic process
GOTERM_BP_ALL	GO:0072182~regulation of nephron tubule epithelial cell differentiation
GOTERM_BP_ALL	GO:0001704~formation of primary germ layer
GOTERM_BP_ALL	GO:0090184~positive regulation of kidney development
GOTERM_BP_ALL	GO:0097152~mesenchymal cell apoptotic process
GOTERM_BP_ALL	GO:0072176~nephric duct development
GOTERM_BP_ALL	GO:0035850~epithelial cell differentiation involved in kidney development
GOTERM_BP_ALL	GO:0048806~genitalia development
GOTERM_BP_ALL	GO:0048538~thymus development
GOTERM_BP_ALL	GO:2000696~regulation of epithelial cell differentiation involved in kidney development
GOTERM_BP_ALL	GO:0060688~regulation of morphogenesis of a branching structure

GOTERM_BP_ALL	GO:0060231~mesenchymal to epithelial transition
GOTERM_BP_ALL	GO:0072202~cell differentiation involved in metanephros development
GOTERM_BP_ALL	GO:0072215~regulation of metanephros development
GOTERM_BP_ALL	GO:0061217~regulation of mesonephros development
GOTERM_BP_ALL	GO:0071695~anatomical structure maturation
GOTERM_BP_ALL	GO:0033077~T cell differentiation in thymus
GOTERM_BP_ALL	GO:1905332~positive regulation of morphogenesis of an epithelium
GOTERM_BP_ALL	GO:0030856~regulation of epithelial cell differentiation
Annotation Cluster 52	Enrichment Score: 3.0361374109240766
Category	Term
GOTERM_BP_ALL	GO:0048485~sympathetic nervous system development
GOTERM_BP_ALL	GO:0048483~autonomic nervous system development
GOTERM_BP_ALL	GO:0002088~lens development in camera-type eye
Annotation Cluster 53	Enrichment Score: 3.006439976242694
Category	Term
GOTERM_BP_ALL	GO:0060425~lung morphogenesis
GOTERM_BP_ALL	GO:0060428~lung epithelium development
GOTERM_BP_ALL	GO:0035272~exocrine system development
GOTERM_BP_ALL	GO:0022612~gland morphogenesis
GOTERM_BP_ALL	GO:0007435~salivary gland morphogenesis
GOTERM_BP_ALL	GO:0007431~salivary gland development
Annotation Cluster 54	Enrichment Score: 2.995370063455802
Category	Term
GOTERM_BP_ALL	GO:0051101~regulation of DNA binding
GOTERM_BP_ALL	GO:0051098~regulation of binding
GOTERM_BP_ALL	GO:2000677~regulation of transcription regulatory region DNA binding
GOTERM_BP_ALL	GO:2000679~positive regulation of transcription regulatory region DNA binding
GOTERM_BP_ALL	GO:0003179~heart valve morphogenesis
GOTERM_BP_ALL	GO:0043388~positive regulation of DNA binding
GOTERM_BP_ALL	GO:0003170~heart valve development
GOTERM_BP_ALL	GO:0051099~positive regulation of binding
GOTERM_BP_ALL	GO:0003180~aortic valve morphogenesis
GOTERM_BP_ALL	GO:0003176~aortic valve development
GOTERM_BP_ALL	GO:1905314~semi-lunar valve development
Annotation Cluster 55	Enrichment Score: 2.9873967114175177
Category	Term
GOTERM_BP_ALL	GO:0030316~osteoclast differentiation
GOTERM_BP_ALL	GO:0060348~bone development
GOTERM_BP_ALL	GO:0036035~osteoclast development
GOTERM_BP_ALL	GO:0098751~bone cell development
GOTERM_BP_ALL	GO:0061515~myeloid cell development
Annotation Cluster 56	Enrichment Score: 2.9498671760362027
Category	Term
GOTERM_BP_ALL	GO:0050808~synapse organization
GOTERM_BP_ALL	GO:0050803~regulation of synapse structure or activity

GOTERM_BP_ALL	GO:0050807~regulation of synapse organization
GOTERM_BP_ALL	GO:0051963~regulation of synapse assembly
Annotation Cluster 57	Enrichment Score: 2.877781764812515
Category	Term
GOTERM_BP_ALL	GO:0060425~lung morphogenesis
GOTERM_BP_ALL	GO:0031016~pancreas development
GOTERM_BP_ALL	GO:0060428~lung epithelium development
GOTERM_BP_ALL	GO:0048546~digestive tract morphogenesis
GOTERM_BP_ALL	GO:0060441~epithelial tube branching involved in lung morphogenesis
GOTERM_BP_ALL	GO:0010464~regulation of mesenchymal cell proliferation
GOTERM_BP_ALL	GO:0010463~mesenchymal cell proliferation
GOTERM_BP_ALL	GO:0060916~mesenchymal cell proliferation involved in lung development
GOTERM_BP_ALL	GO:0048617~embryonic foregut morphogenesis
GOTERM_BP_ALL	GO:0007440~foregut morphogenesis
GOTERM_BP_ALL	GO:0051145~smooth muscle cell differentiation
GOTERM_BP_ALL	GO:0060438~trachea development
GOTERM_BP_ALL	GO:0002053~positive regulation of mesenchymal cell proliferation
GOTERM_BP_ALL	GO:0001570~vasculogenesis
GOTERM_BP_ALL	GO:0009855~determination of bilateral symmetry
GOTERM_BP_ALL	GO:0009799~specification of symmetry
GOTERM_BP_ALL	GO:0007368~determination of left/right symmetry
Annotation Cluster 58	Enrichment Score: 2.8386997977824553
Category	Term
GOTERM_BP_ALL	GO:0060627~regulation of vesicle-mediated transport
GOTERM_BP_ALL	GO:0006897~endocytosis
GOTERM_BP_ALL	GO:0030100~regulation of endocytosis
Annotation Cluster 59	Enrichment Score: 2.7731720947521685
Category	Term
GOTERM_BP_ALL	GO:0048762~mesenchymal cell differentiation
GOTERM_BP_ALL	GO:0048863~stem cell differentiation
GOTERM_BP_ALL	GO:0035272~exocrine system development
GOTERM_BP_ALL	GO:0014033~neural crest cell differentiation
GOTERM_BP_ALL	GO:0003151~outflow tract morphogenesis
GOTERM_BP_ALL	GO:0014032~neural crest cell development
GOTERM_BP_ALL	GO:0014031~mesenchymal cell development
GOTERM_BP_ALL	GO:0048864~stem cell development
GOTERM_BP_ALL	GO:0001755~neural crest cell migration
GOTERM_BP_ALL	GO:0045667~regulation of osteoblast differentiation
Annotation Cluster 60	Enrichment Score: 2.749016924129903
Category	Term
GOTERM_BP_ALL	GO:0048706~embryonic skeletal system development
GOTERM_BP_ALL	GO:0060173~limb development
GOTERM_BP_ALL	GO:0048736~appendage development
GOTERM_BP_ALL	GO:0035107~appendage morphogenesis
GOTERM_BP_ALL	GO:0035108~limb morphogenesis
GOTERM_BP_ALL	GO:0060174~limb bud formation
GOTERM_BP_ALL	GO:0060021~palate development

GOTERM_BP_ALL	GO:0048641~regulation of skeletal muscle tissue development
GOTERM_BP_ALL	GO:0048705~skeletal system morphogenesis
GOTERM_BP_ALL	GO:0035116~embryonic hindlimb morphogenesis
GOTERM_BP_ALL	GO:0035137~hindlimb morphogenesis
GOTERM_BP_ALL	GO:0030326~embryonic limb morphogenesis
GOTERM_BP_ALL	GO:0035113~embryonic appendage morphogenesis
GOTERM_BP_ALL	GO:0042476~odontogenesis
GOTERM_BP_ALL	GO:0048704~embryonic skeletal system morphogenesis
GOTERM_BP_ALL	GO:0042733~embryonic digit morphogenesis
GOTERM_BP_ALL	GO:0035115~embryonic forelimb morphogenesis
GOTERM_BP_ALL	GO:0035136~forelimb morphogenesis
GOTERM_BP_ALL	GO:0030278~regulation of ossification
Annotation Cluster 61	Enrichment Score: 2.6978573474548995
Category	Term
GOTERM_BP_ALL	GO:0060078~regulation of postsynaptic membrane potential
GOTERM_BP_ALL	GO:0042391~regulation of membrane potential
GOTERM_BP_ALL	GO:0050806~positive regulation of synaptic transmission
GOTERM_BP_ALL	GO:0033555~multicellular organismal response to stress
GOTERM_BP_ALL	GO:0060079~excitatory postsynaptic potential
GOTERM_BP_ALL	GO:0007215~glutamate receptor signaling pathway
GOTERM_BP_ALL	GO:0099565~chemical synaptic transmission, postsynaptic
GOTERM_BP_ALL	GO:0035249~synaptic transmission, glutamatergic
GOTERM_BP_ALL	GO:0051966~regulation of synaptic transmission, glutamatergic
GOTERM_BP_ALL	GO:0035235~ionotropic glutamate receptor signaling pathway
GOTERM_BP_ALL	GO:0060291~long-term synaptic potentiation
GOTERM_BP_ALL	GO:2000463~positive regulation of excitatory postsynaptic potential
GOTERM_BP_ALL	GO:0050773~regulation of dendrite development
GOTERM_BP_ALL	GO:0051968~positive regulation of synaptic transmission, glutamatergic
GOTERM_BP_ALL	GO:0098815~modulation of excitatory postsynaptic potential
GOTERM_BP_ALL	GO:0042596~fear response
GOTERM_BP_ALL	GO:0051703~intraspecies interaction between organisms
GOTERM_BP_ALL	GO:0035176~social behavior
GOTERM_BP_ALL	GO:0051705~multi-organism behavior
GOTERM_BP_ALL	GO:2001257~regulation of cation channel activity
GOTERM_BP_ALL	GO:0032412~regulation of ion transmembrane transporter activity
Annotation Cluster 62	Enrichment Score: 2.6889446512299964
Category	Term
GOTERM_BP_ALL	GO:0090596~sensory organ morphogenesis
GOTERM_BP_ALL	GO:0048048~embryonic eye morphogenesis
GOTERM_BP_ALL	GO:0048592~eye morphogenesis
GOTERM_BP_ALL	GO:0060900~embryonic camera-type eye formation
GOTERM_BP_ALL	GO:0048593~camera-type eye morphogenesis
GOTERM_BP_ALL	GO:0048596~embryonic camera-type eye morphogenesis
GOTERM_BP_ALL	GO:0031076~embryonic camera-type eye development
Annotation Cluster 63	Enrichment Score: 2.553148843800438
Category	Term
GOTERM_BP_ALL	GO:0051338~regulation of transferase activity
GOTERM_BP_ALL	GO:0044092~negative regulation of molecular function

GOTERM_BP_ALL	GO:0031400~negative regulation of protein modification process
GOTERM_BP_ALL	GO:0051348~negative regulation of transferase activity
GOTERM_BP_ALL	GO:0032269~negative regulation of cellular protein metabolic process
GOTERM_BP_ALL	GO:0043086~negative regulation of catalytic activity
GOTERM_BP_ALL	GO:0042326~negative regulation of phosphorylation
GOTERM_BP_ALL	GO:0045936~negative regulation of phosphate metabolic process
GOTERM_BP_ALL	GO:0010563~negative regulation of phosphorus metabolic process
GOTERM_BP_ALL	GO:0051248~negative regulation of protein metabolic process
GOTERM_BP_ALL	GO:0001933~negative regulation of protein phosphorylation
GOTERM_BP_ALL	GO:0033673~negative regulation of kinase activity
GOTERM_BP_ALL	GO:1902532~negative regulation of intracellular signal transduction
GOTERM_BP_ALL	GO:0006469~negative regulation of protein kinase activity
GOTERM_BP_ALL	GO:0071901~negative regulation of protein serine/threonine kinase activity
Annotation Cluster 64	Enrichment Score: 2.5438293475803793
Category	Term
GOTERM_BP_ALL	GO:0060341~regulation of cellular localization
GOTERM_BP_ALL	GO:1902582~single-organism intracellular transport
GOTERM_BP_ALL	GO:1903530~regulation of secretion by cell
GOTERM_BP_ALL	GO:0051640~organelle localization
GOTERM_BP_ALL	GO:0051046~regulation of secretion
GOTERM_BP_ALL	GO:0051656~establishment of organelle localization
GOTERM_BP_ALL	GO:1902580~single-organism cellular localization
GOTERM_BP_ALL	GO:0060627~regulation of vesicle-mediated transport
GOTERM_BP_ALL	GO:0051649~establishment of localization in cell
GOTERM_BP_ALL	GO:0099504~synaptic vesicle cycle
GOTERM_BP_ALL	GO:0051047~positive regulation of secretion
GOTERM_BP_ALL	GO:0032386~regulation of intracellular transport
GOTERM_BP_ALL	GO:0046928~regulation of neurotransmitter secretion
GOTERM_BP_ALL	GO:0097479~synaptic vesicle localization
GOTERM_BP_ALL	GO:0061024~membrane organization
GOTERM_BP_ALL	GO:0045956~positive regulation of calcium ion-dependent exocytosis
GOTERM_BP_ALL	GO:0006836~neurotransmitter transport
GOTERM_BP_ALL	GO:0051588~regulation of neurotransmitter transport
GOTERM_BP_ALL	GO:1903307~positive regulation of regulated secretory pathway
GOTERM_BP_ALL	GO:0017157~regulation of exocytosis
GOTERM_BP_ALL	GO:0046907~intracellular transport
GOTERM_BP_ALL	GO:0051648~vesicle localization
GOTERM_BP_ALL	GO:0001505~regulation of neurotransmitter levels
GOTERM_BP_ALL	GO:1903532~positive regulation of secretion by cell
GOTERM_BP_ALL	GO:0032388~positive regulation of intracellular transport
GOTERM_BP_ALL	GO:0097480~establishment of synaptic vesicle localization
GOTERM_BP_ALL	GO:0048489~synaptic vesicle transport
GOTERM_BP_ALL	GO:0045055~regulated exocytosis
GOTERM_BP_ALL	GO:0099003~vesicle-mediated transport in synapse
GOTERM_BP_ALL	GO:0016192~vesicle-mediated transport
GOTERM_BP_ALL	GO:0006887~exocytosis
GOTERM_BP_ALL	GO:1902803~regulation of synaptic vesicle transport
GOTERM_BP_ALL	GO:0051650~establishment of vesicle localization
GOTERM_BP_ALL	GO:1903305~regulation of regulated secretory pathway
GOTERM_BP_ALL	GO:0031630~regulation of synaptic vesicle fusion to presynaptic membrane

GOTERM_BP_ALL	GO:0051954~positive regulation of amine transport
GOTERM_BP_ALL	GO:0035493~SNARE complex assembly
GOTERM_BP_ALL	GO:0044802~single-organism membrane organization
GOTERM_BP_ALL	GO:0045921~positive regulation of exocytosis
GOTERM_BP_ALL	GO:0099643~signal release from synapse
GOTERM_BP_ALL	GO:0007269~neurotransmitter secretion
GOTERM_BP_ALL	GO:0001956~positive regulation of neurotransmitter secretion
GOTERM_BP_ALL	GO:0017156~calcium ion regulated exocytosis
GOTERM_BP_ALL	GO:0099531~presynaptic process involved in chemical synaptic transmission
GOTERM_BP_ALL	GO:0048488~synaptic vesicle endocytosis
GOTERM_BP_ALL	GO:0017158~regulation of calcium ion-dependent exocytosis
GOTERM_BP_ALL	GO:0051590~positive regulation of neurotransmitter transport
GOTERM_BP_ALL	GO:0016079~synaptic vesicle exocytosis
GOTERM_BP_ALL	GO:0031338~regulation of vesicle fusion
GOTERM_BP_ALL	GO:2000300~regulation of synaptic vesicle exocytosis
GOTERM_BP_ALL	GO:0036465~synaptic vesicle recycling
GOTERM_BP_ALL	GO:0072583~clathrin-dependent endocytosis
GOTERM_BP_ALL	GO:0051952~regulation of amine transport
GOTERM_BP_ALL	GO:0006898~receptor-mediated endocytosis
GOTERM_BP_ALL	GO:0015837~amine transport
GOTERM_BP_ALL	GO:0006906~vesicle fusion
GOTERM_BP_ALL	GO:0090174~organelle membrane fusion
GOTERM_BP_ALL	GO:0016050~vesicle organization
GOTERM_BP_ALL	GO:0061025~membrane fusion
GOTERM_BP_ALL	GO:0048278~vesicle docking
GOTERM_BP_ALL	GO:0022406~membrane docking
GOTERM_BP_ALL	GO:0044801~single-organism membrane fusion
GOTERM_BP_ALL	GO:0048284~organelle fusion
Annotation Cluster 65	Enrichment Score: 2.4934831316653367
Category	Term
GOTERM_BP_ALL	GO:0010769~regulation of cell morphogenesis involved in differentiation
GOTERM_BP_ALL	GO:0097061~dendritic spine organization
GOTERM_BP_ALL	GO:0016358~dendrite development
GOTERM_BP_ALL	GO:0060997~dendritic spine morphogenesis
GOTERM_BP_ALL	GO:0048813~dendrite morphogenesis
GOTERM_BP_ALL	GO:0060996~dendritic spine development
GOTERM_BP_ALL	GO:0048814~regulation of dendrite morphogenesis
GOTERM_BP_ALL	GO:0031345~negative regulation of cell projection organization
GOTERM_BP_ALL	GO:0050773~regulation of dendrite development
GOTERM_BP_ALL	GO:0010977~negative regulation of neuron projection development
GOTERM_BP_ALL	GO:0060998~regulation of dendritic spine development
GOTERM_BP_ALL	GO:0061001~regulation of dendritic spine morphogenesis
GOTERM_BP_ALL	GO:0050774~negative regulation of dendrite morphogenesis
GOTERM_BP_ALL	GO:0008361~regulation of cell size
GOTERM_BP_ALL	GO:2000171~negative regulation of dendrite development
GOTERM_BP_ALL	GO:0010771~negative regulation of cell morphogenesis involved in differentiation
GOTERM_BP_ALL	GO:1900006~positive regulation of dendrite development
GOTERM_BP_ALL	GO:0032535~regulation of cellular component size

Annotation Cluster 66	Enrichment Score: 2.475218736519286
Category	Term
GOTERM_BP_ALL	GO:0055123~digestive system development
GOTERM_BP_ALL	GO:0048546~digestive tract morphogenesis
GOTERM_BP_ALL	GO:0048565~digestive tract development
GOTERM_BP_ALL	GO:0048557~embryonic digestive tract morphogenesis
GOTERM_BP_ALL	GO:0048566~embryonic digestive tract development
GOTERM_BP_ALL	GO:0061053~somite development
GOTERM_BP_ALL	GO:0002695~negative regulation of leukocyte activation
GOTERM_BP_ALL	GO:0050866~negative regulation of cell activation
Annotation Cluster 67	Enrichment Score: 2.4444657408729484
Category	Term
GOTERM_BP_ALL	GO:0051932~synaptic transmission, GABAergic
GOTERM_BP_ALL	GO:0060359~response to ammonium ion
GOTERM_BP_ALL	GO:1904862~inhibitory synapse assembly
GOTERM_BP_ALL	GO:0071420~cellular response to histamine
GOTERM_BP_ALL	GO:0007416~synapse assembly
GOTERM_BP_ALL	GO:0007214~gamma-aminobutyric acid signaling pathway
GOTERM_BP_ALL	GO:1902476~chloride transmembrane transport
GOTERM_BP_ALL	GO:0034776~response to histamine
GOTERM_BP_ALL	GO:0006821~chloride transport
GOTERM_BP_ALL	GO:0098661~inorganic anion transmembrane transport
GOTERM_BP_ALL	GO:0098656~anion transmembrane transport
GOTERM_BP_ALL	GO:0015698~inorganic anion transport
GOTERM_BP_ALL	GO:0071242~cellular response to ammonium ion
GOTERM_BP_ALL	GO:0006820~anion transport
GOTERM_BP_ALL	GO:0007186~G-protein coupled receptor signaling pathway
Annotation Cluster 68	Enrichment Score: 2.4240865896161785
Category	Term
GOTERM_BP_ALL	GO:0031060~regulation of histone methylation
GOTERM_BP_ALL	GO:0043414~macromolecule methylation
GOTERM_BP_ALL	GO:0032259~methylation
GOTERM_BP_ALL	GO:0009791~post-embryonic development
GOTERM_BP_ALL	GO:0044728~DNA methylation or demethylation
GOTERM_BP_ALL	GO:0006304~DNA modification
GOTERM_BP_ALL	GO:0006305~DNA alkylation
GOTERM_BP_ALL	GO:0006306~DNA methylation
Annotation Cluster 69	Enrichment Score: 2.415176653848473
Category	Term
GOTERM_BP_ALL	GO:0044087~regulation of cellular component biogenesis
GOTERM_BP_ALL	GO:0006352~DNA-templated transcription, initiation
GOTERM_BP_ALL	GO:0043254~regulation of protein complex assembly
GOTERM_BP_ALL	GO:2000142~regulation of DNA-templated transcription, initiation
GOTERM_BP_ALL	GO:2000144~positive regulation of DNA-templated transcription, initiation
GOTERM_BP_ALL	GO:0031334~positive regulation of protein complex assembly
GOTERM_BP_ALL	GO:0044089~positive regulation of cellular component biogenesis
Annotation Cluster 70	Enrichment Score: 2.3453724721640676

Category	Term
GOTERM_BP_ALL	GO:1902580~single-organism cellular localization
GOTERM_BP_ALL	GO:0051649~establishment of localization in cell
GOTERM_BP_ALL	GO:0034613~cellular protein localization
GOTERM_BP_ALL	GO:0070727~cellular macromolecule localization
GOTERM_BP_ALL	GO:0008104~protein localization
GOTERM_BP_ALL	GO:0033036~macromolecule localization
GOTERM_BP_ALL	GO:0033365~protein localization to organelle
GOTERM_BP_ALL	GO:0032880~regulation of protein localization
GOTERM_BP_ALL	GO:0046907~intracellular transport
GOTERM_BP_ALL	GO:0071705~nitrogen compound transport
GOTERM_BP_ALL	GO:0090087~regulation of peptide transport
GOTERM_BP_ALL	GO:0070201~regulation of establishment of protein localization
GOTERM_BP_ALL	GO:0042886~amide transport
GOTERM_BP_ALL	GO:0071702~organic substance transport
GOTERM_BP_ALL	GO:0015833~peptide transport
GOTERM_BP_ALL	GO:0051223~regulation of protein transport
GOTERM_BP_ALL	GO:0015031~protein transport
GOTERM_BP_ALL	GO:0006886~intracellular protein transport
GOTERM_BP_ALL	GO:0045184~establishment of protein localization
GOTERM_BP_ALL	GO:0006605~protein targeting
Annotation Cluster 71	Enrichment Score: 2.2821797359816793
Category	Term
GOTERM_BP_ALL	GO:0009743~response to carbohydrate
GOTERM_BP_ALL	GO:0009749~response to glucose
GOTERM_BP_ALL	GO:0009746~response to hexose
GOTERM_BP_ALL	GO:0034284~response to monosaccharide
GOTERM_BP_ALL	GO:0071333~cellular response to glucose stimulus
GOTERM_BP_ALL	GO:0071331~cellular response to hexose stimulus
GOTERM_BP_ALL	GO:0071326~cellular response to monosaccharide stimulus
GOTERM_BP_ALL	GO:0042593~glucose homeostasis
GOTERM_BP_ALL	GO:0033500~carbohydrate homeostasis
GOTERM_BP_ALL	GO:0071322~cellular response to carbohydrate stimulus
GOTERM_BP_ALL	GO:0001678~cellular glucose homeostasis
GOTERM_BP_ALL	GO:0050796~regulation of insulin secretion
GOTERM_BP_ALL	GO:0030073~insulin secretion
Annotation Cluster 72	Enrichment Score: 2.2633599432844442
Category	Term
GOTERM_BP_ALL	GO:0009991~response to extracellular stimulus
GOTERM_BP_ALL	GO:0031668~cellular response to extracellular stimulus
GOTERM_BP_ALL	GO:0031667~response to nutrient levels
GOTERM_BP_ALL	GO:0071496~cellular response to external stimulus
GOTERM_BP_ALL	GO:0031669~cellular response to nutrient levels
Annotation Cluster 73	Enrichment Score: 2.260025927088074
Category	Term
GOTERM_BP_ALL	GO:0048705~skeletal system morphogenesis
GOTERM_BP_ALL	GO:0051216~cartilage development
GOTERM_BP_ALL	GO:0061448~connective tissue development

GOTERM_BP_ALL	GO:0002062~chondrocyte differentiation
Annotation Cluster 74	Enrichment Score: 2.2330620609233516
Category	Term
GOTERM_BP_ALL	GO:0010717~regulation of epithelial to mesenchymal transition
GOTERM_BP_ALL	GO:0001837~epithelial to mesenchymal transition
GOTERM_BP_ALL	GO:0010719~negative regulation of epithelial to mesenchymal transition
GOTERM_BP_ALL	GO:1905331~negative regulation of morphogenesis of an epithelium
GOTERM_BP_ALL	GO:0051896~regulation of protein kinase B signaling
GOTERM_BP_ALL	GO:0010718~positive regulation of epithelial to mesenchymal transition
Annotation Cluster 75	Enrichment Score: 2.232778261874327
Category	Term
GOTERM_BP_ALL	GO:0021782~glial cell development
GOTERM_BP_ALL	GO:0048708~astrocyte differentiation
GOTERM_BP_ALL	GO:0014002~astrocyte development
Annotation Cluster 76	Enrichment Score: 2.2327508400011555
Category	Term
GOTERM_BP_ALL	GO:0001708~cell fate specification
GOTERM_BP_ALL	GO:0007369~gastrulation
GOTERM_BP_ALL	GO:0060795~cell fate commitment involved in formation of primary germ layer
GOTERM_BP_ALL	GO:0001711~endodermal cell fate commitment
GOTERM_BP_ALL	GO:0001704~formation of primary germ layer
GOTERM_BP_ALL	GO:0007492~endoderm development
GOTERM_BP_ALL	GO:0035987~endodermal cell differentiation
GOTERM_BP_ALL	GO:0001706~endoderm formation
Annotation Cluster 77	Enrichment Score: 2.1824225021456827
Category	Term
GOTERM_BP_ALL	GO:0048485~sympathetic nervous system development
GOTERM_BP_ALL	GO:0003208~cardiac ventricle morphogenesis
GOTERM_BP_ALL	GO:0003206~cardiac chamber morphogenesis
GOTERM_BP_ALL	GO:0003211~cardiac ventricle formation
GOTERM_BP_ALL	GO:0003205~cardiac chamber development
GOTERM_BP_ALL	GO:0003207~cardiac chamber formation
GOTERM_BP_ALL	GO:0003231~cardiac ventricle development
GOTERM_BP_ALL	GO:0003215~cardiac right ventricle morphogenesis
GOTERM_BP_ALL	GO:0003279~cardiac septum development
GOTERM_BP_ALL	GO:0060840~artery development
GOTERM_BP_ALL	GO:0060411~cardiac septum morphogenesis
GOTERM_BP_ALL	GO:0048844~artery morphogenesis
GOTERM_BP_ALL	GO:0003281~ventricular septum development
Annotation Cluster 78	Enrichment Score: 2.1812724505392875
Category	Term
GOTERM_BP_ALL	GO:0030318~melanocyte differentiation
GOTERM_BP_ALL	GO:0050931~pigment cell differentiation
GOTERM_BP_ALL	GO:0048066~developmental pigmentation
GOTERM_BP_ALL	GO:0043473~pigmentation

Annotation Cluster 79	Enrichment Score: 2.1676779302514366
Category	Term
GOTERM_BP_ALL	GO:0006476~protein deacetylation
GOTERM_BP_ALL	GO:0035601~protein deacylation
GOTERM_BP_ALL	GO:0098732~macromolecule deacylation
GOTERM_BP_ALL	GO:0016575~histone deacetylation
Annotation Cluster 80	Enrichment Score: 2.151160068980582
Category	Term
GOTERM_BP_ALL	GO:0007163~establishment or maintenance of cell polarity
GOTERM_BP_ALL	GO:0001738~morphogenesis of a polarized epithelium
GOTERM_BP_ALL	GO:0030010~establishment of cell polarity
Annotation Cluster 81	Enrichment Score: 2.128366892415545
Category	Term
GOTERM_BP_ALL	GO:0060359~response to ammonium ion
GOTERM_BP_ALL	GO:0042220~response to cocaine
GOTERM_BP_ALL	GO:0043279~response to alkaloid
Annotation Cluster 82	Enrichment Score: 2.1232867671901228
Category	Term
GOTERM_BP_ALL	GO:0048732~gland development
GOTERM_BP_ALL	GO:0048589~developmental growth
GOTERM_BP_ALL	GO:0007548~sex differentiation
GOTERM_BP_ALL	GO:0048608~reproductive structure development
GOTERM_BP_ALL	GO:0061458~reproductive system development
GOTERM_BP_ALL	GO:0003006~developmental process involved in reproduction
GOTERM_BP_ALL	GO:0022414~reproductive process
GOTERM_BP_ALL	GO:0000003~reproduction
GOTERM_BP_ALL	GO:0044702~single organism reproductive process
GOTERM_BP_ALL	GO:0008406~gonad development
GOTERM_BP_ALL	GO:0045137~development of primary sexual characteristics
GOTERM_BP_ALL	GO:0006997~nucleus organization
GOTERM_BP_ALL	GO:0048609~multicellular organismal reproductive process
GOTERM_BP_ALL	GO:0032504~multicellular organism reproduction
GOTERM_BP_ALL	GO:0019953~sexual reproduction
GOTERM_BP_ALL	GO:0044703~multi-organism reproductive process
GOTERM_BP_ALL	GO:0051704~multi-organism process
GOTERM_BP_ALL	GO:0007281~germ cell development
GOTERM_BP_ALL	GO:0007276~gamete generation
GOTERM_BP_ALL	GO:0022412~cellular process involved in reproduction in multicellular organism
GOTERM_BP_ALL	GO:0007286~spermatid development
GOTERM_BP_ALL	GO:0048515~spermatid differentiation
GOTERM_BP_ALL	GO:0007283~spermatogenesis
GOTERM_BP_ALL	GO:0048232~male gamete generation
Annotation Cluster 83	Enrichment Score: 2.12157421878766
Category	Term
GOTERM_BP_ALL	GO:0060736~prostate gland growth

GOTERM_BP_ALL	GO:0035265~organ growth
GOTERM_BP_ALL	GO:0030850~prostate gland development
Annotation Cluster 84	Enrichment Score: 2.1112154672240595
Category	Term
GOTERM_BP_ALL	GO:0001558~regulation of cell growth
GOTERM_BP_ALL	GO:0045926~negative regulation of growth
GOTERM_BP_ALL	GO:0030308~negative regulation of cell growth
GOTERM_BP_ALL	GO:0006275~regulation of DNA replication
Annotation Cluster 85	Enrichment Score: 2.0765990438777546
Category	Term
GOTERM_BP_ALL	GO:0030901~midbrain development
GOTERM_BP_ALL	GO:0045995~regulation of embryonic development
GOTERM_BP_ALL	GO:0040019~positive regulation of embryonic development
GOTERM_BP_ALL	GO:0071542~dopaminergic neuron differentiation
GOTERM_BP_ALL	GO:0040036~regulation of fibroblast growth factor receptor signaling pathway
GOTERM_BP_ALL	GO:0009948~anterior/posterior axis specification
GOTERM_BP_ALL	GO:0009798~axis specification
Annotation Cluster 86	Enrichment Score: 2.047775244167954
Category	Term
GOTERM_BP_ALL	GO:0032940~secretion by cell
GOTERM_BP_ALL	GO:0046903~secretion
GOTERM_BP_ALL	GO:1903530~regulation of secretion by cell
GOTERM_BP_ALL	GO:0051046~regulation of secretion
GOTERM_BP_ALL	GO:0051050~positive regulation of transport
GOTERM_BP_ALL	GO:0023061~signal release
GOTERM_BP_ALL	GO:0051047~positive regulation of secretion
GOTERM_BP_ALL	GO:1903532~positive regulation of secretion by cell
GOTERM_BP_ALL	GO:0046879~hormone secretion
GOTERM_BP_ALL	GO:0009914~hormone transport
GOTERM_BP_ALL	GO:0042593~glucose homeostasis
GOTERM_BP_ALL	GO:0033500~carbohydrate homeostasis
GOTERM_BP_ALL	GO:0046883~regulation of hormone secretion
GOTERM_BP_ALL	GO:0051051~negative regulation of transport
GOTERM_BP_ALL	GO:0010817~regulation of hormone levels
GOTERM_BP_ALL	GO:0030072~peptide hormone secretion
GOTERM_BP_ALL	GO:0090087~regulation of peptide transport
GOTERM_BP_ALL	GO:0070201~regulation of establishment of protein localization
GOTERM_BP_ALL	GO:0051222~positive regulation of protein transport
GOTERM_BP_ALL	GO:0090276~regulation of peptide hormone secretion
GOTERM_BP_ALL	GO:0051223~regulation of protein transport
GOTERM_BP_ALL	GO:0002790~peptide secretion
GOTERM_BP_ALL	GO:1903531~negative regulation of secretion by cell
GOTERM_BP_ALL	GO:0009306~protein secretion
GOTERM_BP_ALL	GO:0031018~endocrine pancreas development
GOTERM_BP_ALL	GO:0002791~regulation of peptide secretion
GOTERM_BP_ALL	GO:0051048~negative regulation of secretion
GOTERM_BP_ALL	GO:0050708~regulation of protein secretion

GOTERM_BP_ALL	GO:0046887~positive regulation of hormone secretion
GOTERM_BP_ALL	GO:0050709~negative regulation of protein secretion
GOTERM_BP_ALL	GO:0002792~negative regulation of peptide secretion
GOTERM_BP_ALL	GO:0051224~negative regulation of protein transport
GOTERM_BP_ALL	GO:1904950~negative regulation of establishment of protein localization
GOTERM_BP_ALL	GO:0050796~regulation of insulin secretion
GOTERM_BP_ALL	GO:0030073~insulin secretion
GOTERM_BP_ALL	GO:0050714~positive regulation of protein secretion
GOTERM_BP_ALL	GO:0002793~positive regulation of peptide secretion
Annotation Cluster 87	Enrichment Score: 2.024681119758309
Category	Term
GOTERM_BP_ALL	GO:1902893~regulation of pri-miRNA transcription from RNA polymerase II promoter
GOTERM_BP_ALL	GO:2000647~negative regulation of stem cell proliferation
GOTERM_BP_ALL	GO:0061614~pri-miRNA transcription from RNA polymerase II promoter
GOTERM_BP_ALL	GO:1902895~positive regulation of pri-miRNA transcription from RNA polymerase II promoter
Annotation Cluster 88	Enrichment Score: 2.0155112836713607
Category	Term
GOTERM_BP_ALL	GO:0014065~phosphatidylinositol 3-kinase signaling
GOTERM_BP_ALL	GO:0048015~phosphatidylinositol-mediated signaling
GOTERM_BP_ALL	GO:0048017~inositol lipid-mediated signaling
GOTERM_BP_ALL	GO:0043491~protein kinase B signaling
GOTERM_BP_ALL	GO:0014066~regulation of phosphatidylinositol 3-kinase signaling
GOTERM_BP_ALL	GO:0051896~regulation of protein kinase B signaling
GOTERM_BP_ALL	GO:0014067~negative regulation of phosphatidylinositol 3-kinase signaling
GOTERM_BP_ALL	GO:0051898~negative regulation of protein kinase B signaling
GOTERM_BP_ALL	GO:0051897~positive regulation of protein kinase B signaling
GOTERM_BP_ALL	GO:0014068~positive regulation of phosphatidylinositol 3-kinase signaling
Annotation Cluster 89	Enrichment Score: 2.0065904361998568
Category	Term
GOTERM_BP_ALL	GO:0007405~neuroblast proliferation
GOTERM_BP_ALL	GO:1902692~regulation of neuroblast proliferation
GOTERM_BP_ALL	GO:0001656~metanephros development
GOTERM_BP_ALL	GO:0048709~oligodendrocyte differentiation
GOTERM_BP_ALL	GO:2000177~regulation of neural precursor cell proliferation
GOTERM_BP_ALL	GO:1902106~negative regulation of leukocyte differentiation
GOTERM_BP_ALL	GO:0070663~regulation of leukocyte proliferation
GOTERM_BP_ALL	GO:0048713~regulation of oligodendrocyte differentiation
GOTERM_BP_ALL	GO:0045685~regulation of glial cell differentiation
GOTERM_BP_ALL	GO:0043588~skin development
GOTERM_BP_ALL	GO:0001942~hair follicle development
GOTERM_BP_ALL	GO:0022404~molting cycle process
GOTERM_BP_ALL	GO:0022405~hair cycle process
GOTERM_BP_ALL	GO:0098773~skin epidermis development
GOTERM_BP_ALL	GO:0042633~hair cycle
GOTERM_BP_ALL	GO:0042303~molting cycle
GOTERM_BP_ALL	GO:0008544~epidermis development

Annotation Cluster 90	Enrichment Score: 1.9965952406642915
Category	Term
GOTERM_BP_ALL	GO:0021510~spinal cord development
GOTERM_BP_ALL	GO:0021517~ventral spinal cord development
GOTERM_BP_ALL	GO:0021515~cell differentiation in spinal cord
GOTERM_BP_ALL	GO:0021522~spinal cord motor neuron differentiation
Annotation Cluster 91	Enrichment Score: 1.9830428588509526
Category	Term
GOTERM_BP_ALL	GO:0007519~skeletal muscle tissue development
GOTERM_BP_ALL	GO:0001649~osteoblast differentiation
GOTERM_BP_ALL	GO:0060538~skeletal muscle organ development
GOTERM_BP_ALL	GO:0061005~cell differentiation involved in kidney development
GOTERM_BP_ALL	GO:0048641~regulation of skeletal muscle tissue development
GOTERM_BP_ALL	GO:0048643~positive regulation of skeletal muscle tissue development
GOTERM_BP_ALL	GO:0050870~positive regulation of T cell activation
GOTERM_BP_ALL	GO:0022409~positive regulation of cell-cell adhesion
GOTERM_BP_ALL	GO:0048645~animal organ formation
GOTERM_BP_ALL	GO:0051145~smooth muscle cell differentiation
GOTERM_BP_ALL	GO:1903039~positive regulation of leukocyte cell-cell adhesion
GOTERM_BP_ALL	GO:0070661~leukocyte proliferation
GOTERM_BP_ALL	GO:0003151~outflow tract morphogenesis
GOTERM_BP_ALL	GO:0048771~tissue remodeling
GOTERM_BP_ALL	GO:0046651~lymphocyte proliferation
GOTERM_BP_ALL	GO:0101023~vascular endothelial cell proliferation
GOTERM_BP_ALL	GO:0032943~mononuclear cell proliferation
GOTERM_BP_ALL	GO:1905562~regulation of vascular endothelial cell proliferation
GOTERM_BP_ALL	GO:0070663~regulation of leukocyte proliferation
GOTERM_BP_ALL	GO:0050670~regulation of lymphocyte proliferation
GOTERM_BP_ALL	GO:0042104~positive regulation of activated T cell proliferation
GOTERM_BP_ALL	GO:0032944~regulation of mononuclear cell proliferation
GOTERM_BP_ALL	GO:0048636~positive regulation of muscle organ development
GOTERM_BP_ALL	GO:0045844~positive regulation of striated muscle tissue development
GOTERM_BP_ALL	GO:1901863~positive regulation of muscle tissue development
GOTERM_BP_ALL	GO:0045669~positive regulation of osteoblast differentiation
GOTERM_BP_ALL	GO:0009855~determination of bilateral symmetry
GOTERM_BP_ALL	GO:0009799~specification of symmetry
GOTERM_BP_ALL	GO:0035050~embryonic heart tube development
GOTERM_BP_ALL	GO:0050671~positive regulation of lymphocyte proliferation
GOTERM_BP_ALL	GO:0032946~positive regulation of mononuclear cell proliferation
GOTERM_BP_ALL	GO:0045667~regulation of osteoblast differentiation
GOTERM_BP_ALL	GO:0070665~positive regulation of leukocyte proliferation
GOTERM_BP_ALL	GO:0046006~regulation of activated T cell proliferation
GOTERM_BP_ALL	GO:0030278~regulation of ossification
GOTERM_BP_ALL	GO:0050798~activated T cell proliferation
GOTERM_BP_ALL	GO:0042102~positive regulation of T cell proliferation
GOTERM_BP_ALL	GO:0045778~positive regulation of ossification
GOTERM_BP_ALL	GO:0042129~regulation of T cell proliferation
GOTERM_BP_ALL	GO:0014902~myotube differentiation
GOTERM_BP_ALL	GO:0042098~T cell proliferation

GOTERM_BP_ALL	GO:0002695~negative regulation of leukocyte activation
GOTERM_BP_ALL	GO:0050866~negative regulation of cell activation
GOTERM_BP_ALL	GO:0051250~negative regulation of lymphocyte activation
Annotation Cluster 92	Enrichment Score: 1.9755288688611632
Category	Term
GOTERM_BP_ALL	GO:0007265~Ras protein signal transduction
GOTERM_BP_ALL	GO:0007264~small GTPase mediated signal transduction
GOTERM_BP_ALL	GO:0016601~Rac protein signal transduction
GOTERM_BP_ALL	GO:0030029~actin filament-based process
GOTERM_BP_ALL	GO:0046578~regulation of Ras protein signal transduction
GOTERM_BP_ALL	GO:0030036~actin cytoskeleton organization
GOTERM_BP_ALL	GO:0051056~regulation of small GTPase mediated signal transduction
GOTERM_BP_ALL	GO:0046580~negative regulation of Ras protein signal transduction
GOTERM_BP_ALL	GO:0051058~negative regulation of small GTPase mediated signal transduction
Annotation Cluster 93	Enrichment Score: 1.9310325902581853
Category	Term
GOTERM_BP_ALL	GO:0070482~response to oxygen levels
GOTERM_BP_ALL	GO:0001666~response to hypoxia
GOTERM_BP_ALL	GO:0036293~response to decreased oxygen levels
GOTERM_BP_ALL	GO:0007568~aging
GOTERM_BP_ALL	GO:0071453~cellular response to oxygen levels
GOTERM_BP_ALL	GO:0090398~cellular senescence
GOTERM_BP_ALL	GO:0090342~regulation of cell aging
GOTERM_BP_ALL	GO:0071456~cellular response to hypoxia
GOTERM_BP_ALL	GO:0036294~cellular response to decreased oxygen levels
GOTERM_BP_ALL	GO:0007569~cell aging
GOTERM_BP_ALL	GO:2000772~regulation of cellular senescence
GOTERM_BP_ALL	GO:0044282~small molecule catabolic process
Annotation Cluster 94	Enrichment Score: 1.910404336447221
Category	Term
GOTERM_BP_ALL	GO:0042592~homeostatic process
GOTERM_BP_ALL	GO:0042391~regulation of membrane potential
GOTERM_BP_ALL	GO:0043269~regulation of ion transport
GOTERM_BP_ALL	GO:0034762~regulation of transmembrane transport
GOTERM_BP_ALL	GO:0006811~ion transport
GOTERM_BP_ALL	GO:0034220~ion transmembrane transport
GOTERM_BP_ALL	GO:0055085~transmembrane transport
GOTERM_BP_ALL	GO:0048878~chemical homeostasis
GOTERM_BP_ALL	GO:0006816~calcium ion transport
GOTERM_BP_ALL	GO:0034765~regulation of ion transmembrane transport
GOTERM_BP_ALL	GO:0098660~inorganic ion transmembrane transport
GOTERM_BP_ALL	GO:0019725~cellular homeostasis
GOTERM_BP_ALL	GO:0070838~divalent metal ion transport
GOTERM_BP_ALL	GO:0072511~divalent inorganic cation transport
GOTERM_BP_ALL	GO:0006812~cation transport
GOTERM_BP_ALL	GO:0098739~import across plasma membrane
GOTERM_BP_ALL	GO:0030001~metal ion transport

GOTERM_BP_ALL	GO:1902656~calcium ion import into cytosol
GOTERM_BP_ALL	GO:0097553~calcium ion transmembrane import into cytosol
GOTERM_BP_ALL	GO:0070509~calcium ion import
GOTERM_BP_ALL	GO:0098659~inorganic cation import into cell
GOTERM_BP_ALL	GO:0099587~inorganic ion import into cell
GOTERM_BP_ALL	GO:0043270~positive regulation of ion transport
GOTERM_BP_ALL	GO:0055082~cellular chemical homeostasis
GOTERM_BP_ALL	GO:0098703~calcium ion import across plasma membrane
GOTERM_BP_ALL	GO:1990035~calcium ion import into cell
GOTERM_BP_ALL	GO:0098657~import into cell
GOTERM_BP_ALL	GO:0060402~calcium ion transport into cytosol
GOTERM_BP_ALL	GO:0051924~regulation of calcium ion transport
GOTERM_BP_ALL	GO:0070588~calcium ion transmembrane transport
GOTERM_BP_ALL	GO:0060401~cytosolic calcium ion transport
GOTERM_BP_ALL	GO:0007204~positive regulation of cytosolic calcium ion concentration
GOTERM_BP_ALL	GO:0098655~cation transmembrane transport
GOTERM_BP_ALL	GO:1904062~regulation of cation transmembrane transport
GOTERM_BP_ALL	GO:0019226~transmission of nerve impulse
GOTERM_BP_ALL	GO:0051480~regulation of cytosolic calcium ion concentration
GOTERM_BP_ALL	GO:0051928~positive regulation of calcium ion transport
GOTERM_BP_ALL	GO:0098662~inorganic cation transmembrane transport
GOTERM_BP_ALL	GO:0086010~membrane depolarization during action potential
GOTERM_BP_ALL	GO:0051899~membrane depolarization
GOTERM_BP_ALL	GO:0019228~neuronal action potential
GOTERM_BP_ALL	GO:0035637~multicellular organismal signaling
GOTERM_BP_ALL	GO:0010959~regulation of metal ion transport
GOTERM_BP_ALL	GO:0030003~cellular cation homeostasis
GOTERM_BP_ALL	GO:0006875~cellular metal ion homeostasis
GOTERM_BP_ALL	GO:0050801~ion homeostasis
GOTERM_BP_ALL	GO:0006873~cellular ion homeostasis
GOTERM_BP_ALL	GO:0035725~sodium ion transmembrane transport
GOTERM_BP_ALL	GO:0006874~cellular calcium ion homeostasis
GOTERM_BP_ALL	GO:0015672~monovalent inorganic cation transport
GOTERM_BP_ALL	GO:0055074~calcium ion homeostasis
GOTERM_BP_ALL	GO:0072503~cellular divalent inorganic cation homeostasis
GOTERM_BP_ALL	GO:0090279~regulation of calcium ion import
GOTERM_BP_ALL	GO:0055080~cation homeostasis
GOTERM_BP_ALL	GO:0055065~metal ion homeostasis
GOTERM_BP_ALL	GO:0098771~inorganic ion homeostasis
GOTERM_BP_ALL	GO:0001508~action potential
GOTERM_BP_ALL	GO:0072507~divalent inorganic cation homeostasis
GOTERM_BP_ALL	GO:0006814~sodium ion transport
GOTERM_BP_ALL	GO:1903169~regulation of calcium ion transmembrane transport
GOTERM_BP_ALL	GO:0010522~regulation of calcium ion transport into cytosol
GOTERM_BP_ALL	GO:0019722~calcium-mediated signaling
GOTERM_BP_ALL	GO:2000021~regulation of ion homeostasis
Annotation Cluster 95	Enrichment Score: 1.820487539376256
Category	Term
GOTERM_BP_ALL	GO:0010717~regulation of epithelial to mesenchymal transition
GOTERM_BP_ALL	GO:0001837~epithelial to mesenchymal transition

GOTERM_BP_ALL	GO:0044344~cellular response to fibroblast growth factor stimulus
GOTERM_BP_ALL	GO:0071774~response to fibroblast growth factor
GOTERM_BP_ALL	GO:0008543~fibroblast growth factor receptor signaling pathway
Annotation Cluster 96	Enrichment Score: 1.8147629252476964
Category	Term
GOTERM_BP_ALL	GO:0010038~response to metal ion
GOTERM_BP_ALL	GO:0051592~response to calcium ion
GOTERM_BP_ALL	GO:0071277~cellular response to calcium ion
GOTERM_BP_ALL	GO:0071248~cellular response to metal ion
GOTERM_BP_ALL	GO:0071241~cellular response to inorganic substance
Annotation Cluster 97	Enrichment Score: 1.7922777826229757
Category	Term
GOTERM_BP_ALL	GO:0031647~regulation of protein stability
GOTERM_BP_ALL	GO:0031329~regulation of cellular catabolic process
GOTERM_BP_ALL	GO:0031396~regulation of protein ubiquitination
GOTERM_BP_ALL	GO:0009894~regulation of catabolic process
GOTERM_BP_ALL	GO:0031330~negative regulation of cellular catabolic process
GOTERM_BP_ALL	GO:1903320~regulation of protein modification by small protein conjugation or removal
GOTERM_BP_ALL	GO:0009895~negative regulation of catabolic process
GOTERM_BP_ALL	GO:2000058~regulation of protein ubiquitination involved in ubiquitin-dependent protein catabolic process
GOTERM_BP_ALL	GO:0030162~regulation of proteolysis
GOTERM_BP_ALL	GO:1903051~negative regulation of proteolysis involved in cellular protein catabolic process
GOTERM_BP_ALL	GO:1903050~regulation of proteolysis involved in cellular protein catabolic process
GOTERM_BP_ALL	GO:0050821~protein stabilization
GOTERM_BP_ALL	GO:0042787~protein ubiquitination involved in ubiquitin-dependent protein catabolic process
GOTERM_BP_ALL	GO:1903363~negative regulation of cellular protein catabolic process
GOTERM_BP_ALL	GO:0042176~regulation of protein catabolic process
GOTERM_BP_ALL	GO:0042177~negative regulation of protein catabolic process
GOTERM_BP_ALL	GO:1903362~regulation of cellular protein catabolic process
GOTERM_BP_ALL	GO:0045861~negative regulation of proteolysis
GOTERM_BP_ALL	GO:0006511~ubiquitin-dependent protein catabolic process
GOTERM_BP_ALL	GO:0019941~modification-dependent protein catabolic process
GOTERM_BP_ALL	GO:0051438~regulation of ubiquitin-protein transferase activity
GOTERM_BP_ALL	GO:0043632~modification-dependent macromolecule catabolic process
GOTERM_BP_ALL	GO:0009896~positive regulation of catabolic process
GOTERM_BP_ALL	GO:1903321~negative regulation of protein modification by small protein conjugation or removal
GOTERM_BP_ALL	GO:0032446~protein modification by small protein conjugation
GOTERM_BP_ALL	GO:0016567~protein ubiquitination
GOTERM_BP_ALL	GO:0051603~proteolysis involved in cellular protein catabolic process
GOTERM_BP_ALL	GO:0031331~positive regulation of cellular catabolic process
GOTERM_BP_ALL	GO:0032435~negative regulation of proteasomal ubiquitin-dependent protein catabolic process
GOTERM_BP_ALL	GO:0031398~positive regulation of protein ubiquitination

GOTERM_BP_ALL	GO:0031397~negative regulation of protein ubiquitination
GOTERM_BP_ALL	GO:0044257~cellular protein catabolic process
GOTERM_BP_ALL	GO:0044248~cellular catabolic process
GOTERM_BP_ALL	GO:1903322~positive regulation of protein modification by small protein conjugation or removal
GOTERM_BP_ALL	GO:0043161~proteasome-mediated ubiquitin-dependent protein catabolic process
GOTERM_BP_ALL	GO:0030163~protein catabolic process
GOTERM_BP_ALL	GO:1901799~negative regulation of proteasomal protein catabolic process
GOTERM_BP_ALL	GO:0070647~protein modification by small protein conjugation or removal
GOTERM_BP_ALL	GO:0006508~proteolysis
GOTERM_BP_ALL	GO:0044265~cellular macromolecule catabolic process
GOTERM_BP_ALL	GO:0010498~proteasomal protein catabolic process
GOTERM_BP_ALL	GO:0009057~macromolecule catabolic process
GOTERM_BP_ALL	GO:0009056~catabolic process
GOTERM_BP_ALL	GO:0061136~regulation of proteasomal protein catabolic process
GOTERM_BP_ALL	GO:1901575~organic substance catabolic process
GOTERM_BP_ALL	GO:0032434~regulation of proteasomal ubiquitin-dependent protein catabolic process
GOTERM_BP_ALL	GO:0045732~positive regulation of protein catabolic process
GOTERM_BP_ALL	GO:0000209~protein polyubiquitination
Annotation Cluster 98	Enrichment Score: 1.7866742572652332
Category	Term
GOTERM_BP_ALL	GO:0001889~liver development
GOTERM_BP_ALL	GO:0061008~hepaticobiliary system development
GOTERM_BP_ALL	GO:0001824~blastocyst development
Annotation Cluster 99	Enrichment Score: 1.7718513613362763
Category	Term
GOTERM_BP_ALL	GO:0070848~response to growth factor
GOTERM_BP_ALL	GO:0071363~cellular response to growth factor stimulus
GOTERM_BP_ALL	GO:0090287~regulation of cellular response to growth factor stimulus
GOTERM_BP_ALL	GO:0007167~enzyme linked receptor protein signaling pathway
GOTERM_BP_ALL	GO:0017015~regulation of transforming growth factor beta receptor signaling pathway
GOTERM_BP_ALL	GO:1903844~regulation of cellular response to transforming growth factor beta stimulus
GOTERM_BP_ALL	GO:0071560~cellular response to transforming growth factor beta stimulus
GOTERM_BP_ALL	GO:0071559~response to transforming growth factor beta
GOTERM_BP_ALL	GO:0090092~regulation of transmembrane receptor protein serine/threonine kinase signaling pathway
GOTERM_BP_ALL	GO:0090288~negative regulation of cellular response to growth factor stimulus
GOTERM_BP_ALL	GO:0007178~transmembrane receptor protein serine/threonine kinase signaling pathway
GOTERM_BP_ALL	GO:0090100~positive regulation of transmembrane receptor protein serine/threonine kinase signaling pathway
GOTERM_BP_ALL	GO:0007179~transforming growth factor beta receptor signaling pathway
GOTERM_BP_ALL	GO:0030512~negative regulation of transforming growth factor beta receptor signaling pathway

GOTERM_BP_ALL	GO:1903845~negative regulation of cellular response to transforming growth factor beta stimulus
GOTERM_BP_ALL	GO:0090101~negative regulation of transmembrane receptor protein serine/threonine kinase signaling pathway
Annotation Cluster 100	Enrichment Score: 1.7502768762184833
Category	Term
GOTERM_BP_ALL	GO:1901796~regulation of signal transduction by p53 class mediator
GOTERM_BP_ALL	GO:0043516~regulation of DNA damage response, signal transduction by p53 class mediator
GOTERM_BP_ALL	GO:0051053~negative regulation of DNA metabolic process
GOTERM_BP_ALL	GO:0072331~signal transduction by p53 class mediator
GOTERM_BP_ALL	GO:1902253~regulation of intrinsic apoptotic signaling pathway by p53 class mediator
GOTERM_BP_ALL	GO:0030330~DNA damage response, signal transduction by p53 class mediator
GOTERM_BP_ALL	GO:0072332~intrinsic apoptotic signaling pathway by p53 class mediator
GOTERM_BP_ALL	GO:0042770~signal transduction in response to DNA damage
Annotation Cluster 101	Enrichment Score: 1.7412291660892067
Category	Term
GOTERM_BP_ALL	GO:0007097~nuclear migration
GOTERM_BP_ALL	GO:0040023~establishment of nucleus localization
GOTERM_BP_ALL	GO:0051647~nucleus localization
Annotation Cluster 102	Enrichment Score: 1.7399214424649845
Category	Term
GOTERM_BP_ALL	GO:0060348~bone development
GOTERM_BP_ALL	GO:0060349~bone morphogenesis
GOTERM_BP_ALL	GO:0031214~biomineral tissue development
Annotation Cluster 103	Enrichment Score: 1.7349505039219226
Category	Term
GOTERM_BP_ALL	GO:0010769~regulation of cell morphogenesis involved in differentiation
GOTERM_BP_ALL	GO:0022604~regulation of cell morphogenesis
GOTERM_BP_ALL	GO:0050770~regulation of axonogenesis
GOTERM_BP_ALL	GO:0010770~positive regulation of cell morphogenesis involved in differentiation
GOTERM_BP_ALL	GO:0048639~positive regulation of developmental growth
GOTERM_BP_ALL	GO:0048588~developmental cell growth
GOTERM_BP_ALL	GO:0031346~positive regulation of cell projection organization
GOTERM_BP_ALL	GO:0060560~developmental growth involved in morphogenesis
GOTERM_BP_ALL	GO:0008361~regulation of cell size
GOTERM_BP_ALL	GO:0050772~positive regulation of axonogenesis
GOTERM_BP_ALL	GO:0045773~positive regulation of axon extension
GOTERM_BP_ALL	GO:1990138~neuron projection extension
GOTERM_BP_ALL	GO:0090066~regulation of anatomical structure size
GOTERM_BP_ALL	GO:0010976~positive regulation of neuron projection development
GOTERM_BP_ALL	GO:0030516~regulation of axon extension
GOTERM_BP_ALL	GO:0061387~regulation of extent of cell growth
GOTERM_BP_ALL	GO:0048675~axon extension

GOTERM_BP_ALL	GO:0032535~regulation of cellular component size
Annotation Cluster 104	Enrichment Score: 1.6870177963093773
Category	Term
GOTERM_BP_ALL	GO:0070507~regulation of microtubule cytoskeleton organization
GOTERM_BP_ALL	GO:0032886~regulation of microtubule-based process
GOTERM_BP_ALL	GO:0051493~regulation of cytoskeleton organization
GOTERM_BP_ALL	GO:0031109~microtubule polymerization or depolymerization
GOTERM_BP_ALL	GO:0031110~regulation of microtubule polymerization or depolymerization
GOTERM_BP_ALL	GO:0007017~microtubule-based process
GOTERM_BP_ALL	GO:0000226~microtubule cytoskeleton organization
GOTERM_BP_ALL	GO:1902903~regulation of supramolecular fiber organization
GOTERM_BP_ALL	GO:0031112~positive regulation of microtubule polymerization or depolymerization
GOTERM_BP_ALL	GO:0007051~spindle organization
GOTERM_BP_ALL	GO:1902905~positive regulation of supramolecular fiber organization
GOTERM_BP_ALL	GO:0051495~positive regulation of cytoskeleton organization
Annotation Cluster 105	Enrichment Score: 1.6571052173942102
Category	Term
GOTERM_BP_ALL	GO:0043535~regulation of blood vessel endothelial cell migration
GOTERM_BP_ALL	GO:0043534~blood vessel endothelial cell migration
GOTERM_BP_ALL	GO:0001936~regulation of endothelial cell proliferation
GOTERM_BP_ALL	GO:0101023~vascular endothelial cell proliferation
GOTERM_BP_ALL	GO:0001935~endothelial cell proliferation
GOTERM_BP_ALL	GO:1905562~regulation of vascular endothelial cell proliferation
GOTERM_BP_ALL	GO:0001938~positive regulation of endothelial cell proliferation
GOTERM_BP_ALL	GO:1905564~positive regulation of vascular endothelial cell proliferation
GOTERM_BP_ALL	GO:0043536~positive regulation of blood vessel endothelial cell migration
GOTERM_BP_ALL	GO:0001937~negative regulation of endothelial cell proliferation
GOTERM_BP_ALL	GO:0001892~embryonic placenta development
GOTERM_BP_ALL	GO:0001890~placenta development
GOTERM_BP_ALL	GO:0043405~regulation of MAP kinase activity
Annotation Cluster 106	Enrichment Score: 1.6435145855843323
Category	Term
GOTERM_BP_ALL	GO:0007548~sex differentiation
GOTERM_BP_ALL	GO:0046661~male sex differentiation
GOTERM_BP_ALL	GO:0008406~gonad development
GOTERM_BP_ALL	GO:0045137~development of primary sexual characteristics
GOTERM_BP_ALL	GO:0046660~female sex differentiation
GOTERM_BP_ALL	GO:0030522~intracellular receptor signaling pathway
GOTERM_BP_ALL	GO:0009566~fertilization
GOTERM_BP_ALL	GO:0008584~male gonad development
GOTERM_BP_ALL	GO:0046546~development of primary male sexual characteristics
GOTERM_BP_ALL	GO:0008585~female gonad development
GOTERM_BP_ALL	GO:0042698~ovulation cycle
GOTERM_BP_ALL	GO:0046545~development of primary female sexual characteristics
GOTERM_BP_ALL	GO:0022602~ovulation cycle process
Annotation Cluster 107	Enrichment Score: 1.6365068421070934

Category	Term
GOTERM_BP_ALL	GO:0030316~osteoclast differentiation
GOTERM_BP_ALL	GO:0008544~epidermis development
GOTERM_BP_ALL	GO:0009913~epidermal cell differentiation
Annotation Cluster 108	Enrichment Score: 1.6104218440073748
Category	Term
GOTERM_BP_ALL	GO:0002366~leukocyte activation involved in immune response
GOTERM_BP_ALL	GO:0002263~cell activation involved in immune response
GOTERM_BP_ALL	GO:0010595~positive regulation of endothelial cell migration
GOTERM_BP_ALL	GO:0010634~positive regulation of epithelial cell migration
GOTERM_BP_ALL	GO:0042093~T-helper cell differentiation
GOTERM_BP_ALL	GO:0046632~alpha-beta T cell differentiation
GOTERM_BP_ALL	GO:0046631~alpha-beta T cell activation
GOTERM_BP_ALL	GO:0002286~T cell activation involved in immune response
GOTERM_BP_ALL	GO:0002294~CD4-positive, alpha-beta T cell differentiation involved in immune response
GOTERM_BP_ALL	GO:0002287~alpha-beta T cell activation involved in immune response
GOTERM_BP_ALL	GO:0002293~alpha-beta T cell differentiation involved in immune response
GOTERM_BP_ALL	GO:0002292~T cell differentiation involved in immune response
GOTERM_BP_ALL	GO:0002285~lymphocyte activation involved in immune response
GOTERM_BP_ALL	GO:0043367~CD4-positive, alpha-beta T cell differentiation
GOTERM_BP_ALL	GO:0035710~CD4-positive, alpha-beta T cell activation
GOTERM_BP_ALL	GO:0034612~response to tumor necrosis factor
GOTERM_BP_ALL	GO:0071356~cellular response to tumor necrosis factor
GOTERM_BP_ALL	GO:0001819~positive regulation of cytokine production
Annotation Cluster 109	Enrichment Score: 1.598048371976269
Category	Term
GOTERM_BP_ALL	GO:0016202~regulation of striated muscle tissue development
GOTERM_BP_ALL	GO:0051147~regulation of muscle cell differentiation
GOTERM_BP_ALL	GO:0048634~regulation of muscle organ development
GOTERM_BP_ALL	GO:1901861~regulation of muscle tissue development
GOTERM_BP_ALL	GO:0048638~regulation of developmental growth
GOTERM_BP_ALL	GO:0051148~negative regulation of muscle cell differentiation
GOTERM_BP_ALL	GO:1901862~negative regulation of muscle tissue development
GOTERM_BP_ALL	GO:0035051~cardiocyte differentiation
GOTERM_BP_ALL	GO:0035265~organ growth
GOTERM_BP_ALL	GO:0048738~cardiac muscle tissue development
GOTERM_BP_ALL	GO:2000106~regulation of leukocyte apoptotic process
GOTERM_BP_ALL	GO:0046620~regulation of organ growth
GOTERM_BP_ALL	GO:0045843~negative regulation of striated muscle tissue development
GOTERM_BP_ALL	GO:0048635~negative regulation of muscle organ development
GOTERM_BP_ALL	GO:0055021~regulation of cardiac muscle tissue growth
GOTERM_BP_ALL	GO:0055007~cardiac muscle cell differentiation
GOTERM_BP_ALL	GO:0055022~negative regulation of cardiac muscle tissue growth
GOTERM_BP_ALL	GO:0061117~negative regulation of heart growth
GOTERM_BP_ALL	GO:0071887~leukocyte apoptotic process
GOTERM_BP_ALL	GO:0060420~regulation of heart growth
GOTERM_BP_ALL	GO:0045926~negative regulation of growth
GOTERM_BP_ALL	GO:2000108~positive regulation of leukocyte apoptotic process

GOTERM_BP_ALL	GO:0033032~regulation of myeloid cell apoptotic process
GOTERM_BP_ALL	GO:0046621~negative regulation of organ growth
GOTERM_BP_ALL	GO:0055024~regulation of cardiac muscle tissue development
GOTERM_BP_ALL	GO:0055017~cardiac muscle tissue growth
GOTERM_BP_ALL	GO:0055026~negative regulation of cardiac muscle tissue development
GOTERM_BP_ALL	GO:2000725~regulation of cardiac muscle cell differentiation
GOTERM_BP_ALL	GO:0060419~heart growth
GOTERM_BP_ALL	GO:0048660~regulation of smooth muscle cell proliferation
GOTERM_BP_ALL	GO:0051153~regulation of striated muscle cell differentiation
GOTERM_BP_ALL	GO:1905207~regulation of cardiocyte differentiation
GOTERM_BP_ALL	GO:0070228~regulation of lymphocyte apoptotic process
GOTERM_BP_ALL	GO:0014855~striated muscle cell proliferation
GOTERM_BP_ALL	GO:0033002~muscle cell proliferation
GOTERM_BP_ALL	GO:0070227~lymphocyte apoptotic process
GOTERM_BP_ALL	GO:0048659~smooth muscle cell proliferation
GOTERM_BP_ALL	GO:1904705~regulation of vascular smooth muscle cell proliferation
GOTERM_BP_ALL	GO:1990874~vascular smooth muscle cell proliferation
GOTERM_BP_ALL	GO:0048640~negative regulation of developmental growth
Annotation Cluster 110	Enrichment Score: 1.577021468162972
Category	Term
GOTERM_BP_ALL	GO:0001824~blastocyst development
GOTERM_BP_ALL	GO:0001835~blastocyst hatching
GOTERM_BP_ALL	GO:0071684~organism emergence from protective structure
GOTERM_BP_ALL	GO:0035188~hatching
Annotation Cluster 111	Enrichment Score: 1.5464086022283383
Category	Term
GOTERM_BP_ALL	GO:0002366~leukocyte activation involved in immune response
GOTERM_BP_ALL	GO:0002263~cell activation involved in immune response
GOTERM_BP_ALL	GO:0045055~regulated exocytosis
GOTERM_BP_ALL	GO:0006887~exocytosis
GOTERM_BP_ALL	GO:1903305~regulation of regulated secretory pathway
GOTERM_BP_ALL	GO:0043299~leukocyte degranulation
GOTERM_BP_ALL	GO:0043303~mast cell degranulation
GOTERM_BP_ALL	GO:0002448~mast cell mediated immunity
GOTERM_BP_ALL	GO:0002275~myeloid cell activation involved in immune response
GOTERM_BP_ALL	GO:0002279~mast cell activation involved in immune response
GOTERM_BP_ALL	GO:0002444~myeloid leukocyte mediated immunity
GOTERM_BP_ALL	GO:0002274~myeloid leukocyte activation
GOTERM_BP_ALL	GO:0045576~mast cell activation
GOTERM_BP_ALL	GO:0043304~regulation of mast cell degranulation
GOTERM_BP_ALL	GO:0032418~lysosome localization
GOTERM_BP_ALL	GO:0033006~regulation of mast cell activation involved in immune response
GOTERM_BP_ALL	GO:0002252~immune effector process
GOTERM_BP_ALL	GO:0033003~regulation of mast cell activation
GOTERM_BP_ALL	GO:0043300~regulation of leukocyte degranulation
GOTERM_BP_ALL	GO:0002703~regulation of leukocyte mediated immunity
GOTERM_BP_ALL	GO:0002443~leukocyte mediated immunity
GOTERM_BP_ALL	GO:0002886~regulation of myeloid leukocyte mediated immunity
GOTERM_BP_ALL	GO:0050776~regulation of immune response

GOTERM_BP_ALL	GO:0002697~regulation of immune effector process
GOTERM_BP_ALL	GO:0002705~positive regulation of leukocyte mediated immunity
GOTERM_BP_ALL	GO:0050778~positive regulation of immune response
GOTERM_BP_ALL	GO:0002699~positive regulation of immune effector process
GOTERM_BP_ALL	GO:0006955~immune response
Annotation Cluster 112	Enrichment Score: 1.5334299524846815
Category	Term
GOTERM_BP_ALL	GO:1903827~regulation of cellular protein localization
GOTERM_BP_ALL	GO:0032880~regulation of protein localization
GOTERM_BP_ALL	GO:1903829~positive regulation of cellular protein localization
GOTERM_BP_ALL	GO:1904951~positive regulation of establishment of protein localization
GOTERM_BP_ALL	GO:0090087~regulation of peptide transport
GOTERM_BP_ALL	GO:0070201~regulation of establishment of protein localization
GOTERM_BP_ALL	GO:0051222~positive regulation of protein transport
GOTERM_BP_ALL	GO:0051223~regulation of protein transport
GOTERM_BP_ALL	GO:0002791~regulation of peptide secretion
GOTERM_BP_ALL	GO:0090316~positive regulation of intracellular protein transport
GOTERM_BP_ALL	GO:0050708~regulation of protein secretion
GOTERM_BP_ALL	GO:0033157~regulation of intracellular protein transport
GOTERM_BP_ALL	GO:1903533~regulation of protein targeting
Annotation Cluster 113	Enrichment Score: 1.497911234230321
Category	Term
GOTERM_BP_ALL	GO:0051216~cartilage development
GOTERM_BP_ALL	GO:0036075~replacement ossification
GOTERM_BP_ALL	GO:0001958~endochondral ossification
GOTERM_BP_ALL	GO:0060349~bone morphogenesis
GOTERM_BP_ALL	GO:0060350~endochondral bone morphogenesis
Annotation Cluster 114	Enrichment Score: 1.460966237532247
Category	Term
GOTERM_BP_ALL	GO:0007017~microtubule-based process
GOTERM_BP_ALL	GO:0010970~transport along microtubule
GOTERM_BP_ALL	GO:0099111~microtubule-based transport
GOTERM_BP_ALL	GO:0008088~axo-dendritic transport
GOTERM_BP_ALL	GO:0030705~cytoskeleton-dependent intracellular transport
GOTERM_BP_ALL	GO:0072384~organelle transport along microtubule
GOTERM_BP_ALL	GO:0007018~microtubule-based movement
Annotation Cluster 115	Enrichment Score: 1.4274943464718395
Category	Term
GOTERM_BP_ALL	GO:2001236~regulation of extrinsic apoptotic signaling pathway
GOTERM_BP_ALL	GO:0097191~extrinsic apoptotic signaling pathway
GOTERM_BP_ALL	GO:2001237~negative regulation of extrinsic apoptotic signaling pathway
GOTERM_BP_ALL	GO:2001239~regulation of extrinsic apoptotic signaling pathway in absence of ligand
GOTERM_BP_ALL	GO:0008625~extrinsic apoptotic signaling pathway via death domain receptors
Annotation Cluster 116	Enrichment Score: 1.385646436321252

Category	Term
GOTERM_BP_ALL	GO:0035051~cardiocyte differentiation
GOTERM_BP_ALL	GO:0055002~striated muscle cell development
GOTERM_BP_ALL	GO:0048738~cardiac muscle tissue development
GOTERM_BP_ALL	GO:0055001~muscle cell development
GOTERM_BP_ALL	GO:0032204~regulation of telomere maintenance
GOTERM_BP_ALL	GO:0010611~regulation of cardiac muscle hypertrophy
GOTERM_BP_ALL	GO:0055021~regulation of cardiac muscle tissue growth
GOTERM_BP_ALL	GO:0043500~muscle adaptation
GOTERM_BP_ALL	GO:0014743~regulation of muscle hypertrophy
GOTERM_BP_ALL	GO:0055007~cardiac muscle cell differentiation
GOTERM_BP_ALL	GO:0060420~regulation of heart growth
GOTERM_BP_ALL	GO:0010614~negative regulation of cardiac muscle hypertrophy
GOTERM_BP_ALL	GO:0055013~cardiac muscle cell development
GOTERM_BP_ALL	GO:0014741~negative regulation of muscle hypertrophy
GOTERM_BP_ALL	GO:0055024~regulation of cardiac muscle tissue development
GOTERM_BP_ALL	GO:0055006~cardiac cell development
GOTERM_BP_ALL	GO:0055017~cardiac muscle tissue growth
GOTERM_BP_ALL	GO:0043502~regulation of muscle adaptation
GOTERM_BP_ALL	GO:0003300~cardiac muscle hypertrophy
GOTERM_BP_ALL	GO:0060419~heart growth
GOTERM_BP_ALL	GO:0014897~striated muscle hypertrophy
GOTERM_BP_ALL	GO:0014896~muscle hypertrophy
GOTERM_BP_ALL	GO:0014888~striated muscle adaptation
GOTERM_BP_ALL	GO:0044057~regulation of system process
GOTERM_BP_ALL	GO:0003012~muscle system process
GOTERM_BP_ALL	GO:0090257~regulation of muscle system process
Annotation Cluster 117	Enrichment Score: 1.377385045820297
Category	Term
GOTERM_BP_ALL	GO:0045580~regulation of T cell differentiation
GOTERM_BP_ALL	GO:0045619~regulation of lymphocyte differentiation
GOTERM_BP_ALL	GO:0046632~alpha-beta T cell differentiation
GOTERM_BP_ALL	GO:0046631~alpha-beta T cell activation
GOTERM_BP_ALL	GO:0046637~regulation of alpha-beta T cell differentiation
GOTERM_BP_ALL	GO:0046634~regulation of alpha-beta T cell activation
Annotation Cluster 118	Enrichment Score: 1.3120754978819988
Category	Term
GOTERM_BP_ALL	GO:0002065~columnar/cuboidal epithelial cell differentiation
GOTERM_BP_ALL	GO:0002067~glandular epithelial cell differentiation
GOTERM_BP_ALL	GO:0002066~columnar/cuboidal epithelial cell development
GOTERM_BP_ALL	GO:0002064~epithelial cell development
Annotation Cluster 119	Enrichment Score: 1.305195674204711
Category	Term
GOTERM_BP_ALL	GO:0010810~regulation of cell-substrate adhesion
GOTERM_BP_ALL	GO:0031589~cell-substrate adhesion
GOTERM_BP_ALL	GO:0010812~negative regulation of cell-substrate adhesion
GOTERM_BP_ALL	GO:0001952~regulation of cell-matrix adhesion
GOTERM_BP_ALL	GO:0001953~negative regulation of cell-matrix adhesion

GOTERM_BP_ALL	GO:0007160~cell-matrix adhesion
GOTERM_BP_ALL	GO:0007162~negative regulation of cell adhesion
GOTERM_BP_ALL	GO:0061041~regulation of wound healing
GOTERM_BP_ALL	GO:1903034~regulation of response to wounding
Annotation Cluster 120	Enrichment Score: 1.2949675632437572
Category	Term
GOTERM_BP_ALL	GO:0097193~intrinsic apoptotic signaling pathway
GOTERM_BP_ALL	GO:2001242~regulation of intrinsic apoptotic signaling pathway
GOTERM_BP_ALL	GO:2001235~positive regulation of apoptotic signaling pathway
GOTERM_BP_ALL	GO:1903146~regulation of mitophagy
GOTERM_BP_ALL	GO:0010821~regulation of mitochondrion organization
GOTERM_BP_ALL	GO:2001244~positive regulation of intrinsic apoptotic signaling pathway
GOTERM_BP_ALL	GO:0008637~apoptotic mitochondrial changes
GOTERM_BP_ALL	GO:0010822~positive regulation of mitochondrion organization
GOTERM_BP_ALL	GO:0090199~regulation of release of cytochrome c from mitochondria
GOTERM_BP_ALL	GO:1902108~regulation of mitochondrial membrane permeability involved in apoptotic process
GOTERM_BP_ALL	GO:0001836~release of cytochrome c from mitochondria
GOTERM_BP_ALL	GO:0046902~regulation of mitochondrial membrane permeability
GOTERM_BP_ALL	GO:0070585~protein localization to mitochondrion
GOTERM_BP_ALL	GO:0007005~mitochondrion organization
GOTERM_BP_ALL	GO:0090559~regulation of membrane permeability
GOTERM_BP_ALL	GO:0072655~establishment of protein localization to mitochondrion
GOTERM_BP_ALL	GO:0007006~mitochondrial membrane organization
GOTERM_BP_ALL	GO:0006839~mitochondrial transport
Annotation Cluster 121	Enrichment Score: 1.2797351371128125
Category	Term
GOTERM_BP_ALL	GO:0045739~positive regulation of DNA repair
GOTERM_BP_ALL	GO:2001022~positive regulation of response to DNA damage stimulus
GOTERM_BP_ALL	GO:0016444~somatic cell DNA recombination
GOTERM_BP_ALL	GO:0002562~somatic diversification of immune receptors via germline recombination within a single locus
GOTERM_BP_ALL	GO:0002200~somatic diversification of immune receptors
GOTERM_BP_ALL	GO:0006310~DNA recombination
GOTERM_BP_ALL	GO:0000724~double-strand break repair via homologous recombination
GOTERM_BP_ALL	GO:0000725~recombinational repair
Annotation Cluster 122	Enrichment Score: 1.2781425643995248
Category	Term
GOTERM_BP_ALL	GO:0061024~membrane organization
GOTERM_BP_ALL	GO:0044802~single-organism membrane organization
GOTERM_BP_ALL	GO:0010256~endomembrane system organization
GOTERM_BP_ALL	GO:1990778~protein localization to cell periphery
GOTERM_BP_ALL	GO:0007009~plasma membrane organization
GOTERM_BP_ALL	GO:0072657~protein localization to membrane
GOTERM_BP_ALL	GO:0072659~protein localization to plasma membrane
Annotation Cluster 123	Enrichment Score: 1.2781062911232908
Category	Term

GOTERM_BP_ALL	GO:0010810~regulation of cell-substrate adhesion
GOTERM_BP_ALL	GO:0097435~supramolecular fiber organization
GOTERM_BP_ALL	GO:0051493~regulation of cytoskeleton organization
GOTERM_BP_ALL	GO:0001738~morphogenesis of a polarized epithelium
GOTERM_BP_ALL	GO:0007010~cytoskeleton organization
GOTERM_BP_ALL	GO:0030100~regulation of endocytosis
GOTERM_BP_ALL	GO:0031589~cell-substrate adhesion
GOTERM_BP_ALL	GO:0034332~adherens junction organization
GOTERM_BP_ALL	GO:0030029~actin filament-based process
GOTERM_BP_ALL	GO:0001952~regulation of cell-matrix adhesion
GOTERM_BP_ALL	GO:0034333~adherens junction assembly
GOTERM_BP_ALL	GO:0030036~actin cytoskeleton organization
GOTERM_BP_ALL	GO:0051017~actin filament bundle assembly
GOTERM_BP_ALL	GO:0061572~actin filament bundle organization
GOTERM_BP_ALL	GO:0007160~cell-matrix adhesion
GOTERM_BP_ALL	GO:0032231~regulation of actin filament bundle assembly
GOTERM_BP_ALL	GO:0034329~cell junction assembly
GOTERM_BP_ALL	GO:0031032~actomyosin structure organization
GOTERM_BP_ALL	GO:0045216~cell-cell junction organization
GOTERM_BP_ALL	GO:0048259~regulation of receptor-mediated endocytosis
GOTERM_BP_ALL	GO:0051893~regulation of focal adhesion assembly
GOTERM_BP_ALL	GO:0090109~regulation of cell-substrate junction assembly
GOTERM_BP_ALL	GO:1903391~regulation of adherens junction organization
GOTERM_BP_ALL	GO:0034330~cell junction organization
GOTERM_BP_ALL	GO:0032956~regulation of actin cytoskeleton organization
GOTERM_BP_ALL	GO:0032970~regulation of actin filament-based process
GOTERM_BP_ALL	GO:0051492~regulation of stress fiber assembly
GOTERM_BP_ALL	GO:0007044~cell-substrate junction assembly
GOTERM_BP_ALL	GO:0030038~contractile actin filament bundle assembly
GOTERM_BP_ALL	GO:0043149~stress fiber assembly
GOTERM_BP_ALL	GO:1901888~regulation of cell junction assembly
GOTERM_BP_ALL	GO:0007015~actin filament organization
GOTERM_BP_ALL	GO:0007266~Rho protein signal transduction
Annotation Cluster 124	Enrichment Score: 1.2747205124345435
Category	Term
GOTERM_BP_ALL	GO:0043491~protein kinase B signaling
GOTERM_BP_ALL	GO:0051896~regulation of protein kinase B signaling
GOTERM_BP_ALL	GO:0032869~cellular response to insulin stimulus
GOTERM_BP_ALL	GO:0046626~regulation of insulin receptor signaling pathway
GOTERM_BP_ALL	GO:1900076~regulation of cellular response to insulin stimulus
GOTERM_BP_ALL	GO:0008286~insulin receptor signaling pathway
Annotation Cluster 125	Enrichment Score: 1.2717630763930527
Category	Term
GOTERM_BP_ALL	GO:0001704~formation of primary germ layer
GOTERM_BP_ALL	GO:0007498~mesoderm development
GOTERM_BP_ALL	GO:0001707~mesoderm formation
GOTERM_BP_ALL	GO:0048332~mesoderm morphogenesis
Annotation Cluster 126	Enrichment Score: 1.262098971441987

Category	Term
GOTERM_BP_ALL	GO:0071214~cellular response to abiotic stimulus
GOTERM_BP_ALL	GO:0034644~cellular response to UV
GOTERM_BP_ALL	GO:0071478~cellular response to radiation
GOTERM_BP_ALL	GO:0071482~cellular response to light stimulus
GOTERM_BP_ALL	GO:0009411~response to UV
Annotation Cluster 127	Enrichment Score: 1.2467540115696671
Category	Term
GOTERM_BP_ALL	GO:2000045~regulation of G1/S transition of mitotic cell cycle
GOTERM_BP_ALL	GO:0000082~G1/S transition of mitotic cell cycle
GOTERM_BP_ALL	GO:1902807~negative regulation of cell cycle G1/S phase transition
GOTERM_BP_ALL	GO:0045786~negative regulation of cell cycle
GOTERM_BP_ALL	GO:0045930~negative regulation of mitotic cell cycle
GOTERM_BP_ALL	GO:0010948~negative regulation of cell cycle process
GOTERM_BP_ALL	GO:2000134~negative regulation of G1/S transition of mitotic cell cycle
GOTERM_BP_ALL	GO:1901988~negative regulation of cell cycle phase transition
GOTERM_BP_ALL	GO:1901991~negative regulation of mitotic cell cycle phase transition
GOTERM_BP_ALL	GO:0044773~mitotic DNA damage checkpoint
GOTERM_BP_ALL	GO:0044774~mitotic DNA integrity checkpoint
GOTERM_BP_ALL	GO:0000077~DNA damage checkpoint
GOTERM_BP_ALL	GO:0031570~DNA integrity checkpoint
GOTERM_BP_ALL	GO:0007093~mitotic cell cycle checkpoint
GOTERM_BP_ALL	GO:0000075~cell cycle checkpoint
Annotation Cluster 128	Enrichment Score: 1.2110820269825806
Category	Term
GOTERM_BP_ALL	GO:0031109~microtubule polymerization or depolymerization
GOTERM_BP_ALL	GO:0031110~regulation of microtubule polymerization or depolymerization
GOTERM_BP_ALL	GO:1902903~regulation of supramolecular fiber organization
GOTERM_BP_ALL	GO:0046785~microtubule polymerization
GOTERM_BP_ALL	GO:0031113~regulation of microtubule polymerization
GOTERM_BP_ALL	GO:0051258~protein polymerization
GOTERM_BP_ALL	GO:0032271~regulation of protein polymerization
Annotation Cluster 129	Enrichment Score: 1.1814818034786683
Category	Term
GOTERM_BP_ALL	GO:0046661~male sex differentiation
GOTERM_BP_ALL	GO:2000243~positive regulation of reproductive process
GOTERM_BP_ALL	GO:2000241~regulation of reproductive process
Annotation Cluster 130	Enrichment Score: 1.167496300913339
Category	Term
GOTERM_BP_ALL	GO:0050805~negative regulation of synaptic transmission
GOTERM_BP_ALL	GO:0032228~regulation of synaptic transmission, GABAergic
GOTERM_BP_ALL	GO:0046717~acid secretion
GOTERM_BP_ALL	GO:0006865~amino acid transport
GOTERM_BP_ALL	GO:0015718~monocarboxylic acid transport
GOTERM_BP_ALL	GO:0046942~carboxylic acid transport
GOTERM_BP_ALL	GO:0015849~organic acid transport
GOTERM_BP_ALL	GO:0015711~organic anion transport

Annotation Cluster 131	Enrichment Score: 1.1661439330933268
Category	Term
GOTERM_BP_ALL	GO:2000142~regulation of DNA-templated transcription, initiation
GOTERM_BP_ALL	GO:0060260~regulation of transcription initiation from RNA polymerase II promoter
GOTERM_BP_ALL	GO:0006367~transcription initiation from RNA polymerase II promoter
Annotation Cluster 132	Enrichment Score: 1.1589966404036662
Category	Term
GOTERM_BP_ALL	GO:0034504~protein localization to nucleus
GOTERM_BP_ALL	GO:1902593~single-organism nuclear import
GOTERM_BP_ALL	GO:0006606~protein import into nucleus
GOTERM_BP_ALL	GO:0044744~protein targeting to nucleus
GOTERM_BP_ALL	GO:0006886~intracellular protein transport
GOTERM_BP_ALL	GO:0051170~nuclear import
GOTERM_BP_ALL	GO:0006605~protein targeting
GOTERM_BP_ALL	GO:0010823~negative regulation of mitochondrion organization
GOTERM_BP_ALL	GO:0072594~establishment of protein localization to organelle
GOTERM_BP_ALL	GO:0017038~protein import
GOTERM_BP_ALL	GO:0034103~regulation of tissue remodeling
GOTERM_BP_ALL	GO:0006913~nucleocytoplasmic transport
GOTERM_BP_ALL	GO:0051169~nuclear transport
Annotation Cluster 133	Enrichment Score: 1.1532396625136823
Category	Term
GOTERM_BP_ALL	GO:0030199~collagen fibril organization
GOTERM_BP_ALL	GO:0030198~extracellular matrix organization
GOTERM_BP_ALL	GO:0043062~extracellular structure organization
Annotation Cluster 134	Enrichment Score: 1.151534929062636
Category	Term
GOTERM_BP_ALL	GO:0090335~regulation of brown fat cell differentiation
GOTERM_BP_ALL	GO:0045444~fat cell differentiation
GOTERM_BP_ALL	GO:0050873~brown fat cell differentiation
GOTERM_BP_ALL	GO:0045598~regulation of fat cell differentiation
Annotation Cluster 135	Enrichment Score: 1.1330407774939146
Category	Term
GOTERM_BP_ALL	GO:0051937~catecholamine transport
GOTERM_BP_ALL	GO:0015844~monoamine transport
GOTERM_BP_ALL	GO:0050432~catecholamine secretion
GOTERM_BP_ALL	GO:0015850~organic hydroxy compound transport
Annotation Cluster 136	Enrichment Score: 1.1268282549699506
Category	Term
GOTERM_BP_ALL	GO:0010770~positive regulation of cell morphogenesis involved in differentiation
GOTERM_BP_ALL	GO:0050900~leukocyte migration
GOTERM_BP_ALL	GO:0030865~cortical cytoskeleton organization
GOTERM_BP_ALL	GO:0001736~establishment of planar polarity

GOTERM_BP_ALL	GO:0007164~establishment of tissue polarity
Annotation Cluster 137	Enrichment Score: 1.0875471456176335
Category	Term
GOTERM_BP_ALL	GO:0048147~negative regulation of fibroblast proliferation
GOTERM_BP_ALL	GO:0048145~regulation of fibroblast proliferation
GOTERM_BP_ALL	GO:0048144~fibroblast proliferation
Annotation Cluster 138	Enrichment Score: 1.0805701785981823
Category	Term
GOTERM_BP_ALL	GO:1902803~regulation of synaptic vesicle transport
GOTERM_BP_ALL	GO:0070527~platelet aggregation
GOTERM_BP_ALL	GO:0034109~homotypic cell-cell adhesion
GOTERM_BP_ALL	GO:0030168~platelet activation
GOTERM_BP_ALL	GO:0007596~blood coagulation
GOTERM_BP_ALL	GO:0007599~hemostasis
GOTERM_BP_ALL	GO:0050817~coagulation
GOTERM_BP_ALL	GO:0050878~regulation of body fluid levels
Annotation Cluster 139	Enrichment Score: 1.0346767240006902
Category	Term
GOTERM_BP_ALL	GO:0071396~cellular response to lipid
GOTERM_BP_ALL	GO:0048545~response to steroid hormone
GOTERM_BP_ALL	GO:1901654~response to ketone
GOTERM_BP_ALL	GO:0071383~cellular response to steroid hormone stimulus
Annotation Cluster 140	Enrichment Score: 1.020055157513497
Category	Term
GOTERM_BP_ALL	GO:0051781~positive regulation of cell division
GOTERM_BP_ALL	GO:0032467~positive regulation of cytokinesis
GOTERM_BP_ALL	GO:0051302~regulation of cell division
GOTERM_BP_ALL	GO:0032465~regulation of cytokinesis
GOTERM_BP_ALL	GO:0000910~cytokinesis
Annotation Cluster 141	Enrichment Score: 1.0187373105429396
Category	Term
GOTERM_BP_ALL	GO:2000116~regulation of cysteine-type endopeptidase activity
GOTERM_BP_ALL	GO:0051336~regulation of hydrolase activity
GOTERM_BP_ALL	GO:0052548~regulation of endopeptidase activity
GOTERM_BP_ALL	GO:0043281~regulation of cysteine-type endopeptidase activity involved in apoptotic process
GOTERM_BP_ALL	GO:2001056~positive regulation of cysteine-type endopeptidase activity
GOTERM_BP_ALL	GO:0010950~positive regulation of endopeptidase activity
GOTERM_BP_ALL	GO:0051345~positive regulation of hydrolase activity
GOTERM_BP_ALL	GO:0052547~regulation of peptidase activity
GOTERM_BP_ALL	GO:0010952~positive regulation of peptidase activity
GOTERM_BP_ALL	GO:0043154~negative regulation of cysteine-type endopeptidase activity involved in apoptotic process
GOTERM_BP_ALL	GO:0045862~positive regulation of proteolysis
GOTERM_BP_ALL	GO:2000117~negative regulation of cysteine-type endopeptidase activity

GOTERM_BP_ALL	GO:0043280~positive regulation of cysteine-type endopeptidase activity involved in apoptotic process
GOTERM_BP_ALL	GO:0010951~negative regulation of endopeptidase activity
GOTERM_BP_ALL	GO:0051346~negative regulation of hydrolase activity
GOTERM_BP_ALL	GO:0010466~negative regulation of peptidase activity
Annotation Cluster 142	Enrichment Score: 1.0133462939982585
Category	Term
GOTERM_BP_ALL	GO:0010506~regulation of autophagy
GOTERM_BP_ALL	GO:0006914~autophagy
GOTERM_BP_ALL	GO:0016241~regulation of macroautophagy
GOTERM_BP_ALL	GO:1903008~organelle disassembly
GOTERM_BP_ALL	GO:0016239~positive regulation of macroautophagy
GOTERM_BP_ALL	GO:0000422~mitophagy
GOTERM_BP_ALL	GO:0061726~mitochondrion disassembly
GOTERM_BP_ALL	GO:0010508~positive regulation of autophagy
GOTERM_BP_ALL	GO:0016236~macroautophagy
Annotation Cluster 143	Enrichment Score: 1.0010122107743045
Category	Term
GOTERM_BP_ALL	GO:0045197~establishment or maintenance of epithelial cell apical/basal polarity
GOTERM_BP_ALL	GO:0035088~establishment or maintenance of apical/basal cell polarity
GOTERM_BP_ALL	GO:0061245~establishment or maintenance of bipolar cell polarity
Annotation Cluster 144	Enrichment Score: 0.9939202383280086
Category	Term
GOTERM_BP_ALL	GO:0010770~positive regulation of cell morphogenesis involved in differentiation
GOTERM_BP_ALL	GO:0006911~phagocytosis, engulfment
GOTERM_BP_ALL	GO:0099024~plasma membrane invagination
GOTERM_BP_ALL	GO:1900026~positive regulation of substrate adhesion-dependent cell spreading
GOTERM_BP_ALL	GO:0010324~membrane invagination
GOTERM_BP_ALL	GO:0050900~leukocyte migration
GOTERM_BP_ALL	GO:1900024~regulation of substrate adhesion-dependent cell spreading
GOTERM_BP_ALL	GO:0010811~positive regulation of cell-substrate adhesion
GOTERM_BP_ALL	GO:0032956~regulation of actin cytoskeleton organization
GOTERM_BP_ALL	GO:0006909~phagocytosis
GOTERM_BP_ALL	GO:0032970~regulation of actin filament-based process
GOTERM_BP_ALL	GO:0034446~substrate adhesion-dependent cell spreading
GOTERM_BP_ALL	GO:0060491~regulation of cell projection assembly
GOTERM_BP_ALL	GO:0030031~cell projection assembly
Annotation Cluster 145	Enrichment Score: 0.984300478220346
Category	Term
GOTERM_BP_ALL	GO:0071346~cellular response to interferon-gamma
GOTERM_BP_ALL	GO:0034341~response to interferon-gamma
GOTERM_BP_ALL	GO:0045087~innate immune response
Annotation Cluster 146	Enrichment Score: 0.9350044975303342

Category	Term
GOTERM_BP_ALL	GO:0007052~mitotic spindle organization
GOTERM_BP_ALL	GO:1902850~microtubule cytoskeleton organization involved in mitosis
GOTERM_BP_ALL	GO:0007051~spindle organization
Annotation Cluster 147	Enrichment Score: 0.9293366712249611
Category	Term
GOTERM_BP_ALL	GO:0050954~sensory perception of mechanical stimulus
GOTERM_BP_ALL	GO:0007605~sensory perception of sound
GOTERM_BP_ALL	GO:0007600~sensory perception
Annotation Cluster 148	Enrichment Score: 0.926302886838678
Category	Term
GOTERM_BP_ALL	GO:2000377~regulation of reactive oxygen species metabolic process
GOTERM_BP_ALL	GO:1903426~regulation of reactive oxygen species biosynthetic process
GOTERM_BP_ALL	GO:1903409~reactive oxygen species biosynthetic process
GOTERM_BP_ALL	GO:2000378~negative regulation of reactive oxygen species metabolic process
GOTERM_BP_ALL	GO:0072593~reactive oxygen species metabolic process
GOTERM_BP_ALL	GO:2000379~positive regulation of reactive oxygen species metabolic process
Annotation Cluster 149	Enrichment Score: 0.8782832044942449
Category	Term
GOTERM_BP_ALL	GO:0055088~lipid homeostasis
GOTERM_BP_ALL	GO:0046890~regulation of lipid biosynthetic process
GOTERM_BP_ALL	GO:0008610~lipid biosynthetic process
Annotation Cluster 150	Enrichment Score: 0.8781353687507323
Category	Term
GOTERM_BP_ALL	GO:0048024~regulation of mRNA splicing, via spliceosome
GOTERM_BP_ALL	GO:0033119~negative regulation of RNA splicing
GOTERM_BP_ALL	GO:0043484~regulation of RNA splicing
GOTERM_BP_ALL	GO:0050684~regulation of mRNA processing
GOTERM_BP_ALL	GO:1903312~negative regulation of mRNA metabolic process
GOTERM_BP_ALL	GO:1903311~regulation of mRNA metabolic process
GOTERM_BP_ALL	GO:0000381~regulation of alternative mRNA splicing, via spliceosome
GOTERM_BP_ALL	GO:0008380~RNA splicing
GOTERM_BP_ALL	GO:0000377~RNA splicing, via transesterification reactions with bulged adenosine as nucleophile
GOTERM_BP_ALL	GO:0000398~mRNA splicing, via spliceosome
GOTERM_BP_ALL	GO:0000375~RNA splicing, via transesterification reactions
GOTERM_BP_ALL	GO:0006397~mRNA processing
GOTERM_BP_ALL	GO:0006396~RNA processing
GOTERM_BP_ALL	GO:0016071~mRNA metabolic process
Annotation Cluster 151	Enrichment Score: 0.8757353659517456
Category	Term
GOTERM_BP_ALL	GO:0018108~peptidyl-tyrosine phosphorylation
GOTERM_BP_ALL	GO:0018212~peptidyl-tyrosine modification
GOTERM_BP_ALL	GO:0010675~regulation of cellular carbohydrate metabolic process

GOTERM_BP_ALL	GO:0050730~regulation of peptidyl-tyrosine phosphorylation
GOTERM_BP_ALL	GO:0050731~positive regulation of peptidyl-tyrosine phosphorylation
Annotation Cluster 152	Enrichment Score: 0.8717246355036814
Category	Term
GOTERM_BP_ALL	GO:0071229~cellular response to acid chemical
GOTERM_BP_ALL	GO:0071300~cellular response to retinoic acid
GOTERM_BP_ALL	GO:0032526~response to retinoic acid
Annotation Cluster 153	Enrichment Score: 0.846001510025847
Category	Term
GOTERM_BP_ALL	GO:0030225~macrophage differentiation
GOTERM_BP_ALL	GO:0002262~myeloid cell homeostasis
GOTERM_BP_ALL	GO:1904036~negative regulation of epithelial cell apoptotic process
GOTERM_BP_ALL	GO:1904019~epithelial cell apoptotic process
GOTERM_BP_ALL	GO:1904035~regulation of epithelial cell apoptotic process
GOTERM_BP_ALL	GO:0030218~erythrocyte differentiation
GOTERM_BP_ALL	GO:0034101~erythrocyte homeostasis
Annotation Cluster 154	Enrichment Score: 0.84587242377765
Category	Term
GOTERM_BP_ALL	GO:0009566~fertilization
GOTERM_BP_ALL	GO:0007338~single fertilization
GOTERM_BP_ALL	GO:0048477~oogenesis
GOTERM_BP_ALL	GO:0007292~female gamete generation
GOTERM_BP_ALL	GO:0007281~germ cell development
GOTERM_BP_ALL	GO:0007276~gamete generation
GOTERM_BP_ALL	GO:0022412~cellular process involved in reproduction in multicellular organism
Annotation Cluster 155	Enrichment Score: 0.8368124959811799
Category	Term
GOTERM_BP_ALL	GO:0032409~regulation of transporter activity
GOTERM_BP_ALL	GO:0022898~regulation of transmembrane transporter activity
GOTERM_BP_ALL	GO:0050795~regulation of behavior
GOTERM_BP_ALL	GO:2001257~regulation of cation channel activity
GOTERM_BP_ALL	GO:0032412~regulation of ion transmembrane transporter activity
Annotation Cluster 156	Enrichment Score: 0.8241047612144611
Category	Term
GOTERM_BP_ALL	GO:0006979~response to oxidative stress
GOTERM_BP_ALL	GO:0042542~response to hydrogen peroxide
GOTERM_BP_ALL	GO:0000302~response to reactive oxygen species
Annotation Cluster 157	Enrichment Score: 0.7977590250018755
Category	Term
GOTERM_BP_ALL	GO:1903578~regulation of ATP metabolic process
GOTERM_BP_ALL	GO:0009135~purine nucleoside diphosphate metabolic process
GOTERM_BP_ALL	GO:0009179~purine ribonucleoside diphosphate metabolic process
GOTERM_BP_ALL	GO:0044723~single-organism carbohydrate metabolic process
GOTERM_BP_ALL	GO:0043470~regulation of carbohydrate catabolic process

GOTERM_BP_ALL	GO:0006090~pyruvate metabolic process
GOTERM_BP_ALL	GO:0051196~regulation of coenzyme metabolic process
GOTERM_BP_ALL	GO:0009185~ribonucleoside diphosphate metabolic process
GOTERM_BP_ALL	GO:1900542~regulation of purine nucleotide metabolic process
GOTERM_BP_ALL	GO:0006140~regulation of nucleotide metabolic process
GOTERM_BP_ALL	GO:0051193~regulation of cofactor metabolic process
GOTERM_BP_ALL	GO:0009132~nucleoside diphosphate metabolic process
GOTERM_BP_ALL	GO:0043467~regulation of generation of precursor metabolites and energy
GOTERM_BP_ALL	GO:0005996~monosaccharide metabolic process
GOTERM_BP_ALL	GO:0046031~ADP metabolic process
GOTERM_BP_ALL	GO:0046496~nicotinamide nucleotide metabolic process
GOTERM_BP_ALL	GO:0019362~pyridine nucleotide metabolic process
GOTERM_BP_ALL	GO:0019637~organophosphate metabolic process
GOTERM_BP_ALL	GO:0072524~pyridine-containing compound metabolic process
GOTERM_BP_ALL	GO:0019318~hexose metabolic process
GOTERM_BP_ALL	GO:0005975~carbohydrate metabolic process
GOTERM_BP_ALL	GO:0006110~regulation of glycolytic process
GOTERM_BP_ALL	GO:0006733~oxidoreduction coenzyme metabolic process
GOTERM_BP_ALL	GO:0006109~regulation of carbohydrate metabolic process
GOTERM_BP_ALL	GO:0006006~glucose metabolic process
GOTERM_BP_ALL	GO:0045912~negative regulation of carbohydrate metabolic process
GOTERM_BP_ALL	GO:0044262~cellular carbohydrate metabolic process
GOTERM_BP_ALL	GO:0044724~single-organism carbohydrate catabolic process
GOTERM_BP_ALL	GO:0006096~glycolytic process
GOTERM_BP_ALL	GO:0006757~ATP generation from ADP
GOTERM_BP_ALL	GO:0045913~positive regulation of carbohydrate metabolic process
GOTERM_BP_ALL	GO:0010675~regulation of cellular carbohydrate metabolic process
GOTERM_BP_ALL	GO:0050730~regulation of peptidyl-tyrosine phosphorylation
GOTERM_BP_ALL	GO:0009126~purine nucleoside monophosphate metabolic process
GOTERM_BP_ALL	GO:0009167~purine ribonucleoside monophosphate metabolic process
GOTERM_BP_ALL	GO:0006165~nucleoside diphosphate phosphorylation
GOTERM_BP_ALL	GO:0045981~positive regulation of nucleotide metabolic process
GOTERM_BP_ALL	GO:0046939~nucleotide phosphorylation
GOTERM_BP_ALL	GO:1900544~positive regulation of purine nucleotide metabolic process
GOTERM_BP_ALL	GO:0006163~purine nucleotide metabolic process
GOTERM_BP_ALL	GO:0016052~carbohydrate catabolic process
GOTERM_BP_ALL	GO:0009161~ribonucleoside monophosphate metabolic process
GOTERM_BP_ALL	GO:0009123~nucleoside monophosphate metabolic process
GOTERM_BP_ALL	GO:0009117~nucleotide metabolic process
GOTERM_BP_ALL	GO:0072521~purine-containing compound metabolic process
GOTERM_BP_ALL	GO:0006753~nucleoside phosphate metabolic process
GOTERM_BP_ALL	GO:0046034~ATP metabolic process
GOTERM_BP_ALL	GO:0010906~regulation of glucose metabolic process
GOTERM_BP_ALL	GO:0009150~purine ribonucleotide metabolic process
GOTERM_BP_ALL	GO:0055086~nucleobase-containing small molecule metabolic process
GOTERM_BP_ALL	GO:0009205~purine ribonucleoside triphosphate metabolic process
GOTERM_BP_ALL	GO:0009144~purine nucleoside triphosphate metabolic process
GOTERM_BP_ALL	GO:0009199~ribonucleoside triphosphate metabolic process
GOTERM_BP_ALL	GO:0009259~ribonucleotide metabolic process
GOTERM_BP_ALL	GO:0019693~ribose phosphate metabolic process
GOTERM_BP_ALL	GO:0009141~nucleoside triphosphate metabolic process

GOTERM_BP_ALL	GO:0006732~coenzyme metabolic process
GOTERM_BP_ALL	GO:0006091~generation of precursor metabolites and energy
GOTERM_BP_ALL	GO:0044712~single-organism catabolic process
GOTERM_BP_ALL	GO:0015980~energy derivation by oxidation of organic compounds
GOTERM_BP_ALL	GO:0044281~small molecule metabolic process
GOTERM_BP_ALL	GO:0032787~monocarboxylic acid metabolic process
GOTERM_BP_ALL	GO:0043436~oxoacid metabolic process
GOTERM_BP_ALL	GO:0019752~carboxylic acid metabolic process
GOTERM_BP_ALL	GO:0006082~organic acid metabolic process
GOTERM_BP_ALL	GO:1901135~carbohydrate derivative metabolic process
GOTERM_BP_ALL	GO:0051186~cofactor metabolic process
GOTERM_BP_ALL	GO:0055114~oxidation-reduction process
Annotation Cluster 158	Enrichment Score: 0.7819598211594245
Category	Term
GOTERM_BP_ALL	GO:0050920~regulation of chemotaxis
GOTERM_BP_ALL	GO:0050921~positive regulation of chemotaxis
GOTERM_BP_ALL	GO:1902622~regulation of neutrophil migration
GOTERM_BP_ALL	GO:0043277~apoptotic cell clearance
GOTERM_BP_ALL	GO:0050900~leukocyte migration
GOTERM_BP_ALL	GO:0002685~regulation of leukocyte migration
GOTERM_BP_ALL	GO:0097529~myeloid leukocyte migration
GOTERM_BP_ALL	GO:0006909~phagocytosis
GOTERM_BP_ALL	GO:0002690~positive regulation of leukocyte chemotaxis
GOTERM_BP_ALL	GO:0032103~positive regulation of response to external stimulus
GOTERM_BP_ALL	GO:0002688~regulation of leukocyte chemotaxis
GOTERM_BP_ALL	GO:0060326~cell chemotaxis
GOTERM_BP_ALL	GO:0002687~positive regulation of leukocyte migration
GOTERM_BP_ALL	GO:0030595~leukocyte chemotaxis
Annotation Cluster 159	Enrichment Score: 0.7774483433735004
Category	Term
GOTERM_BP_ALL	GO:0060969~negative regulation of gene silencing
GOTERM_BP_ALL	GO:0060968~regulation of gene silencing
GOTERM_BP_ALL	GO:0016458~gene silencing
Annotation Cluster 160	Enrichment Score: 0.7762443138570287
Category	Term
GOTERM_BP_ALL	GO:0032606~type I interferon production
GOTERM_BP_ALL	GO:0032608~interferon-beta production
GOTERM_BP_ALL	GO:0032479~regulation of type I interferon production
GOTERM_BP_ALL	GO:0032648~regulation of interferon-beta production
GOTERM_BP_ALL	GO:0001816~cytokine production
GOTERM_BP_ALL	GO:0001817~regulation of cytokine production
Annotation Cluster 161	Enrichment Score: 0.7679087538060161
Category	Term
GOTERM_BP_ALL	GO:0009566~fertilization
GOTERM_BP_ALL	GO:0007565~female pregnancy
GOTERM_BP_ALL	GO:0044706~multi-multicellular organism process

Annotation Cluster 162	Enrichment Score: 0.7624505122664094
Category	Term
GOTERM_BP_ALL	GO:0046189~phenol-containing compound biosynthetic process
GOTERM_BP_ALL	GO:1901617~organic hydroxy compound biosynthetic process
GOTERM_BP_ALL	GO:1901615~organic hydroxy compound metabolic process
GOTERM_BP_ALL	GO:0018958~phenol-containing compound metabolic process
Annotation Cluster 163	Enrichment Score: 0.7503871878334597
Category	Term
GOTERM_BP_ALL	GO:0018107~peptidyl-threonine phosphorylation
GOTERM_BP_ALL	GO:0018210~peptidyl-threonine modification
GOTERM_BP_ALL	GO:0018209~peptidyl-serine modification
GOTERM_BP_ALL	GO:0018105~peptidyl-serine phosphorylation
Annotation Cluster 164	Enrichment Score: 0.7247209328081105
Category	Term
GOTERM_BP_ALL	GO:0000082~G1/S transition of mitotic cell cycle
GOTERM_BP_ALL	GO:0045727~positive regulation of translation
GOTERM_BP_ALL	GO:0034250~positive regulation of cellular amide metabolic process
GOTERM_BP_ALL	GO:0035196~production of miRNAs involved in gene silencing by miRNA
GOTERM_BP_ALL	GO:0070918~production of small RNA involved in gene silencing by RNA
GOTERM_BP_ALL	GO:0031050~dsRNA fragmentation
GOTERM_BP_ALL	GO:0071359~cellular response to dsRNA
GOTERM_BP_ALL	GO:0043331~response to dsRNA
GOTERM_BP_ALL	GO:0016458~gene silencing
GOTERM_BP_ALL	GO:0017148~negative regulation of translation
GOTERM_BP_ALL	GO:0034249~negative regulation of cellular amide metabolic process
GOTERM_BP_ALL	GO:0035195~gene silencing by miRNA
GOTERM_BP_ALL	GO:0035194~posttranscriptional gene silencing by RNA
GOTERM_BP_ALL	GO:0016441~posttranscriptional gene silencing
GOTERM_BP_ALL	GO:0031047~gene silencing by RNA
Annotation Cluster 165	Enrichment Score: 0.7074469790291038
Category	Term
GOTERM_BP_ALL	GO:1903201~regulation of oxidative stress-induced cell death
GOTERM_BP_ALL	GO:0034599~cellular response to oxidative stress
GOTERM_BP_ALL	GO:1900407~regulation of cellular response to oxidative stress
GOTERM_BP_ALL	GO:0036473~cell death in response to oxidative stress
GOTERM_BP_ALL	GO:1902882~regulation of response to oxidative stress
Annotation Cluster 166	Enrichment Score: 0.6971883212428183
Category	Term
GOTERM_BP_ALL	GO:0031669~cellular response to nutrient levels
GOTERM_BP_ALL	GO:0009267~cellular response to starvation
GOTERM_BP_ALL	GO:0042594~response to starvation
Annotation Cluster 167	Enrichment Score: 0.6689723721096669
Category	Term
GOTERM_BP_ALL	GO:0046328~regulation of JNK cascade
GOTERM_BP_ALL	GO:0007254~JNK cascade
GOTERM_BP_ALL	GO:0032872~regulation of stress-activated MAPK cascade

GOTERM_BP_ALL	GO:0070302~regulation of stress-activated protein kinase signaling cascade
GOTERM_BP_ALL	GO:0051403~stress-activated MAPK cascade
GOTERM_BP_ALL	GO:0031098~stress-activated protein kinase signaling cascade
GOTERM_BP_ALL	GO:0046330~positive regulation of JNK cascade
GOTERM_BP_ALL	GO:0032874~positive regulation of stress-activated MAPK cascade
GOTERM_BP_ALL	GO:0070304~positive regulation of stress-activated protein kinase signaling cascade
Annotation Cluster 168	Enrichment Score: 0.6685013142912606
Category	Term
GOTERM_BP_ALL	GO:0006629~lipid metabolic process
GOTERM_BP_ALL	GO:0008610~lipid biosynthetic process
GOTERM_BP_ALL	GO:0044255~cellular lipid metabolic process
Annotation Cluster 169	Enrichment Score: 0.6466351724600641
Category	Term
GOTERM_BP_ALL	GO:0008015~blood circulation
GOTERM_BP_ALL	GO:0003013~circulatory system process
GOTERM_BP_ALL	GO:0060047~heart contraction
GOTERM_BP_ALL	GO:0003015~heart process
Annotation Cluster 170	Enrichment Score: 0.6264524558604854
Category	Term
GOTERM_BP_ALL	GO:0002366~leukocyte activation involved in immune response
GOTERM_BP_ALL	GO:0002263~cell activation involved in immune response
GOTERM_BP_ALL	GO:0002252~immune effector process
GOTERM_BP_ALL	GO:0002443~leukocyte mediated immunity
GOTERM_BP_ALL	GO:0006952~defense response
GOTERM_BP_ALL	GO:0050776~regulation of immune response
GOTERM_BP_ALL	GO:0002757~immune response-activating signal transduction
GOTERM_BP_ALL	GO:0050778~positive regulation of immune response
GOTERM_BP_ALL	GO:0002429~immune response-activating cell surface receptor signaling pathway
GOTERM_BP_ALL	GO:0002764~immune response-regulating signaling pathway
GOTERM_BP_ALL	GO:0031347~regulation of defense response
GOTERM_BP_ALL	GO:0050851~antigen receptor-mediated signaling pathway
GOTERM_BP_ALL	GO:0002768~immune response-regulating cell surface receptor signaling pathway
GOTERM_BP_ALL	GO:0002253~activation of immune response
GOTERM_BP_ALL	GO:0009607~response to biotic stimulus
GOTERM_BP_ALL	GO:0050853~B cell receptor signaling pathway
GOTERM_BP_ALL	GO:0045087~innate immune response
GOTERM_BP_ALL	GO:0006955~immune response
GOTERM_BP_ALL	GO:0002460~adaptive immune response based on somatic recombination of immune receptors built from immunoglobulin superfamily domains
GOTERM_BP_ALL	GO:0045088~regulation of innate immune response
GOTERM_BP_ALL	GO:0051707~response to other organism
GOTERM_BP_ALL	GO:0043207~response to external biotic stimulus
GOTERM_BP_ALL	GO:0002758~innate immune response-activating signal transduction
GOTERM_BP_ALL	GO:0002218~activation of innate immune response
GOTERM_BP_ALL	GO:0009617~response to bacterium

GOTERM_BP_ALL	GO:0045089~positive regulation of innate immune response
GOTERM_BP_ALL	GO:0031349~positive regulation of defense response
GOTERM_BP_ALL	GO:0002250~adaptive immune response
GOTERM_BP_ALL	GO:0006959~humoral immune response
Annotation Cluster 171	Enrichment Score: 0.5941209905045322
Category	Term
GOTERM_BP_ALL	GO:0002285~lymphocyte activation involved in immune response
GOTERM_BP_ALL	GO:0002703~regulation of leukocyte mediated immunity
GOTERM_BP_ALL	GO:0002443~leukocyte mediated immunity
GOTERM_BP_ALL	GO:0050778~positive regulation of immune response
GOTERM_BP_ALL	GO:0002822~regulation of adaptive immune response based on somatic recombination of immune receptors built from immunoglobulin superfamily domains
GOTERM_BP_ALL	GO:0002819~regulation of adaptive immune response
GOTERM_BP_ALL	GO:0002706~regulation of lymphocyte mediated immunity
GOTERM_BP_ALL	GO:0002460~adaptive immune response based on somatic recombination of immune receptors built from immunoglobulin superfamily domains
GOTERM_BP_ALL	GO:0002449~lymphocyte mediated immunity
GOTERM_BP_ALL	GO:0002250~adaptive immune response
Annotation Cluster 172	Enrichment Score: 0.5843041661961808
Category	Term
GOTERM_BP_ALL	GO:0032602~chemokine production
GOTERM_BP_ALL	GO:0032642~regulation of chemokine production
GOTERM_BP_ALL	GO:0032722~positive regulation of chemokine production
GOTERM_BP_ALL	GO:0032755~positive regulation of interleukin-6 production
GOTERM_BP_ALL	GO:0032680~regulation of tumor necrosis factor production
GOTERM_BP_ALL	GO:0032760~positive regulation of tumor necrosis factor production
GOTERM_BP_ALL	GO:0071706~tumor necrosis factor superfamily cytokine production
GOTERM_BP_ALL	GO:1903555~regulation of tumor necrosis factor superfamily cytokine production
GOTERM_BP_ALL	GO:1903557~positive regulation of tumor necrosis factor superfamily cytokine production
GOTERM_BP_ALL	GO:0001819~positive regulation of cytokine production
GOTERM_BP_ALL	GO:0001816~cytokine production
GOTERM_BP_ALL	GO:0001817~regulation of cytokine production
GOTERM_BP_ALL	GO:0032635~interleukin-6 production
GOTERM_BP_ALL	GO:0032640~tumor necrosis factor production
GOTERM_BP_ALL	GO:0032675~regulation of interleukin-6 production
Annotation Cluster 173	Enrichment Score: 0.56259334801895
Category	Term
GOTERM_BP_ALL	GO:0015672~monovalent inorganic cation transport
GOTERM_BP_ALL	GO:0071804~cellular potassium ion transport
GOTERM_BP_ALL	GO:0071805~potassium ion transmembrane transport
GOTERM_BP_ALL	GO:0006813~potassium ion transport
Annotation Cluster 174	Enrichment Score: 0.5185923868958442
Category	Term
GOTERM_BP_ALL	GO:0032984~macromolecular complex disassembly

GOTERM_BP_ALL	GO:0043244~regulation of protein complex disassembly
GOTERM_BP_ALL	GO:0043624~cellular protein complex disassembly
GOTERM_BP_ALL	GO:0043241~protein complex disassembly
Annotation Cluster 175	Enrichment Score: 0.4945374685345561
Category	Term
GOTERM_BP_ALL	GO:1902749~regulation of cell cycle G2/M phase transition
GOTERM_BP_ALL	GO:0010389~regulation of G2/M transition of mitotic cell cycle
GOTERM_BP_ALL	GO:0000086~G2/M transition of mitotic cell cycle
GOTERM_BP_ALL	GO:0044839~cell cycle G2/M phase transition
Annotation Cluster 176	Enrichment Score: 0.4150920120706421
Category	Term
GOTERM_BP_ALL	GO:0045727~positive regulation of translation
GOTERM_BP_ALL	GO:0034250~positive regulation of cellular amide metabolic process
GOTERM_BP_ALL	GO:0034248~regulation of cellular amide metabolic process
GOTERM_BP_ALL	GO:0006417~regulation of translation
GOTERM_BP_ALL	GO:0006412~translation
GOTERM_BP_ALL	GO:0043604~amide biosynthetic process
GOTERM_BP_ALL	GO:0043043~peptide biosynthetic process
GOTERM_BP_ALL	GO:0006518~peptide metabolic process
GOTERM_BP_ALL	GO:0043603~cellular amide metabolic process
GOTERM_BP_ALL	GO:0010608~posttranscriptional regulation of gene expression
GOTERM_BP_ALL	GO:1901564~organonitrogen compound metabolic process
GOTERM_BP_ALL	GO:1901566~organonitrogen compound biosynthetic process
GOTERM_BP_ALL	GO:0017148~negative regulation of translation
GOTERM_BP_ALL	GO:0034249~negative regulation of cellular amide metabolic process
Annotation Cluster 177	Enrichment Score: 0.41346667047514113
Category	Term
GOTERM_BP_ALL	GO:0031347~regulation of defense response
GOTERM_BP_ALL	GO:0050728~negative regulation of inflammatory response
GOTERM_BP_ALL	GO:0031348~negative regulation of defense response
GOTERM_BP_ALL	GO:0032102~negative regulation of response to external stimulus
GOTERM_BP_ALL	GO:0050727~regulation of inflammatory response
Annotation Cluster 178	Enrichment Score: 0.3811462164125748
Category	Term
GOTERM_BP_ALL	GO:0045862~positive regulation of proteolysis
GOTERM_BP_ALL	GO:0070372~regulation of ERK1 and ERK2 cascade
GOTERM_BP_ALL	GO:0070371~ERK1 and ERK2 cascade
GOTERM_BP_ALL	GO:0070374~positive regulation of ERK1 and ERK2 cascade
Annotation Cluster 179	Enrichment Score: 0.3806722116552417
Category	Term
GOTERM_BP_ALL	GO:0031333~negative regulation of protein complex assembly
GOTERM_BP_ALL	GO:0070897~DNA-templated transcriptional preinitiation complex assembly
GOTERM_BP_ALL	GO:1903902~positive regulation of viral life cycle
GOTERM_BP_ALL	GO:0048524~positive regulation of viral process
GOTERM_BP_ALL	GO:0019079~viral genome replication
GOTERM_BP_ALL	GO:0043902~positive regulation of multi-organism process

GOTERM_BP_ALL	GO:0016032~viral process
GOTERM_BP_ALL	GO:1903900~regulation of viral life cycle
GOTERM_BP_ALL	GO:0044764~multi-organism cellular process
GOTERM_BP_ALL	GO:0043900~regulation of multi-organism process
GOTERM_BP_ALL	GO:0044403~symbiosis, encompassing mutualism through parasitism
GOTERM_BP_ALL	GO:0044419~interspecies interaction between organisms
GOTERM_BP_ALL	GO:0009615~response to virus
GOTERM_BP_ALL	GO:0019058~viral life cycle
GOTERM_BP_ALL	GO:0050792~regulation of viral process
GOTERM_BP_ALL	GO:0043903~regulation of symbiosis, encompassing mutualism through parasitism
Annotation Cluster 180	Enrichment Score: 0.3722673512565431
Category	Term
GOTERM_BP_ALL	GO:0007601~visual perception
GOTERM_BP_ALL	GO:0050953~sensory perception of light stimulus
GOTERM_BP_ALL	GO:0007600~sensory perception
Annotation Cluster 181	Enrichment Score: 0.34441239425789444
Category	Term
GOTERM_BP_ALL	GO:0006644~phospholipid metabolic process
GOTERM_BP_ALL	GO:1901615~organic hydroxy compound metabolic process
GOTERM_BP_ALL	GO:0006066~alcohol metabolic process
GOTERM_BP_ALL	GO:0008654~phospholipid biosynthetic process
GOTERM_BP_ALL	GO:0090407~organophosphate biosynthetic process
Annotation Cluster 182	Enrichment Score: 0.32115815963538275
Category	Term
GOTERM_BP_ALL	GO:0006403~RNA localization
GOTERM_BP_ALL	GO:0015931~nucleobase-containing compound transport
GOTERM_BP_ALL	GO:0050657~nucleic acid transport
GOTERM_BP_ALL	GO:0050658~RNA transport
GOTERM_BP_ALL	GO:0051236~establishment of RNA localization
Annotation Cluster 183	Enrichment Score: 0.30254801956009814
Category	Term
GOTERM_BP_ALL	GO:1904064~positive regulation of cation transmembrane transport
GOTERM_BP_ALL	GO:0034767~positive regulation of ion transmembrane transport
GOTERM_BP_ALL	GO:0034764~positive regulation of transmembrane transport
Annotation Cluster 184	Enrichment Score: 0.29203327291829345
Category	Term
GOTERM_BP_ALL	GO:0006644~phospholipid metabolic process
GOTERM_BP_ALL	GO:0046486~glycerolipid metabolic process
GOTERM_BP_ALL	GO:0006650~glycerophospholipid metabolic process
Annotation Cluster 185	Enrichment Score: 0.24075939762448328
Category	Term
GOTERM_BP_ALL	GO:0006457~protein folding
GOTERM_BP_ALL	GO:0007188~adenylate cyclase-modulating G-protein coupled receptor signaling pathway

GOTERM_BP_ALL	GO:0007187~G-protein coupled receptor signaling pathway, coupled to cyclic nucleotide second messenger
Annotation Cluster 186	Enrichment Score: 0.23973962167126972
Category	Term
GOTERM_BP_ALL	GO:0042113~B cell activation
GOTERM_BP_ALL	GO:0050871~positive regulation of B cell activation
GOTERM_BP_ALL	GO:0002460~adaptive immune response based on somatic recombination of immune receptors built from immunoglobulin superfamily domains
GOTERM_BP_ALL	GO:0050864~regulation of B cell activation
GOTERM_BP_ALL	GO:0002250~adaptive immune response
Annotation Cluster 187	Enrichment Score: 0.14615944222136285
Category	Term
GOTERM_BP_ALL	GO:0032496~response to lipopolysaccharide
GOTERM_BP_ALL	GO:0002237~response to molecule of bacterial origin
GOTERM_BP_ALL	GO:0009617~response to bacterium
Annotation Cluster 188	Enrichment Score: 0.11781501682953328
Category	Term
GOTERM_BP_ALL	GO:0042254~ribosome biogenesis
GOTERM_BP_ALL	GO:0022613~ribonucleoprotein complex biogenesis
GOTERM_BP_ALL	GO:0034660~ncRNA metabolic process
GOTERM_BP_ALL	GO:0034470~ncRNA processing
Annotation Cluster 189	Enrichment Score: 0.10465604094187994
Category	Term
GOTERM_BP_ALL	GO:0022613~ribonucleoprotein complex biogenesis
GOTERM_BP_ALL	GO:0022618~ribonucleoprotein complex assembly
GOTERM_BP_ALL	GO:0071826~ribonucleoprotein complex subunit organization
Annotation Cluster 190	Enrichment Score: 0.0824014777718883
Category	Term
GOTERM_BP_ALL	GO:0009100~glycoprotein metabolic process
GOTERM_BP_ALL	GO:1901137~carbohydrate derivative biosynthetic process
GOTERM_BP_ALL	GO:0009101~glycoprotein biosynthetic process
