# Supplementary material for: Meta-analysis of 46,000 germline de novo mutations linked to human inherited disease
Source: Hum Genomics. 2024 Feb 23;18:20. doi: 10.1186/s40246-024-00587-8 (PMC10885371; doi:10.1186/s40246-024-00587-8)

Additional file 4. REVEL scores for the two groups of HGMD disease-causing missense mutations (5,307 DNM versus 32,605 non-DNM). Purple bar indicates the proportion of dataset overlap between DNM and non-DNM for a specific range of scores; red indicates enrichment of the DNM; blue denotes enrichment of the non-DNM set.

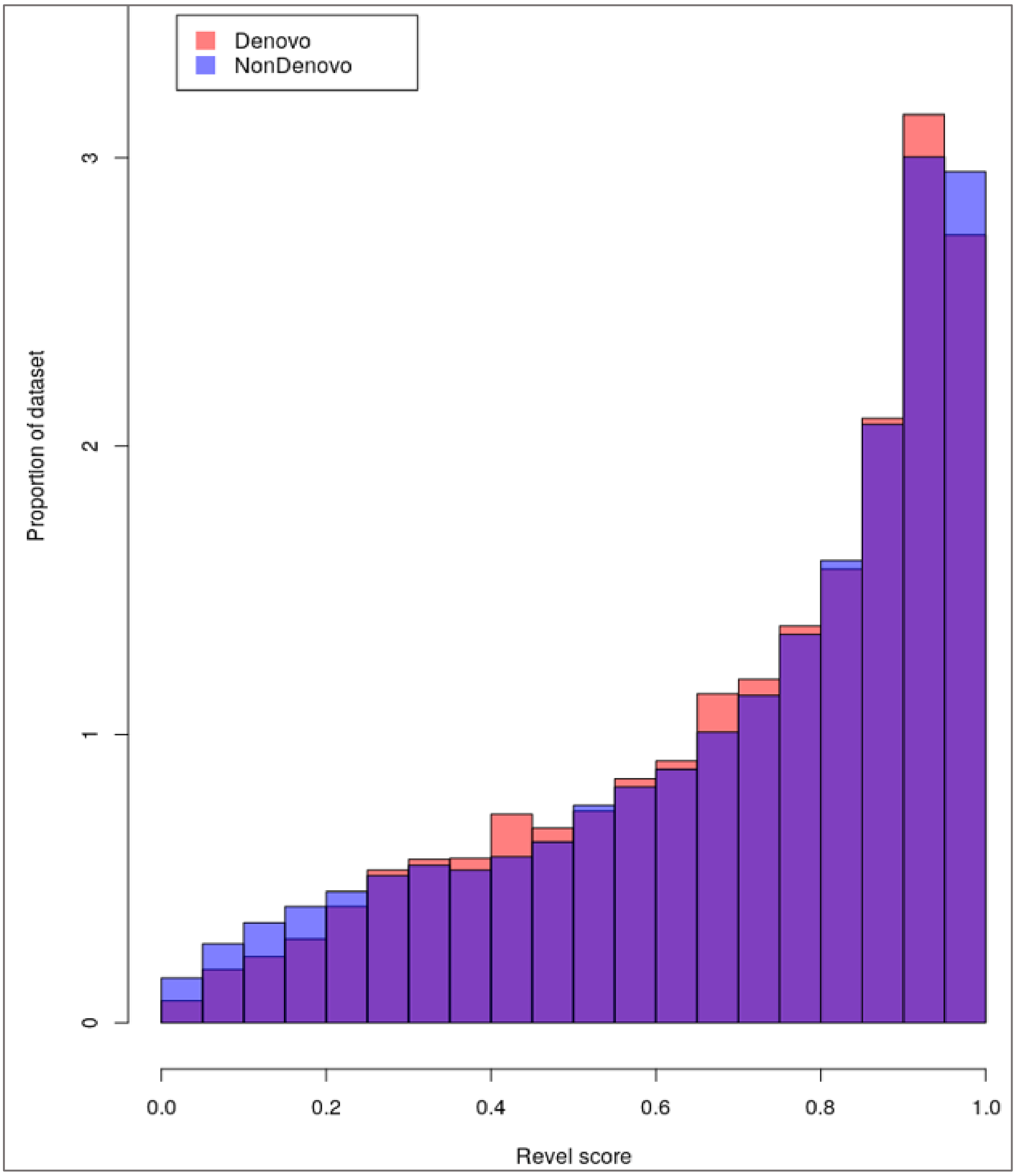

Supplement: Supplementary file 3 — Supplementary Material 3 [file 40246_2024_587_MOESM3_ESM.pdf]
