# Supplementary material for: Meta-analysis of 46,000 germline de novo mutations linked to human inherited disease
Source: Hum Genomics. 2024 Feb 23;18:20. doi: 10.1186/s40246-024-00587-8 (PMC10885371; doi:10.1186/s40246-024-00587-8)
Supplement: Supplementary file 4 — Supplementary Material 4 [file 40246_2024_587_MOESM4_ESM.pdf]

Additional file 2. Relative mutability of 187 genes enriched for DNMs.

Gene Symbol	Frequency of DMNs	Coding length (bp)	Relative mutability (DNM frequency/coding length)*100	Relative mutability (DNM frequency/expected Genovo missense from Bethune et al. 2022
H3-3A	29	411	7.056	0.300
DDX3X	134	1986	6.747	
CSNK2B	43	648	6.636	0.343
PURA	61	969	6.295	
ZC4H2	40	675	5.926	
STXBP1	95	1812	5.243	
SCN1A	311	6030	5.158	
SATB2	104	2202	4.723	
H3-3B	19	411	4.623	0.189
TUBA1A	60	1356	4.425	0.216
SLC35A2	49	1182	4.146	
CTNNB1	94	2346	4.007	0.207
VAMP2	14	351	3.989	0.202
GNAO1	42	1065	3.944	0.177
MECP2	57	1461	3.901	
DYRK1A	84	2292	3.665	
GATAD2B	64	1782	3.591	0.181
WDR45	39	1086	3.591	
SLC6A1	64	1800	3.556	0.170
H1-4	22	660	3.333	
KCNQ2	86	2619	3.284	0.155
SOX11	40	1326	3.017	
ARID1B	209	6999	2.986	
SOX10	41	1401	2.926	0.131
ACTB	33	1128	2.926	
SYNGAP1	117	4032	2.902	
H4C5	9	312	2.885	
PCDH19	95	3447	2.756	
GABRB3	39	1422	2.743	0.142
NR2F1	34	1272	2.673	0.113
HNRNPU	65	2478	2.623	
SCN2A	154	6018	2.559	
PTEN	31	1212	2.558	0.146
FBXO11	71	2784	2.550	0.158
EBF3	41	1656	2.476	
RB1	68	2787	2.440	0.145
SLC2A1	36	1479	2.434	0.111
RAC3	14	579	2.418	0.105
FOXP1	49	2034	2.409	
NAA10	17	708	2.401	
FOXG1	35	1470	2.381	
EIF5A	11	465	2.366	0.116
PAFAH1B1	29	1233	2.352	0.124

SHANK3	127	5421	2.343	
PHEX	52	2250	2.311	
CDKL5	71	3093	2.296	
GABRB2	35	1539	2.274	
TBL1XR1	35	1545	2.265	0.136
PBX1	29	1293	2.243	0.120
HNRNPK	31	1395	2.222	
ACTA1	25	1134	2.205	0.085
WDR26	43	1986	2.165	
NF1	179	8457	2.117	
TP53	25	1182	2.115	0.098
snap25b	13	621	2.093	0.0972
ANKRD11	167	7992	2.090	0.102
RAC1	13	636	2.044	
GRIN2B	91	4455	2.043	0.097
TLK2	45	2253	1.997	0.111
ACTG1	22	1128	1.950	0.079
DEAF1	33	1698	1.943	0.099
ATP1A3	59	3042	1.940	
PPP2CA	18	930	1.935	0.098
DNMT3A	53	2739	1.935	
CDC42	11	576	1.910	
GABRA1	26	1371	1.896	
IGLL5	12	645	1.860	0.132
CREBBP	136	7329	1.856	0.093
HDAC8	21	1134	1.852	
ARF3	10	546	1.832	0.088
POGZ	77	4233	1.819	0.095
NUS1	16	882	1.814	0.114
INS	6	333	1.802	0.097
MYT1L	64	3555	1.800	
HNF1B	30	1674	1.792	
TCF4	36	2016	1.786	0.090
CSNK2A1	21	1176	1.786	
GNB1	18	1023	1.760	0.081
YWHAG	13	744	1.747	0.074
EFTUD2	51	2919	1.747	0.089
RPS26	6	348	1.724	
GNAI1	18	1065	1.690	
PLP1	14	834	1.679	
TWIST1	10	609	1.642	0.080
PHF6	18	1098	1.639	
GABRG2	23	1404	1.638	
NFIX	25	1533	1.631	
PSMD12	22	1371	1.605	0.089
H4C3	5	312	1.603	
H4C11	5	312	1.603	
NR5A1	22	1386	1.587	0.080
FOXF1	18	1140	1.579	0.072

HBB	7	444	1.577	0.088
SYT1	20	1269	1.576	0.088
ZBTB20	35	2226	1.572	
LAT	11	702	1.567	0.078
PUF60	26	1680	1.548	0.076
KMT5B	41	2658	1.543	0.085
CHD2	84	5487	1.531	0.083
OTC	16	1065	1.502	
MED13L	99	6633	1.493	0.079
COL4A1	74	5010	1.477	0.074
AHDC1	71	4812	1.475	
SOX2	14	954	1.468	
TBR1	30	2049	1.464	0.070
NAA15	38	2601	1.461	0.091
TCEAL1	7	480	1.458	
PDHA1	17	1173	1.449	
PMP22	7	483	1.449	0.076
EHMT1	56	3897	1.437	0.065
EEF1A2	20	1392	1.437	0.066
KCNB1	37	2577	1.436	0.070
MEIS2	17	1185	1.435	
MEF2C	20	1422	1.406	0.076
TSC2	75	5424	1.383	0.066
COL1A1	60	4395	1.365	0.062
CAMK2A	20	1470	1.361	
SMC1A	50	3702	1.351	
MITF	17	1260	1.349	
ASXL3	91	6747	1.349	0.079
PPP3CA	21	1566	1.341	0.074
TAOK1	40	3006	1.331	0.071
GATA2	19	1443	1.317	0.060
SCN8A	78	5943	1.312	
EIF4A2	16	1224	1.307	0.069
LMNA	26	1995	1.303	0.057
RRAS2	8	615	1.301	0.069
QRICH1	30	2331	1.287	
WAC	25	1944	1.286	0.075
PAX2	16	1254	1.276	
FBXW7	27	2124	1.271	
PRRT2	13	1023	1.271	0.065
FGFR1	31	2469	1.256	0.063
IQSEC2	56	4467	1.254	
MSL3	14	1119	1.251	
NKX2-1	15	1206	1.244	0.057
FBN1	107	8616	1.242	0.063
FOXL2	14	1131	1.238	
ACTG2	14	1131	1.238	0.060
NFIB	18	1485	1.212	0.071
NR2F2	15	1245	1.205	0.056

POU3F3	18	1503	1.198	
TUBB2B	16	1338	1.196	0.061
SOX4	17	1425	1.193	
SPAST	22	1851	1.189	0.065
ZIC2	19	1599	1.188	0.062
stxbp1tv2	21	1785	1.176	0.057
DNM1L	26	2211	1.176	0.064
UBE3A	30	2559	1.172	
COL2A1	52	4464	1.165	0.055
TCF20	68	5883	1.156	
msl3tv1	18	1566	1.149	
OTX2	10	870	1.149	
SET	10	873	1.145	
COL6A2	35	3060	1.144	0.047
ATP6V1A	21	1854	1.133	0.060
IRF2BPL	27	2391	1.129	
RALA	7	621	1.127	0.064
YY1	14	1245	1.124	0.057
SMARCB1	13	1158	1.123	
CASK	31	2766	1.121	
RPL5	10	894	1.119	0.057
ADNP	37	3309	1.118	
NR4A2	20	1797	1.113	0.053
CHD7	100	8994	1.112	0.062
DPF2	13	1176	1.105	0.052
IGF2	6	543	1.105	0.045
TAB2	23	2082	1.105	
GRIN1	31	2817	1.100	0.048
ZEB2	40	3645	1.097	
CLCN4	25	2283	1.095	
DHDDS	11	1005	1.095	
GRIA2	29	2652	1.094	0.059
KMT2B	89	8148	1.092	0.055
YWHAZ	8	738	1.084	
KMT2A	129	11919	1.082	0.058
NOVA2	16	1479	1.082	0.058
SSR4	6	555	1.081	
ANK1	61	5643	1.081	0.051
HMGB1	7	648	1.080	0.083
SHH	15	1389	1.080	0.057
RFX3	24	2250	1.067	
KAT6A	64	6015	1.064	0.057
KRAS	6	567	1.058	0.062
ZMYND11	19	1809	1.050	
GATA3	14	1335	1.049	0.048
CUL3	24	2307	1.040	0.057
